# Seeking patterns of antibiotic resistance in ATLAS, an open, raw MIC database with patient metadata

**DOI:** 10.1038/s41467-022-30635-7

**Published:** 2022-05-25

**Authors:** Pablo Catalán, Emily Wood, Jessica M. A. Blair, Ivana Gudelj, Jonathan R. Iredell, Robert E. Beardmore

**Affiliations:** 1 Biosciences, College of Life and Environmental Sciences, University of Exeter, Exeter, EX4 4QD UK; 2 Grupo Interdisciplinar de Sistemas Complejos, Departamento de Matemáticas, Universidad Carlos III de Madrid, 28911 Leganés, Spain; 3 Institute of Microbiology and Infection, College of Medical and Dental Sciences, University of Birmingham, Birmingham, B15 2TT UK; 4 Centre for Infectious Diseases and Microbiology, Westmead Institute for Medical Research, Sydney, NSW Australia; 5 Westmead Hospital,Western Sydney Local Health District, Sydney, NSW Australia; 6 School of Medicine, Sydney Medical School, University of Sydney, Sydney, NSW Australia

**Keywords:** Antimicrobial resistance, Bacterial infection, Epidemiology

## Abstract

Antibiotic resistance represents a growing medical concern where raw, clinical datasets are under-exploited as a means to track the scale of the problem. We therefore sought patterns of antibiotic resistance in the Antimicrobial Testing Leadership and Surveillance (ATLAS) database. ATLAS holds 6.5M minimal inhibitory concentrations (MICs) for 3,919 pathogen-antibiotic pairs isolated from 633k patients in 70 countries between 2004 and 2017. We show most pairs form coherent, although not stationary, timeseries whose frequencies of resistance are higher than other databases, although we identified no systematic bias towards including more resistant strains in ATLAS. We sought data anomalies whereby MICs could shift for methodological and not clinical or microbiological reasons and found artefacts in over 100 pathogen-antibiotic pairs. Using an information-optimal clustering methodology to classify pathogens into low and high antibiotic susceptibilities, we used ATLAS to predict changes in resistance. Dynamics of the latter exhibit complex patterns with MIC increases, and some decreases, whereby subpopulations’ MICs can diverge. We also identify pathogens at risk of developing clinical resistance in the near future.

## Abstract (web summary)

Pathogens are typically classified as ‘antibiotic-resistant’ for clinical purposes based on cut-off values of minimum inhibitory concentrations (MICs). In this study, the authors explore quantitative values of MICs using the global ‘ATLAS’ database of pathogen-antibiotic pairs, describe trends in resistance, and compare results to other antibiotic resistance surveillance data.

## Introduction

Antibiotic resistance is a peculiarly multiscale phenomenon. Small molecule antibiotics bind sites measurable on the nanoscale and yet those molecules, the pathogens they target and their resistance genes traverse our planet. As a result, understanding global changes in antibiotic resistance and its economic burden is particularly difficult. Governments have tried this, predicting future increases in resistance, disease morbidity and mortality^1,2^ but, possibly because of low fidelity antibiotic usage and resistance data, those long-term predictions have been criticised for relying on a narrow range of clinical conditions^3^.

How antibiotic usage mediates resistance is key. Self-evidently, reduced consumption should reduce resistance^2,4^ but this can be surprisingly difficult to establish. Some countries have successfully rationalised antibiotic use in farming^5^ but one problem when studying medical use may be timescales: theory predicts resistance rises faster than it decays following antibiotic withdrawal^6^, helping explain why a successful theory of antibiotic stewardship is so hard to develop^7^. The removal of an antibiotic may, or may not, lead to detectable reductions in resistance^8^ and so the body of evidence of how changing stewardship practice alters resistance has been described as ‘inconclusive’^9^.

More positively, resistance has reduced following pathogen-specific changes in antibiotic usage^10^, city-wide observations have also shown decreases in resistance^11^, as have hospital scale interventions, like hand-washing with alcohol^12^ or the isolation of patients carrying multidrug-resistant infections^13^. At the microbial scale, amplified resistance genes can be rapidly lost from microbial genomes following drug withdrawal^14^ although resistant species need not rescind completely from microbial communities post-treatment^15^.

In the clinic, longitudinal pathogen isolates from single patients can exhibit volatile resistance patterns, such as 500-fold weekly changes in antibiotic susceptibility that includes resistance changes to antibiotics not used for treatment^16^. However, such volatility may reflect a sampling problem of in vivo conditions whereby single clones are assayed that are not representative of the wider pathogen population.

Given the complexities, whether we can mitigate, or even reverse, resistance^17^ systematically is a difficult open problem. As a means to assess changes in resistance, hopefully reductions, high fidelity spatiotemporal data on the *status quo* is needed and Pfizer’s minimal inhibitory concentration (MIC) database, Antimicrobial Testing Leadership and Surveillance (ATLAS)^18^, published by Micron (https://micron-group.com), the Wellcome Trust and Open Data Institute, seeks this. To the best of our knowledge, ATLAS is the only open access MIC database where patient data are stored with clinical metadata (Table 1), making it a potentially important resource for assessing patterns in the dynamics of resistance.

**Table 1:** The 5 different antibiotic resistance databases used in this study: only ATLAS stores patient metadata which means that it can be compared with other databases in terms of their derived datatypes, but the converse is not possible.

database	datatype	patient metadata (pathogen, antibiotic and ...)
ATLAS	raw patient MICs	multiple MICs per patient/year/location/infection site
- TEST	a subset of ATLAS	multiple MICs per patient/year/location/infection site
		covering years 2004-2017
- INFORM	a subset of ATLAS	multiple MICs per patient/year/location/infection site
		covering years 2012-2017
- AWARE	a subset of ATLAS	multiple MICs per patient/year/location/infection site
	data not analysed herein	covering years 2008-2017
EUCAST	aggregated MIC histograms	no patient metadata; drug & pathogen are specified
ESPAUR	frequency of resistance	no patient metadata; drug & pathogen are specified
Resistance Map	frequency of resistance	no patient metadata; drug & pathogen are specified
ECDC	frequency of resistance	no patient metadata; drug & pathogen are specified

The acronyms used are: ATLAS (Antimicrobial Testing Leadership and Surveillance), EUCAST (the European Committee on Antimicrobial Susceptibility Testing), ECDC (European Centre for Disease Prevention and Control) and ESPAUR (English Surveillance Programme for Antimicrobial Utilisation and Resistance).

ATLAS can, in principle, be used to infer spatiotemporal changes in resistance for potentially hundreds of pathogen-antibiotic (PA) pairs and so we compare its MIC data both to fractions of resistance and to MIC histograms held in other databases, we then summarise the predictions ATLAS makes for MICs of important clinical pathogens. This analysis indicates MIC distributions exhibit hallmarks of phenotypic evolution whereby multi-modal MIC distributions have subpopulations that cluster around different MICs that shift each year. Some PA pairs exhibit mean MIC decreases even though high level resistance continues to increase. However, we show that some MIC dynamics in ATLAS may, in fact, be artefacts resulting from methodological choices made when the database was assembled.

## Results

Each ATLAS datum is a vector of antibiotic MICs assayed for one pathogen isolated from one patient in a known country, where each MIC is a dose that completely inhibits growth of the pathogen in an *i*n vitro drug-susceptibility assay. Pathogens are classified as resistant and an antibiotic is not recommended for treatment if the MIC lies above a published clinical breakpoint^19–21^ (i.e. susceptible isolates are those with MICs below the breakpoint, resistant isolates have MICs above the breakpoint). Thus MICs are a standardised, albeit variable, even laboratory- and assay-dependent^22,23^ resistance measure. Indeed, EUCAST (the European Committee on Antimicrobial Susceptibility Testing) acknowledges this when they determine so-called epidemiological cut-off values (ECOFFs^24,25^) that define breakpoints.

While paying attention to any noise, biases and anomalies that could result from discrepancies in MIC protocols, we seek signals in the ATLAS data collated during 2004–2017 that represent 6.5M MICs for pathogens from approximately 633k patients in 70 countries. While there are data for 284 pathogens, only those represented by more than 500 antibiotic susceptibility tests over 2 or more years were included in our initial sift that retained 43 pathogens and 827 pathogen-antibiotic pairs from all 3919 for later analysis (unless stated otherwise below). Of those 827, only 544 have published clinical breakpoints (defined by CLSI^19^) thus ATLAS has data for more PA pairs than are in current clinical use. So although all data derive from clinical assays, not all those assays were used to make clinical decisions.

### Between-database consistency: resistanceMap, ECDC, ESPAUR and EUCAST

ATLAS curators use different data sources, they acknowledge variability between those sources and address some inconsistencies in their documentation^18^. Descriptive statistics (Supplementary Fig. 1) indicate increases in data quantity through time, showing that US patients, *Staphylococcus aureus* and *Escherichia coli* infections dominate. ATLAS has a labelling bias whereby it can distinguish within-country MIC heterogeneities in the US because it contains state metadata, but it cannot do this for any other country.

ATLAS holds raw, anonymised patient MICs and metadata whereas some antibiotic programmes only report fractions of resistance longitudinally, like the English Surveillance Programme for Antimicrobial Utilisation and Resistance (ESPAUR) report in England^26^. There, the use of an essentially binary filter based on clinical breakpoints limits the analyses that can be performed.

**Fig. 1:**
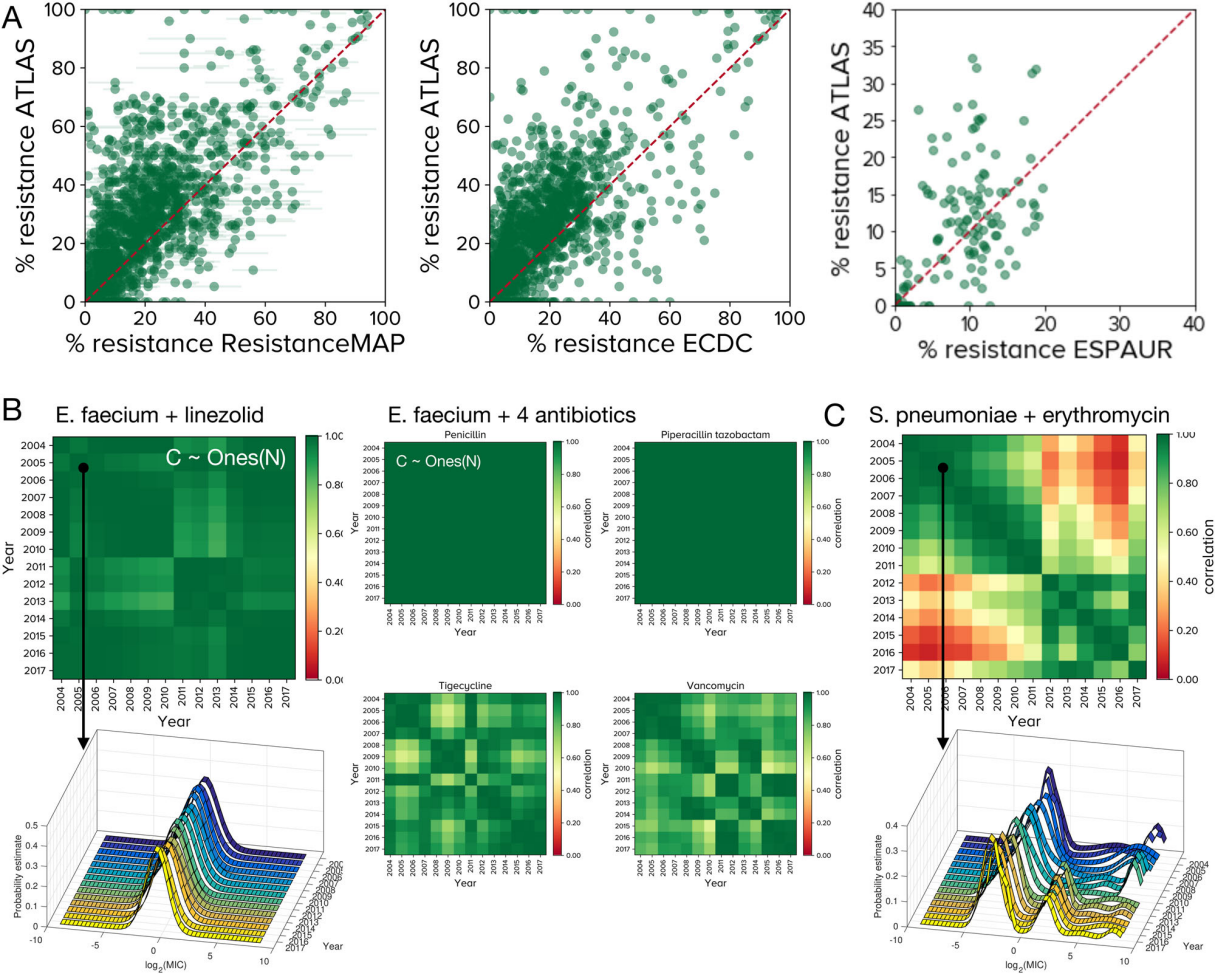
Between database comparisons with ATLAS. **A** Each point represents a PA (pathogen-antibiotic) pair in a given country, for a given year with frequency of resistance (*f*_*R*_ as a %age) on x and y axes. ATLAS tends to over-estimates *f*_*R*_ relative to ResistanceMap, ECDC and ESPAUR data: differences between *f*_*R*_ in ATLAS and other databases are positively skewed, however between-database differences are smaller for larger PA pair datasets (Supplementary Fig. 2 has statistics). **B** Between-year correlations for many ATLAS PA pairs form correlelograms, called `C' here, that are close to the (pure green) unity matrix of ones, Ones(N), for *N* years. 5 PA pairs for *Enterococcus faecium* are shown. (Supplementary Fig. 8C has some correlelogram statistics, Supplementary Fig. 9 shows many are close to Ones(N) but not all, see Fig. 3 and Supplementary Fig. 10). The left column shows the global MIC (minimal inhibitory concentration) distribution of *E. faecium* and linezolid is stable from year to year and its correlelogram is close to Ones(N). The middle panel shows 4 correlelograms with banded structures that occur when MIC distributions experience change. **C** This correlelogram of *Streptococcus pneumoniae* and erythromycin have a block structure because their MIC distributions correlate poorly between years: a high-MIC cluster diminishes and is replaced by a cluster with lower MIC in 2010–2011 (c.f. Fig. 4).

To test the consistency of ATLAS, we applied the CLSI susceptible-resistant classifier to ATLAS and compared that against recent ESPAUR^26^, European Centre for Disease Prevention and Control (ECDC)^27^ and ResistanceMap^28^ databases (also denoted RMap herein). This shows ATLAS has significantly many PA pairs with higher frequencies of resistance (skewed distribution tests; Fig. 1A, Supplementary Fig. 2) where between-database point differences can be as large as 60% for some PA pairs (even 100% for rare pairs, Fig. 1A, Supplementary Tables 2 and 3) Comparing ATLAS’s UK sub-dataset with ESPAUR’s 2013-2018 data, we find a moderate but statistically significant bias in ATLAS towards greater resistance (Fig. 1A, Supplementary Fig. 2). Moreover, within-country correlations between ATLAS and ECDC data can vary: they are high for France and Portugal but low for Denmark, Netherlands and others (Supplementary Fig. 3). Database differences are partially explained by larger PA datasets having statistically significantly smaller between-database discrepancies: where PA pair datasets have more than 500 points, all 3 databases agree with ATLAS to within a *circa* 20% frequency of resistance (Supplementary Fig. 2B quantifies the 3 correlations).

However, database comparisons like these can be affected by reporting methodologies. To understand how, consider that CLSI revises its breakpoints on occasion^29^ and because raw MICs do not change, resistance fractions reported by ATLAS one year need not be consistent with fractions reported following a revision which could affect comparisons between ATLAS, ECDC, ResistanceMap and ESPAUR. So while ATLAS reports raw MIC data labelled with CLSI breakpoints defined in 2018, reports for the 3 other databases are based on the breakpoints used each year of publication.

To assess the variation caused by breakpoint revisions, we re-analysed ATLAS based on its fractions of resistance, mimicking the reporting methods of ECDC, ResistanceMap and ESPAUR but this time, we used revised CLSI breakpoints^29^ (Supplementary Table 1) and re-analysed PA pairs where revisions occurred. This shows (Supplementary §8) that breakpoint revisions can affect between-database comparisons by an approximately 20% frequency of resistance and only where PA pairs have large enough sample sizes, above ~ 100 patients, is this percentage lower (Supplementary Fig. 14).

**Fig. 2:**
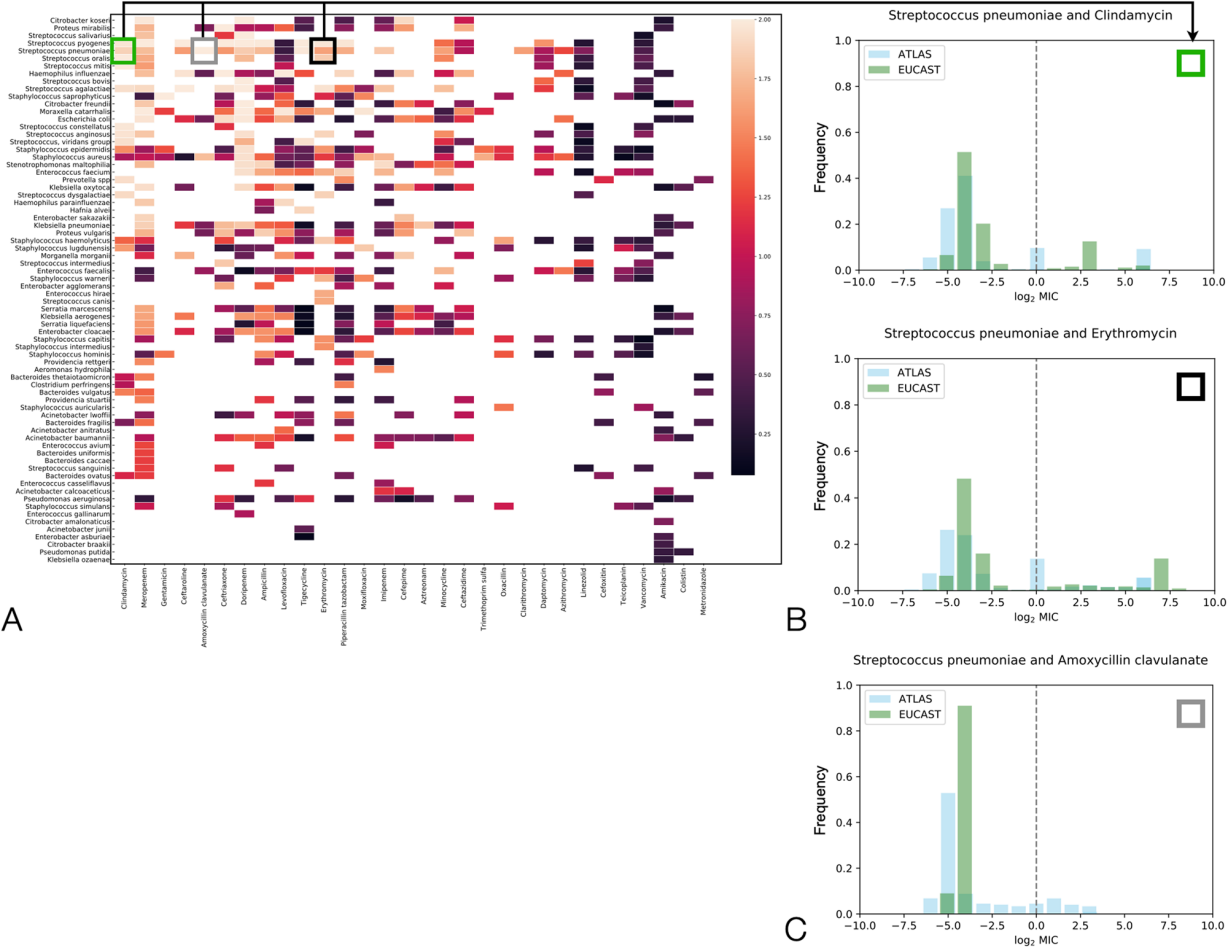
Comparing ATLAS with MIC histograms from EUCAST: from the worst to best correspondences. **A** If an MIC (minimal inhibitory concentration) histogram from EUCAST is the vector ***x*** and an analogous histogram is determined from ATLAS (by aggregating across all years) and denoted ***y***, this heatmap shows the Euclidean distance ∥***x*** − ***y***∥_2_ for each pathogen-antibiotic (PA) pair in both databases: squares indicate distances on a colour scale, black denotes close agreement and pink denotes poor agreement, white shows no comparison can be made. Axis labels are ordered whereby the biggest ATLAS-EUCAST disagreements are generally found leftmost and uppermost. **B** Two MIC histograms are shown whereby the open black and green squares superimposed on A highlight *S. pneumoniae* and clindamycin and erythromycin as having among the biggest disagreements between ATLAS and EUCAST. **C** The case of *S. pneumoniae* and amoxycillin clavulanate is one of 10 antibiotic PA pairs for which ATLAS has resistant subpopulations according to CLSI breakpoints, but EUCAST does not (also Supplementary Fig. 25).

We compared ATLAS with MIC histograms published by EUCAST, noting the latter do not publish time or location metadata with each MIC. ATLAS data must, therefore, be pre-processed to remove all metadata before this comparison can be made (“Methods”). The degree of ATLAS-EUCAST correlation differs between PA pairs (Fig. 2A) which is expected because EUCAST have curated PA pair data for a longer period of time and typically have larger datasets. However, despite this, we found 10 PA pairs in ATLAS with MICs residing above the corresponding CLSI breakpoint whereas the analogous EUCAST entries lie below those breakpoints (Fig. 2C and Supplementary Fig. 25).

### Potential for bias in ATLAS

Given the tendency to report higher frequencies of resistance, we asked whether biases might be encoded in the ATLAS methodology. Statistical modelling shows a systematic bias towards greater resistance would not be consistent with the above observation of greater between-database similarities at larger PA database sizes (Supplementary §3). Thus, ATLAS’s differences from other public databases appear more subtle than there simply being a systematic bias towards sampling more resistant strains.

Plausible biasing mechanisms could arise if (1) data on resistance to particular drugs is specifically targeted, then there could be an unintentional human operative or programme-encoded bias towards submitting resistant isolates at all contributing centres. Or, (2) if small numbers of contributing centres sampled large numbers of strains, this could result in some geographically-localised strains (e.g. US-based) being over-represented. We can partially address (2) by stratifying our analyses by geography and we will do this below when comparing MIC dynamics of the most resistant strains.

To address (1), we note ATLAS comprises data from so-called TEST and INFORM surveillance programmes developed by Pfizer and AstraZeneca, respectively, which were designed to quantify tigecycline and ceftazidime-avibactam (*a.k.a*. CAZ-AVI: the *β*-lactamase inhibitor avibactam inhibits class C enzymes, restoring ceftazidime susceptibility^30^) MICs and this necessitates careful consideration. Contributing centres to these 2 programmes could, consciously or unconsciously, choose to test and submit more resistant isolates because these companies were interested in the efficacy of their drugs against problematic clones. However, documentation authored by the Wellcome Trust and the Open Data Institute, with advice from bodies such as Public Health England (now Health Security Agency UK), claims data in ATLAS are of high quality^18,31^ (Supplementary §1-2).

Now, CAZ-AVI is often tested against strains resistant to frontline treatments so the likelihood of reporting as resistant by INFORM may be biased due to cross resistance because it uses strains that already proved themselves resistant against other treatments. Thus, when CAZ-AVI MICs are contrasted with earlier surveys to determine its wider efficacy, this could appear in ATLAS as a resistance shift that merely reflects INFORM’s design and this issue may affect the newest antibiotics, like CAZ-AVI, most. On that basis, is ATLAS data quality impaired if it over-represents resistant isolates that were thereafter submitted for testing against the newest antibiotics?

According to its publishers, this is not how ATLAS was designed and this is consistent with the lack of modelling support above for a systematic bias hypothesis. Instead, hospitals that agreed to contribute to surveillance programmes chose isolates as part of routine clinical practice who then tested clones against antibiotics suggested by Pfizer or AstraZeneca. Database curators^18,31^ claim there is no bias in isolate selection, stating that submitted clones were already due to be tested (Supplementary §2) supports this with quotations. If this is true, criticising ATLAS because isolate bias is the result of targeting CAZ-AVI-resistant strains would not appear justified, although CAZ-AVI testing does have a large representation in ATLAS (Supplementary Fig. 6), exactly as the programme set out to achieve.

We were then concerned ATLAS could bias towards low MICs following years in which pathogen sampling methods changed. For instance, could heightened awareness of resistance have lead to increased clinical susceptibility testing that could, in turn, increase the reporting of low MICs? Or could improvements in molecular identification methodologies^32,33^ have an analogous effect?

To address this, we sought PA pairs with a changepoint in the size of their ATLAS dataset: of all 3,919 PA pairs, 1,718 have a data size changepoint of which 203 occur in 2012, while 657 occur in 2013 when both TEST and INFORM increased in size (ATLAS doubled in size in 2012 following the inclusion of INFORM and it increased again in 2013 by around 50%, Supplementary Fig. 1C).

We then sought PA pairs that initially exhibited an increasing MIC until a significant increase in data occurred at the same time as a significant decrease in MIC (a positive-then-negative MIC change). These increases and decreases were tested using statistically significant linear regressions (using *p* < 0.05, noting smaller *p* values would detect fewer PA pairs) and the number of PA pairs exhibiting this MIC change in its data size changepoint year is 11 (Supplementary Fig. 7A), suggesting that growing or merging TEST and INFORM impacted the inference of MIC dynamics for at least 11/3919 PA pairs. We then asked whether the data size changepoint occurred in the same year as a negative-then-positive MIC change, finding a further 25/3919 PA pairs exhibit this property (Supplementary Fig. 7B). This provides 36 putative PA pairs for which changes in data availability may have a methodological significance that we must account for in any analysis of the clinical predictions ATLAS makes. Interestingly, 7/36 of these involve tigecycline, the target drug of TEST (Supplementary Fig. 7).

### Within-database consistency: ATLAS year by year

ATLAS comprises patient samples that are not longitudinal on a per patient basis. It is therefore plausible that MICs of a given PA pair exhibit no between-patient correlation or between-year coherency and, instead, resemble a noise process. As MICs are resolved only by year, each PA pair is not associated to an MIC timeseries but, rather, to a set of MICs of different cardinalities for each year.

**Fig. 3:**
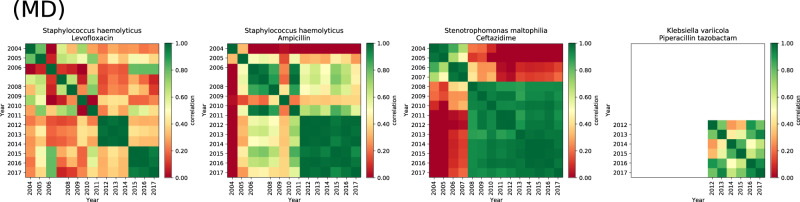
Four of the “worst” pathogen-antibiotic (PA) pair year-year correlations ranked by *τ* (as defined in Supplementary §5): these satisfy *τ* < 1/4. PA pairs typically have low values of *τ* because their correlelograms exhibit block structures consistent with high year-year correlations between MIC distributions that incorporate sudden changes. The particular MIC correlations shown here are for PA pairs that satisfy *τ* < 1/4 that are also classified as both D and M, as described in the text. An M label refers to a PA pair where sampling was very low in one particular year and pairs with high sampling variability across years are labelled with a V. Not shown here are other PA pairs with low sampling size across years (labelled as N) and ones where the discrepancy between TEST and INFORM MICs is high (labelled as D). Pairs that do not fall under any of these categories are labelled with a U and Supplementary Fig. 10 shows corelelograms for all the remaining PA pairs that satisfy *τ* < 1/4.

We therefore asked whether MIC distributions for PA pairs from year *y*_*i*_ would correlate with year *y*_*j*_. We call the symmetric matrix of all year-year correlations, *C*_*ij*_, “correlelograms”, examples of which are shown for *Enterococcus faecium*, linezolid and 4 other antibiotics (Fig. 1B). Testing the distributions of singular values of *C*_*ij*_ shows ATLAS has significantly greater correlations between-years for PA pairs than expected for noise processes (*p* < 10^−16^, Supplementary Fig. 8) and PA pairs with the lowest between-year correlations typically have data for the fewest years (Fig. 3 and Supplementary Fig. 10). However, many PA pairs have coherent but slowly changing datasets for several years’ duration (Fig. 1B, Supplementary Fig. 9).

We did not remove PA pairs with the lowest between-year correlations (Fig. 3 and Supplementary Fig. 10) from analysis at this point because it is plausible that absences of correlation have a microbiological basis in those cases where the data are recorded for sufficiently many years. For instance, low correlations could result if a highly resistant strain swept a pathogen population very quickly in some part of the globe in year *Y*, creating low MIC correlations in the years before *Y* and those after *Y*. We discuss this idea in more detail below.

### An example of TEST-INFORM inconsistency following their 2012 merge

Some MIC distributions change abruptly so we investigated why. *Streptococcus pneumoniae* and erythromycin have a modular correlelogram structure (Fig. 1C) reflecting the apparent loss of a high-MIC subpopulation in 2011, call it R-strep. On examining this PA pair, we found international definitions of susceptibility for *S. pneumoniae* were revised in 2008 resulting in more strains appearing susceptible^34^. While this could affect the sampling behaviour of clinicians which would impact R-strep data, it does not alter a reported MIC following a susceptibility test so it is unclear whether this is sufficient to explain the reduction in the R-strep subpopulation.

**Fig. 4:**
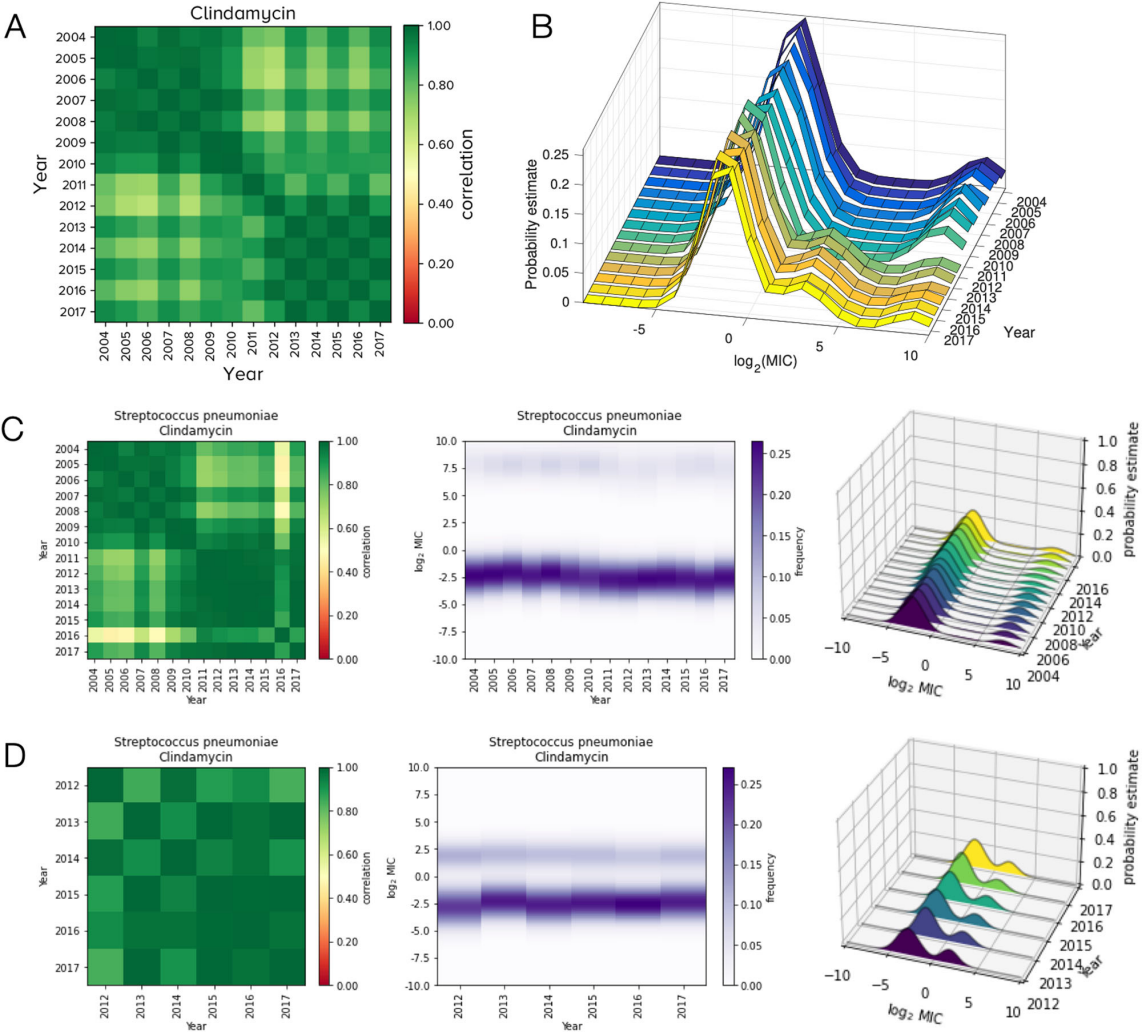
Evidence *S. pneumoniae* and clindamycin MIC dynamics are the result of database curation methodologies. Show an analogous structural shift to Fig. 1B, C for *S. pneumoniae* and erythromycin: the correlelogram (in **A**) and MIC dynamics (in **B**) shift towards lower MICs around 2011. The result of separating ATLAS into its (**C**)) TEST and (**D**)) INFORM components are then shown. These indicate the loss of the most-resistant cluster from B around 2011 is due to TEST and INFORM both having bimodal MIC distributions with different high-MIC clusters (see C(right) and D(right)). When both the latter are amalgamated to form ATLAS, noting (**D**) has data only after 2012, this appears (in (**B**)) to change the structure and dynamics of the MIC distributions when, in fact, these are artefacts reflecting the merging of 2 datasets.

Furthermore, the introduction of conjugate vaccines (7-valent in 2000, 13-valent in 2010) reduced the incidence of antibiotic resistant invasive clones and, in particular, clindamycin resistance fell following the 2010 vaccine in developed countries^35,36^. As *erm* genes confer resistance to both erythromycin and clindamycin^37,38^. Fig. 1C may reflect a correlated change in high-level resistance to both antibiotics and, supporting this, we observe a similar 2011 shift for *S. pneumoniae* and clindamycin (Fig. 4A, B).

However, these clinical factors are probably irrelevant to ATLAS. Deeper investigation reveals a problem to the extent that *Streptococcus pneumoniae*, erythromycin and clindamycin illustrate how the ATLAS methodology can create artefactual MIC distributions. Figures 1C and 4B show a sudden decrease in frequency of a highly-resistant subpopulation in the MIC distribution of these two PA pairs but Fig. 4C, D reveal what actually happened: both TEST and INFORM components of ATLAS have distinct subpopulations within respective bimodal MIC distributions that consist of susceptible and resistant strains where the MICs of these subpopulations are different. So, when TEST and INFORM were combined in 2012, data from Fig. 4C and D merged to form Fig. 4B. As the latter has the apparent shift in resistance but Fig. 4C, D do not, this shift must be an artefact of merging databases. Moreover, the long-term stable presence of different MIC clusters in Fig. 4C, D indicates that TEST and INFORM must differ in their methodologies: either they are using test laboratories that report very different MIC values for the same strains (unlikely) or they are sampling phenotypically distinct *S. pneumoniae* subpopulations.

To identify PA pairs with curation problems systematically, we sought TEST-INFORM discrepancies using a metric-like statistic, Δ, that quantifies (Supplementary §6) differentially susceptible subpopulations in TEST and INFORM based on an ‘S-R’ clustering methodology that is described in detail below. Ranking PA pairs according to Δ indicates *S. pneumoniae*, erythromycin and clindamycin have the biggest TEST-INFORM discrepancies followed by *Haemophilus influenzae* and ceftazidime, *Staphylococcus aureus* and ampicillin, *Streptococcus pyogenes* and meropenem, and then *Klebsiella oxytoca* and ceftazidime (Supplementary Fig. 11). These 4 pairs have MIC distributions with high year-year correlations within each database and yet between-database correlations are low, with different modal MIC dosages, again as if TEST and INFORM sampled from different populations (Supplementary Fig. 12).

We hypothesised EUCAST’s MIC histograms might also help identify curation problems in ATLAS. After removing all temporal metadata so ATLAS can be compared with EUCAST (Methods), we found *S. pneumoniae*, erythromycin and clindamycin have among the greatest ATLAS-EUCAST discrepancies in that comparison too (Fig. 2A). Despite this, both ATLAS and EUCAST do share some features for these PA pairs in the sense that both have trimodal MIC histograms where the modes lie at similar MIC values (Fig. 2B). Thus, following the removal of metadata, aggregating TEST and INFORM yields a dataset that is similar to EUCAST, even for PA pairs where TEST and INFORM are dissimilar.

Despite these problems, we assume from here on that methodological problems are sufficiently rare that some clinical signals can still be found within ATLAS but, as the above cases highlight, we must be aware of the potential for finding artefacts as we proceed.

### Directional changes in resistance: the cluster of greatest MIC (*R*)

**Fig. 5:**
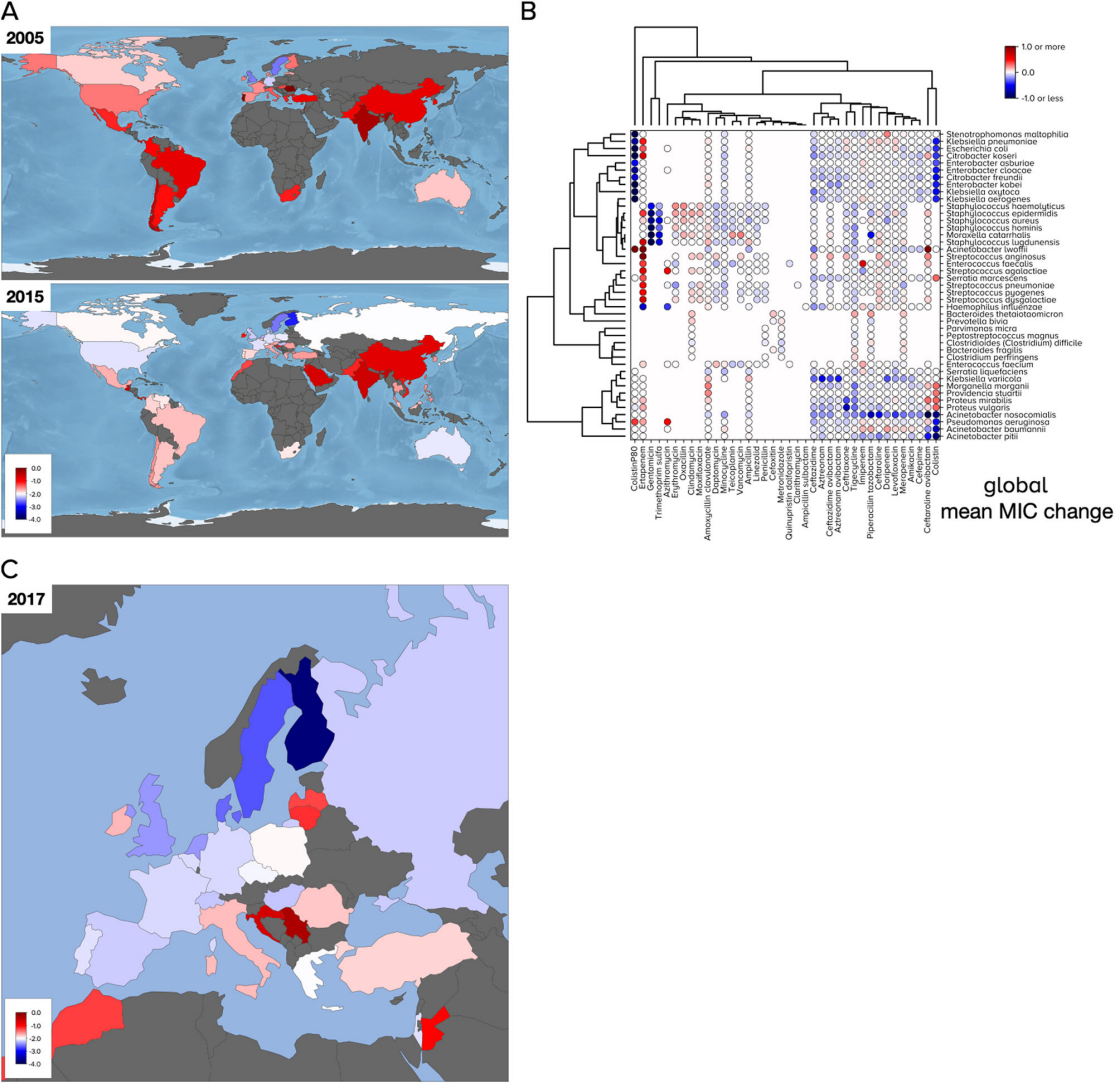
Coarse changes in mean MIC (minimal inhibitory concentration) for all pathogen-antibiotic (PA) pairs: some decreases with increases in Asia. **A** The distribution of mean MICs for PA pairs aggregated globally: a potential reduction is apparent from 2005 to 2015 in some countries with increases in India and China. **B** The slope of a linear regression attempts to predict mean MIC changes globally for all PA pairs and is shown as a dot. A blue-to-red colour scale indicates normalised ${\log }_{2}$ MIC change per year: red is +ve, blue is -ve, light grey is no change, grey countries in A and C have insufficient data, as do white regions in B. Dots are clustered into PA pairs of similar change, indicative of a global motif of predicted increases and decreases; the same analysis using European-only data is Supplementary Fig. 15A. **C** European MICs aggregated across all PA pairs in 2017 shown by country indicate possible differences between eastern and western Europe. Colourbars in A and C show normalised ${\log }_{2}$ MIC units.

The finding that ATLAS is not an ensemble of uncorrelated MICs though nor is it a set of stationary MIC distributions is consistent with pathogens undergoing evolutionary change with the MIC as a phenotype. A standard quantitative genetics approach to elucidating MIC dynamics would be to linearly regress MIC against time so we did this for every PA pair, both for global data (Fig. 5A, B) and for Europe (Fig. 5C, Supplementary Fig. 15A). The predicted changes in MIC are curious: apart from South-East Asia and Central America, particularly India, China and also Ireland, Serbia and Croatia (Fig. 5C), regressions predict more PA pairs have global MIC reductions than increases from 2005 to 2015 (Fig. 5B, *p* < 0.001, skewness test using scipy.stats.skewtest). This observation is analogous if just US or European data are used instead of global data (Supplementary Fig. 15A). Given the wide reporting of increased resistance and the fact that ATLAS typically exhibits greater frequencies of resistance than other datasets, this skew seems anomalous. One explanation could be that MIC distributions have highly-resistant tails that exhibit different dynamical behaviour to their means, so we tested this, as follows.

A clustering methodology was employed to replace the clinical, binary categorisation of pathogens into susceptible (S) and resistant (R) strains by, instead, seeking clustered subpopulations in MIC distributions with the highest and, thereafter, lower MICs. For this we noted that MIC data is said to be log-normally distributed^25,§3.1.4]^ and so we are justified in the following procedure. By modelling log-transformed MIC distributions as Gaussian mixtures, namely a superposition of *k* different normally distributed clusters, we determined an information-optimal value of *k* for each PA pair for each year (Supplementary Figs. 16, 17). The most-resistant sub-population, R, is then defined to be the cluster with greatest mean MIC and S is the complement of the R cluster. The boundary between S and R is that MIC value which has an equal likelihood of being in either sub-population. According to this definition R need not represent clinically resistant strains, rather it is the cluster of strains with the lowest antibiotic susceptibility for the PA pair under study, a property that makes it a useful filter for conducting a “worst case analysis” over a defined geographical region or time period. For these analyses, the mean MIC the R cluster will be called the R-MIC, the S-MIC is defined analogously.

Although we found no evidence of systematic resistance bias in ATLAS, we remark that, even if we did, the R-MIC is useful because it has a robustness property to positive resistance bias that the S-MIC and the mean MIC do not share: unless the bias is extreme and only leads to the sampling of data in the very high-resistance tail of the MIC distribution, R-MIC values determined from resistance-biased sampling are less affected than samples that contribute to the S-MIC (Supplementary §3, Supplementary Fig. 5). Indeed, the latter are more likely to be excluded from analysis by a methodology biased towards sampling data with high MICs. So even if ATLAS has methodological biases towards sampling more resistant strains, this should affect R-MIC dynamics less than it would S-MIC dynamics.

**Fig. 6:**
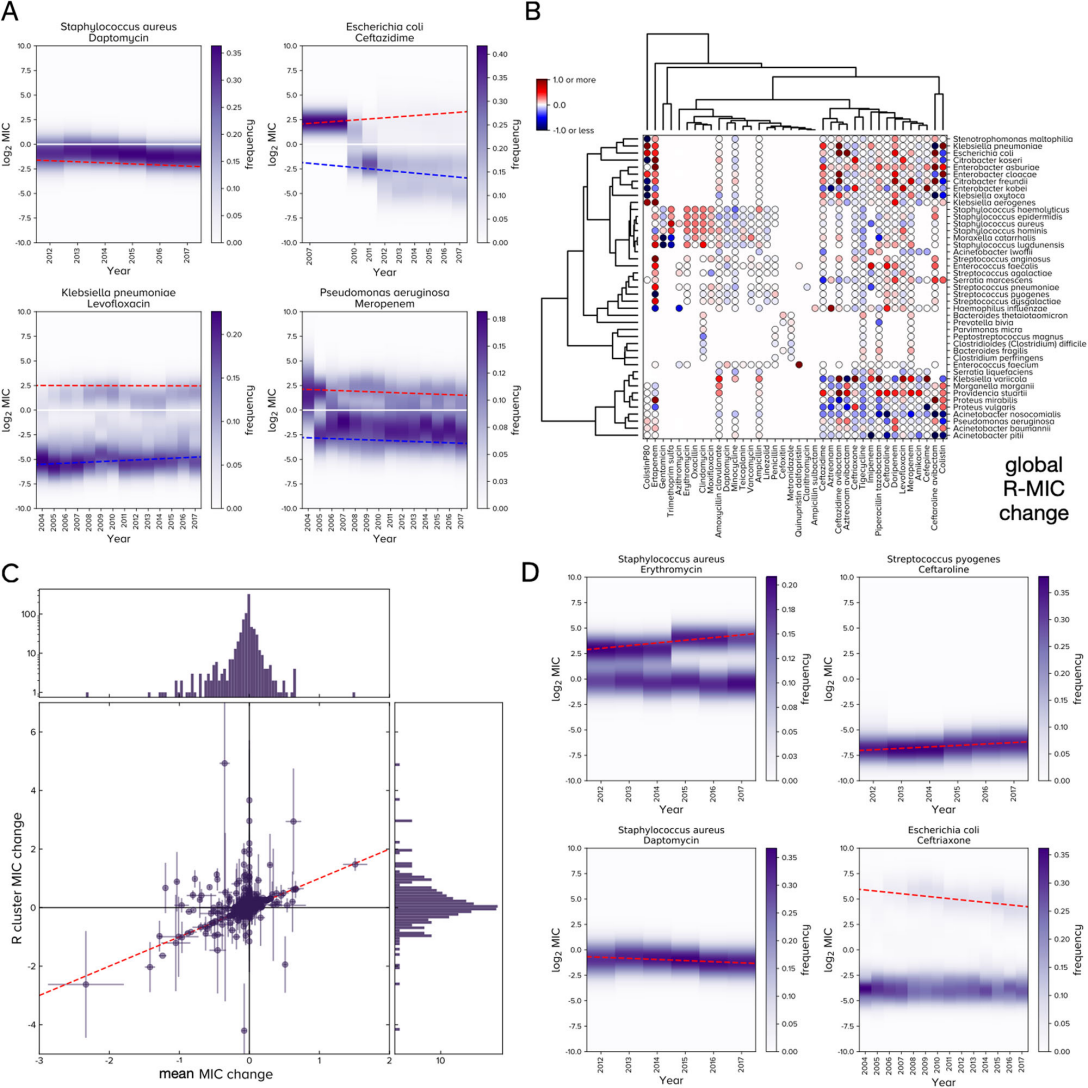
The R-MIC and its global dynamics for pathogen-antibiotic (PA) pairs are different from mean MIC dynamics shown in Fig. 5. **A** Example dynamics of the S-MIC (blue dash) and R-MIC (red dash) are shown for 4 PA pairs: these have 1, 2 or 3 clusters in their MIC distributions and linear regressions predict the changes of the R-MIC (red) and S-MIC (blue). **B** Predicted global R-MIC changes per year for each PA pair (${\log }_{2}$ MIC change per year) are shown as a clustergram (the analogous plot with European data is similar, Supplementary Fig. 15B). **C** Changes in R-MIC (y-axis) and mean MIC (x-axis) are not well correlated (one dot per PA pair, *ρ* = 0.33, red dash shows `y=x' to highlight equal changes, crosshairs indicate s.d. from synthetic ATLAS replication, *n* = 50) because many PA pairs have R-MIC decreases and mean MIC increases and vice versa (c.f. Figs. 5B and 6B or Supplementary Figs. 15A and B). **D** These 4 PA pairs illustrate 4 MIC dynamics whereby R clusters have either sub- or super-breakpoint MICs which either increase or decrease (making 4 possible cases).

To determine S and R clusters robustly, we repeated the Gaussian clustering on 50 synthetic replicates of ATLAS, each with small-variance noise added to every MIC (“Methods”). We then determined MIC changes, a.k.a. derivatives, for each replicate by applying longitudinal regression to S and R separately to estimate time derivatives *d*S/*dt* and *d*R/*dt* (Fig. 6A). In summary, this procedure found *d*R/*dt* statistics are increasing and not decreasing for many PA pairs (Fig. 6B). The above anomaly arises, therefore, because many PA pairs have mean MIC or S-MIC decreases and yet their R-MIC is increasing (Fig. 6C and Supplementary Fig. 18).

Phase planes formed from (*d*S/*dt*, *d*R/*dt*) summarise patterns of longitudinal MIC change. Consistent with longitudinal regressions performed above, S-MICs and R-MICs do not always increase in time: cases of divergent MICs are present in ATLAS whereby the S-MIC is static or decreasing while the R-MIC increases (Fig. 6A). Similarly, there are cases where the S-MIC and R-MIC tend towards a common value, the most clinically optimistic case whereby the S-MIC is stable, or decreasing, while the R-MIC is decreasing can also be found. Divergent MICs are the most common of these cases where R-MIC increases are typically greater than the respective change in the S-MIC or mean MIC; all these behaviours are found in data (Fig. 6 and Supplementary Fig. 18).

### Clinical observations

**Fig. 7:**
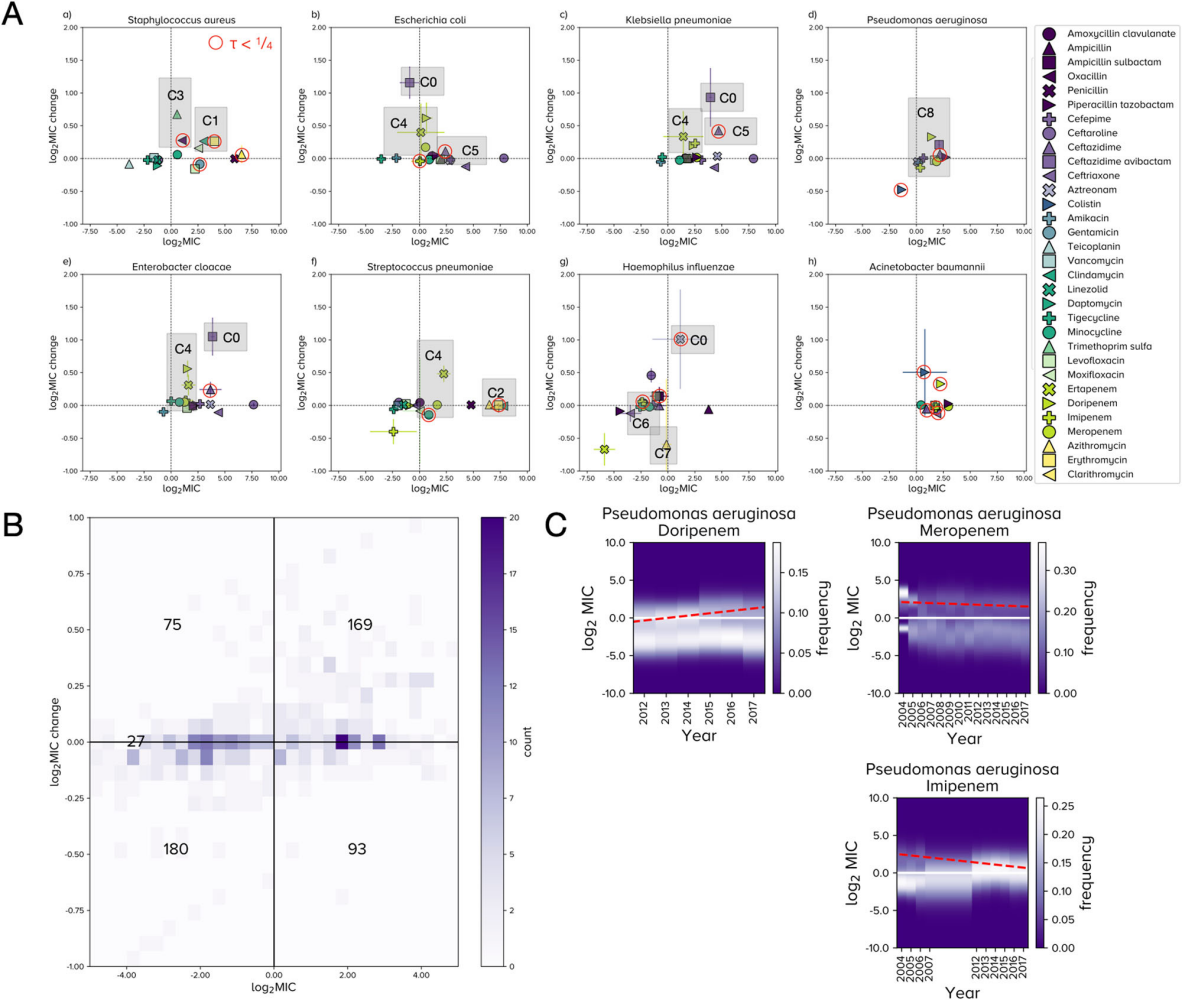
Eight clinically important pathogens and their R-MIC dynamics. **A** Each panel shows the (*R*, *dR*/*dt*) phase plane (x-axis: 2017 data, the most recent value in ATLAS; the normalised ${\log }_{2}({\mathsf{MIC}})=0$ axis represents the clinical breakpoint, see “Methods”). Data are means and crosshairs are s.d. under synthetic ATLAS replication with *n* = 50 (“Methods”). *Acinetobacter baumannii* exhibits clinical resistance to all ATLAS antibiotics and the other phase planes visibly skew towards resistance. Labels C0-C8 refer to clinical observations made in the main text. Data with low year-year correlations satisfying *τ* < 1/4 (Fig. 3 and Supplementary Fig. 10) are circled red. **B** A 2d heat map counts which regions contain 544 pathogen-antibiotic pairs in the (*R*, *dR*/*dt*) phase plane. **C** Example changes in R-MIC using global data for *Pseudomonas aeruginosa* and 3 carbapenems: doripenem MICs are increasing from sub- to super-breakpoint whereas meropenem appears stable. Linear regression of R (red dashes) indicate the R-MIC of imipenem is slowly decreasing whereas, in fact, inspection of the underlying data shows this R-cluster is merging with an S sub-population as both achieve super-breakpoint dosages and the population transitions from a bimodal to unimodal MIC distribution.

We investigated (R, *d*R/*dt*) phase planes which indicate where R was in 2017 and estimate where it could be heading (Fig. 7A, B). For completeness, phase planes of the entire ATLAS database are summarised as a heatmap (Fig. 7B) and phase planes of 8 important pathogens (Fig. 7A) visually skew towards R-MIC increases. These phase planes exhibit clinically relevant trends consistent with previous reports but some observations were less expected, as follows. In the following, references to ‘label CX’ appear in Fig. 7A.

*Acinetobacter baumannii* resists all antibiotic classes with scant evidence of R-MIC reductions (Fig. 7A). Reports of colistin resistance in *A. baumannii*^39^ are consistent with an R cluster first detected in ATLAS in 2015 (Supplementary Fig. 16B). The most rapid R-MIC increases in ATLAS are for ceftazidime avibactam (CAZ-AVI; label C0) which has a novel R-cluster first detected for *Klebsiella pneumoniae* in 2014 that has an increasing R-MIC (Supplementary Fig. 16A). Enterobacteriaceae E. coli and *E. cloacae* have a large change in R-MIC for CAZ-AVI (label C0) because an R cluster recently appeared with R-MIC transitioning from sub- to super-breakpoint (Supplementary Fig. 19). A positive change in R-MIC is predicted for *H. influenzae* and aztreonam (label C0) but it is not significant under synthetic ATLAS replication.

Erythromycin and clindamycin exhibit analogous behaviour against *S. aureus* whereby resistance is increasing at similar rates (label C1), consistent with the function of *erm* genes^37,38^. Conversely, and consistent with other reports of a plateau^40^, erythromycin resistance in *S. pneumoniae* is high but not increasing (label C2) and its “corrected” MIC distributions (formed by separating INFORM and TEST into 2 databases) support this observation (Fig. 4C, D).

*S. aureus* resistance to trimethoprim sulfamethoxazole (TMP-SMX) and oxacillin are increasing (label C3). Oxacillin has substituted methicillin and TMP-SMX is used against methicillin- (thus oxacillin-) resistant *S. aureus*^41^ and this trend suggests TMP-SMX is failing to mitigate resistance in *S. aureus*, as noted elsewhere^42^. Secondary prescribing behaviour may be important as methicillin-resistant *S. aureus* clones emerged from antibiotic-susceptible community lineages and TMP-SMX became an important oral agent for community-acquired non-multiresistant MRSA (CA-MRSA)^42^ which may have driven TMP-SMX resistance. The R-MIC of TMP-SMX is significantly above the clinical breakpoint with a positive time derivative (label C3), indicating treatment of MRSA with TMP-SMX may be at risk.

In terms of beta lactams, ATLAS exhibits the following. Carbapenem MICs for *K. pneumoniae* have been reported as bimodally distributed where high MICs reflect outer membrane protein (OmpK36) defects^43–45^, this is consistent with distinct S and R clusters (Supplementary Fig. 17). Increasing carbapenem MICs^46^ are found in ATLAS (label C4; enterobacteriaceae E. coli, *K. pneumoniae*, *E. cloacae* and the Gram-positive *S. pneumoniae*) as are increasing ertapenem MICs against *E. cloacae*^47^ (label C4). Ceftazidime is following known trends in uropathogenic E. coli^48^ (label C5) that derive from mobile CTX-M genes^49^ that may contribute to the R-MIC increases of ertapenem in E. coli and *K. pneumoniae*^50^ (label C4), possibly reflecting changes in OmpK36; the rate of ceftazidime R-MIC increases for *K. pneumoniae* are greater than for E. coli (label C5).

In contrast with smaller studies^51,52^ where differences in carbapenem resistance were not detected, the R-MIC of doripenem in ATLAS is increasing more quickly than other carbapenems against *P. aeruginosa* (label C8). This may be due to changes in efflux-mediated cross-resistance between carbapenems^53^. More speculatively, it might even have resulted from a change in manufacturing or usage base as carbapenem patents expired in the decade after 2010. Seeking to better understand this, we found doripenem has the fastest increasing carbapenem R-MIC in almost all countries (Supplementary Fig. 20) thus between-country differences do not explain doripenem’s R-MIC rise in ATLAS. S- and R-MICs of doripenem against *P. aeruginosa* are converging towards those of meropenem (Fig. 7C) and we speculate doripenem’s increase might result from recommendations shifting to mitigate resistance in other carbapenems^54–56^. Consistent with this, a 2019 survey of 20 US hospitals shows while doripenem has more variable usage data, it was the most used carbapenem measured in days of treatment per patient day whose defined daily dosage was as high as half of the most-used carbapenem^57^, Tables 1 and 3.

*H. influenzae* is susceptible to minocycline with no significant increase in R-MIC (label C6) so we predict this drug can be used against beta lactam-resistant *H. influenzae*. We find no evidence of azithromycin resistance in *H. influenzae* and, interestingly, resistance may be marginally decreasing (label C7). However, the number of clinical cases supporting this observation is fewer than 100 per year in a narrow year range (2015 to 2017) and this prediction is not statistically significant under synthetic ATLAS replication.

To highlight the potential for unreliability in (R, *d*R/*dt*) phase planes, correlelograms of PA pairs in labels C1-8 with year-to-year correlations that satisfy *τ* < 1/4, where *τ* is a measure of year-year correlation (Supplementary §5) are indicated (19 in Supplementary Fig. 23 in addition to *S. pneumoniae* and erythromycin), making 20 PA pairs with *τ* < 1/4 in Fig. 7A.

We then investigated all 128 PA pairs satisfying *τ* < 1/4, classifying them using the following scheme: a letter N denotes 49/128 pairs that have low mean sample sizes, M denotes 66/128 pairs with a low minimal sample size observed somewhere within their time period, V denotes 34/128 pairs with highly variable sample sizes, D denotes 25/128 pairs with high TEST-INFORM discrepancies which leaves 33/128 pairs labelled with a U (for “unclear”, Fig. 3 and Supplementary Fig. 10, Supplementary §7). Some pairs suffer from multiple problems simultaneously within this classification.

Why would these 33 PA pairs have low *τ*? These cases have various MIC changes, both increasing and decreasing, above and below the clinical breakpoint (Supplementary Fig. 13). Technical problems with colistin and colistinP80 testing might explain poor year-year correlation in some cases (Supplementary Fig. 13). Some colistin PA pairs exhibit a trend whereby a slowly moving S cluster centred around −2.5 in breakpoint-normalised ${\log }_{2}$ units suddenly jumps to around −5.0 (deduced from INFORM data only). Importantly, the material of the microtitre plate affects colistin susceptibility testing^58^ and P80, a surfactant, was used to improve in vitro testing by preventing colistin from adhering to surfaces, a practice discontinued in 2014^59^. INFORM curators claim they followed CLSI methodologies^60^ and, consistent with this, there is no INFORM data for colistinP80 after 2014 (although colistin data is available after 2014: Supplementary Fig. 13, see *C. freundii* and *K. oxytoca*). Of the 33 U-classified PA pairs, 11 involve colistin, 9 have data for just 4 years, or less and 12 change markedly between 2014 and 2015 (Fig. 3 and Supplementary Fig. 10) and, as these occur in the same year, this degree of coincidence could indicate anomalies in data curation processes.

The only remaining PA pair of the 128 identified not suffering one of these issues is *Haemophilus influenzae* and ampicillin sulbactam which has low year-year correlations because it exhibits quickly changing MICs close to, but mainly below, the clinical breakpoint. Moreover, MIC variance increases through time, first with a decreasing and then an increasing mean MIC (Supplementary Fig. 13) whereby the frequency of resistance in ATLAS is broadly consistent with other recent reports^61^.

Finally, to ascertain whether regional differences might affect analyses of ATLAS we did the following. Stratifying data according to their origins in either US, Europe or Rest of the World, we constructed 3 sub-databases of nearly equal size and asked whether R-MIC dynamics differed between them (Supplementary Fig. 21). Accordingly, there is good agreement between regions provided the cardinality of each PA pair dataset is sufficiently large (within 10% for all but 9 PA pairs; Supplementary Fig. 21D). However, the largest differences between regions occur for colistin, ertapenem and CAZ-AVI (Supplementary Fig. 21C). This is noteworthy because CAZ-AVI was the target drug of INFORM and one should therefore expect high between-region consistency for this drug, given its high level of representation in ATLAS (Supplementary Fig. 6).

## Discussion

We compared ATLAS against ResistanceMap, ECDC and ESPAUR, which only provide frequencies of resistance, not raw MICs, and we found ATLAS generally biases towards higher frequencies of resistance. We also compared ATLAS with EUCAST MICs curated from a wide variety of sources, finding different degrees of consistency between them for different PA pairs. EUCAST does not employ the R-MIC statistic and although one could apply it to their histograms, we have not done so because one cannot then infer rates of R-MIC change due to the absence of metadata.

Our computational approach, which, for openness, can be downloaded freely (Methods) clusters MICs of PA pairs and assigns MICs to an S or R class according to whichever has the maximum likelihood under the assumption of lognormally distributed clusters. This provides one advantage relative to the standard clinical classification based on published breakpoints: it can track the dynamics of the least antibiotic susceptible cluster, R, from the time it first emerges and not the time it first crosses the breakpoint. By contrast, databases that only publish frequencies of resistance are compelled to report the value zero until the time that crossing first occurs. As a result, the R-MIC would prove useful for tracking changes in susceptibility for the newest antibiotics that are yet to exhibit clinical resistance.

Applying R-MIC to data aggregated across all countries in ATLAS or across multi-country regions, like the EU, provides a worst case analysis of MICs for those regions. However, ATLAS is not well-suited to studying differences within countries and yet these exist, whether because of resistance heterogeneities, differences in farming policy, clinical stewardship practice and prescribing culture, climate even, all of which contribute to a fluctuating, heterogeneous microbiological dynamic whereby all pathogen strains are not necessarily circulating in all countries at all times. As a coarse exploration of between country heterogeneities, we used linear regression to estimate mean MICs and their changes for 50 important PA pairs in each country separately (Supplementary Fig. 22). Some pairs have highly correlated MICs between countries (e.g. *Staphylococcus aureus* and daptomycin) but others vary in MIC by as much as 2 orders of magnitude (e.g. *Streptococcus pneumoniae* and azithromycin), illustrating that ATLAS does capture some geographical heterogeneities.

To better quantify heterogeneities we asked this: do S and R clusters for each PA pair for each year contain data from similar, or different, countries? For instance, could (1) some country ‘A’ have a diversity of MICs and contribute many datapoints to both S and R clusters, or (2) does country ‘A’ tend to contribute data to S and some other country ‘B’ tend to contribute to R? To answer this, we found the Jaccard index between the set of countries that appear in the S cluster and R cluster of a given PA pair in a given year tend to be very similar whereby only 27% of all PA pairs are below 1/2 Jaccard similarity (Supplementary Fig. 24). Thus possibility (1) is more common than (2) which would be explained by different strains circulating stably in different countries at the same time, but (2) does occur.

Despite the issues in data quality we sought clinically relevant predictions by deriving metadata that we queried to detect PA pairs with important properties. For instance, we detected problematic PA pairs where (i) the R-MIC is above the clinical breakpoint and still increasing: *Acinetobacter baumannii* and doripenem is one such pair (Fig. 6D). Conversely, we sought PA pairs where, arguably even more importantly, (ii) the R-MIC is sub-breakpoint but increasing and *Streptococcus pneumoniae* and moxifloxacin is one such case (Fig. 6D). Hoping for optimism, we sought pairs where (iii) the R-MIC is sub-breakpoint and decreasing, like *H. influenzae* and azithromycin (Fig. 6D), and also ones where (iv) the R-MIC is super-breakpoint but decreasing, like *S. aureus* and levofloxacin (Fig. 6D). Importantly, other PA pairs exhibiting property (ii) can be identified (E. coli and *H. influenzae* in Fig. 7A).

Our investigations highlight how raw MICs must be curated carefully, particularly where differently sized databases, covering different regions and different periods in time are merged. Indeed, the rapid R-MIC changes found for CAZ-AVI (Fig. 7A, label C0) may yet prove to be the result of bias because CAZ-AVI has a large representation in ATLAS by design (Supplementary Fig. 6). This design feature could have recruited strains with higher MICs into ATLAS than other databases, increasing its MICs due, for example, to metallo-*β*-lactamase (MBL) and penicillin-binding protein (PBP3) mediating cross-resistance between CAZ-AVI and other antibiotics^62^. A comparison of ATLAS with other raw MIC databases would be needed to establish whether this has unduly biased the rate of ascent of the CAZ-AVI R-MIC seen in ATLAS.

**Fig. 8:**
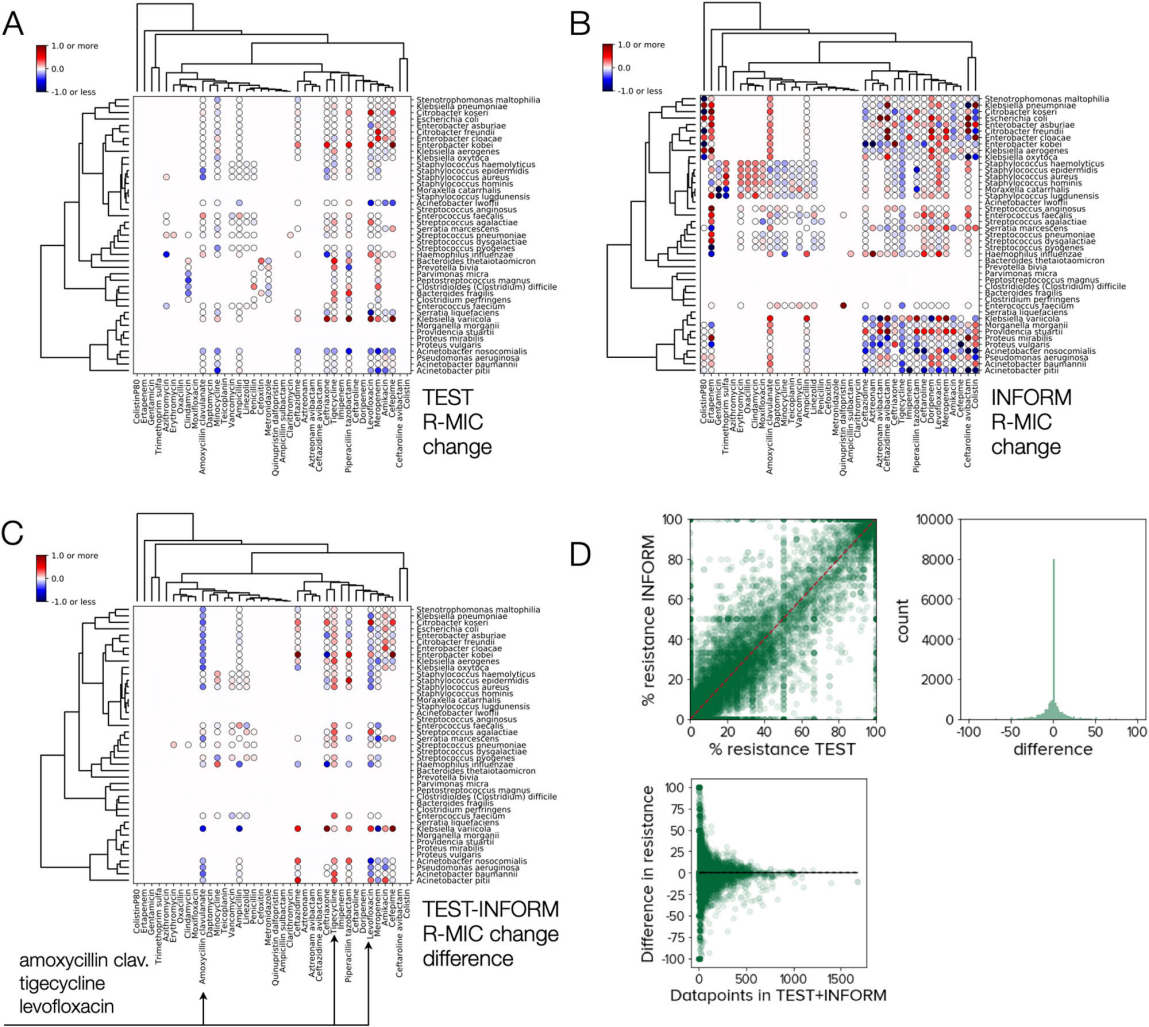
Testing ATLAS for self-consistency by comparing TEST and INFORM predictions separately. **A** Positive (red) and negative (blue) R-MIC changes for all pathogen-antibiotic (PA) pairs in the TEST sub-component of ATLAS were predicted by regressing R-MIC versus time (as Fig. 6B). **B** Predicted positive (red) and negative (blue) R-MIC changes for all PA pairs in the INFORM sub-component of ATLAS. **C** The difference between R-MIC dynamics in TEST and INFORM for those PA pairs that appear in both datasets; the 3 antibiotics with the largest absolute changes are indicated with arrows. **D** Percentage differences between the frequencies of resistance observed in TEST and INFORM for all PA pairs and all years for which data is available. Although some differences are large (as much as 100%) note how the between-database differences decline with increasing numbers of datapoints.

As ATLAS is composed of TEST and INFORM, we checked their mutual consistency: R-MIC dynamics for both sub-databases indicate a tendency towards increased resistance through time (Fig. 8A, B). For PA pairs appearing in both datasets, between-database differences in R-MIC dynamics are greatest for amoxycillin clavulanate (aka co-amoxiclav: amoxycillin combined with clavulanic acid), tigecycline and levofloxacin (Fig. 8C). Moreover, TEST predicts a smaller degree of R-MIC change in co-amoxiclav in comparison with INFORM and the converse is true for the target drug tigecycline (Fig. 8C). Frequencies of resistance statistics have a small but statistically significant between-database bias towards more resistance in INFORM than TEST (skew test with statistic 3.67, *p* ≈ 0.0002, − 0.29% mean difference in frequency of resistance; Fig. 8D).

These observations are noteworthy when we recall that TEST and INFORM were designed to evaluate the activity of tigecycline and CAZ-AVI, respectively, and they may be indicative of testing bias for tigecycline whereby highly resistant strains are over-represented relative to other databases. We speculate that because CAZ-AVI has a high representation in ATLAS due to INFORM (Supplementary Fig. 6) this could have increased R-MICs reported for co-amoxiclav because both are beta lactams with a beta lactamase-inhibiting adjunct, although the biological mechanism for this is unclear.

ATLAS is an imperfect but unique resource because, to the best of our knowledge, it is the only publicly available database containing raw MICs alongside patient metadata and, despite the problems, we find some signals in ATLAS are consistent with prior clinical expectations. Moreover, if more databases of raw MICs were published openly, the issue of data curation artefacts should rescind because more robust mutual consistency checks would then be possible. New features could be explored too, like the recent finding that few clinical MICs exhibit collateral sensitivity between antibiotic pairs^63^ despite its presence in laboratory studies^64,65^. However, databases built on different assumptions should be combined with enough care to ensure automated computational methodologies, like machine learning, do not make spurious inferences. If such checks were implemented, a global MIC database supplemented with time, location and other patient metadata would prove an invaluable resource for understanding resistance patterns that might not be detectable in smaller cohorts.

## Methods

ATLAS data have the following structure: one record represents one patient which comes with metadata, including the year and country of infection, the isolated pathogen strain, whether the latter was deemed resistant, susceptible or intermediate against a panel of antibiotics and it lists all MICs used for those classifications. Data amalgamate different surveillance programmes across approximately 70 countries so there is no uniformity across the database. Following standard clinical practise, each MIC comes un-replicated, with no measure of variation, where the panel of antibiotics assayed vary between patients, even for the same pathogen.

Raw MICs are quoted as scaled powers of 2, *A* ⋅ 2^−*d*^, where *A* is a baseline dose quoted in *μg*/*mL* and typically *d* is the critical two-fold antibiotic dilution found using an antibiotic susceptibility test (AST). Different laboratory protocols and devices estimate the antibiotic sensitivities that contribute to ATLAS, typically broth dilution or disk diffusion tests. To ensure uniformity despite changing CLSI breakpoints, we determine MIC breakpoints by seeking the maximal dose in the database deemed ‘susceptible’ for each PA pair and we fix this value. The latter was not found to differ from the latest CLSI breakpoints^19^. We then divide each MIC by this breakpoint, thus scaling out the dose constant *A*, and we ${\log }_{2}$-transform the result. Physical units for the subsequent MIC are the numbers of twofold dilutions relative to the breakpoint, so a value of “MIC = 0” represents “clinically resistant” throughout. Throughout, wherever the terms ‘MIC’ or ‘${\log }_{2}$MIC’ are written, including in figures, it always refers to the ${\log }_{2}$ value normalised relative to the clinical breakpoint, raw database MIC values are never shown.

All computations in the paper were performed using Pandas 1.4.1 in Python 3 and Matlab 9.11.0.1873467 (R2021b) Update 3 including the Statistics and Machine Learning Toolbox R2021b. Whenever linear regression slopes are reported, they are always statistically significantly non-zero (*p* < 0.05). Non-significant regression slopes are never stated as being non-zero.

### Comparison of open databases

We compared resistance data from the 5 main databases in Table 1 by transforming between datasets in the following way. ATLAS stores patient MICs that can be transformed into the MIC histograms used by EUCAST by removing space and time metadata, thus forming a single histogram of MICs (e.g. Fig. 2B, C). For consistency with ATLAS, EUCAST MIC histograms were scaled relative to the latest CLSI breakpoint^19^ and then ${\log }_{2}$ transformed.

It is not possible make the converse comparison of EUCAST with ATLAS because we cannot transform EUCAST data into the datatype that ATLAS holds, this is because EUCAST has no spatiotemporal metadata. Similar comments apply to ESPAUR, ECDC and Resistance Map databases because none of these contain the raw MIC data from which their frequencies of resistance were originally inferred.

The frequency of resistance data stored in ESPAUR, ECDC and Resistance Map databases can be compared with ATLAS by transforming raw MICs of the latter into frequencies of resistance in a defined geographical region for a defined time period using the latest CLSI breakpoints^19^.

### MIC variation: additive noise model

Following pre-processing and as is standard for accredited clinical microbiological testing laboratories, ATLAS MICs are discrete data quoted without uncertainty measures or replication. To test the robustness of our analyses we therefore created *n* = 50 synthetic replicates of ATLAS to provide uncertainty estimates, as follows. We simulated within-replicate MIC variation of the kind generated by laboratory assays by adding a stochastic quantity to each MIC which we sampled from a normal distribution with a variance of 1 dilution. This approach permits a form of sensitivity analysis whereby patterns of resistance and resistance change can be classified as significant, or not, relative to this degree of uncertainty in MIC. N.B. Comparisons of ATLAS data with other databases that only contain frequencies of resistance were not performed with any added noise (e.g. Fig. 1A).

### Determination of S and R and their time derivatives

We used Gaussian mixture models to determine the optimal number of clusters in the MIC distribution for each PA pair and these models identified the cluster with greatest mean MIC, which we call the R-cluster, or just R. The R-MIC is then defined to be the mean of the MICs in the R-cluster. The lower MIC boundary of the R cluster defines the upper MIC boundary of an ‘S’ (meaning susceptible) sub-population and this was defined using an equal likelihood criterion: pathogen MICs were labelled ‘S’ if the estimated probability of residing in the R cluster was less than 1/2. Linear regressions were then used to determine MIC change versus time for the S-MIC and R-MIC (Figs. 6A/D and 7C). This procedure is likely to be more accurate for R-MIC data because the latter is formed from a single normally distributed cluster whereas S-MICs derive from a sum, potentially, of several normal distributions. In terms of nomenclature, it is possible that some members of the S subpopulation, in fact, exhibit clinical resistance where the R cluster has very high levels of clinical resistance but this is not typically the case in practice.

### Reporting summary

Further information on research design is available in the Nature Research Reporting Summary linked to this article.

## Supplementary information

Supplementary Information

Reporting Summary

## Data Availability

ATLAS is available following website registration^*^. Data and further information can be downloaded from the following links: Project overview: https://amr.theodi.org/project-overview Project description: https://wellcome.ac.uk/sites/default/files/antimicrobial-resistance-surveillance-sharing-industry-data.pdf Data download^*^: https://www.synapse.org/#!Synapse:syn17009517/wiki/585653 The same dataset is available from this link: https://s3-eu-west-1.amazonaws.com/amr-prototype-data/Open+Atlas_Reuse_Data.xlsx Data was extracted from the English Surveillance Programme for Antimicrobial Utilisation and Resistance (ESPAUR) report from years 2013-2018. These were downloaded from the following UK government website: https://www.gov.uk/government/publications/english-surveillance-programme-antimicrobial-utilisation-and-resistance-espaur-report ResistanceMap data is published by the Centre for Disease, Dynamics Economics and Policy^28^, it can be downloaded from https://github.com/gwenknight/empiricprescribing/tree/master/data, Data for the European Centre for Disease Prevention and Control (ECDC) can be downloaded from https://atlas.ecdc.europa.eu/public/index.aspx?Dataset=27#x00026;HealthTopic=4. The file we used in this paper can be downloaded from https://github.com/PabloCatalan/atlas/tree/master/data/europe_resistance_data.csv EUCAST data can only be obtained by contacting individuals named on their website https://www.eucast.org/mic_distributions_and_ecoffs/ and requesting access to MIC histograms, which we were granted.

## Code availability

Analysis codes^66^ written in Python 3.0 using pandas can be downloaded here: https://github.com/PabloCatalan/atlas or 10.5281/zenodo.6390565.

Codes have been written to provide straightforward access to data so that figures from this manuscript can be reproduced and to help facilitate the development of new analyses. Interested readers are encouraged to seek assistance from corresponding authors in case it is not clear how those codes are used.
