# Supplementary material for: Seeking patterns of antibiotic resistance in ATLAS, an open, raw MIC database with patient metadata
Source: Nat Commun. 2022 May 25;13:2917. doi: 10.1038/s41467-022-30635-7 (PMC9133080; doi:10.1038/s41467-022-30635-7)
Supplement: Supplementary file 1 — Supplementary Information [file 41467_2022_30635_MOESM1_ESM.pdf]

Supplementary Information

Clinical breakpoints were obtained from the 2018 CLSI guidelines.[19]

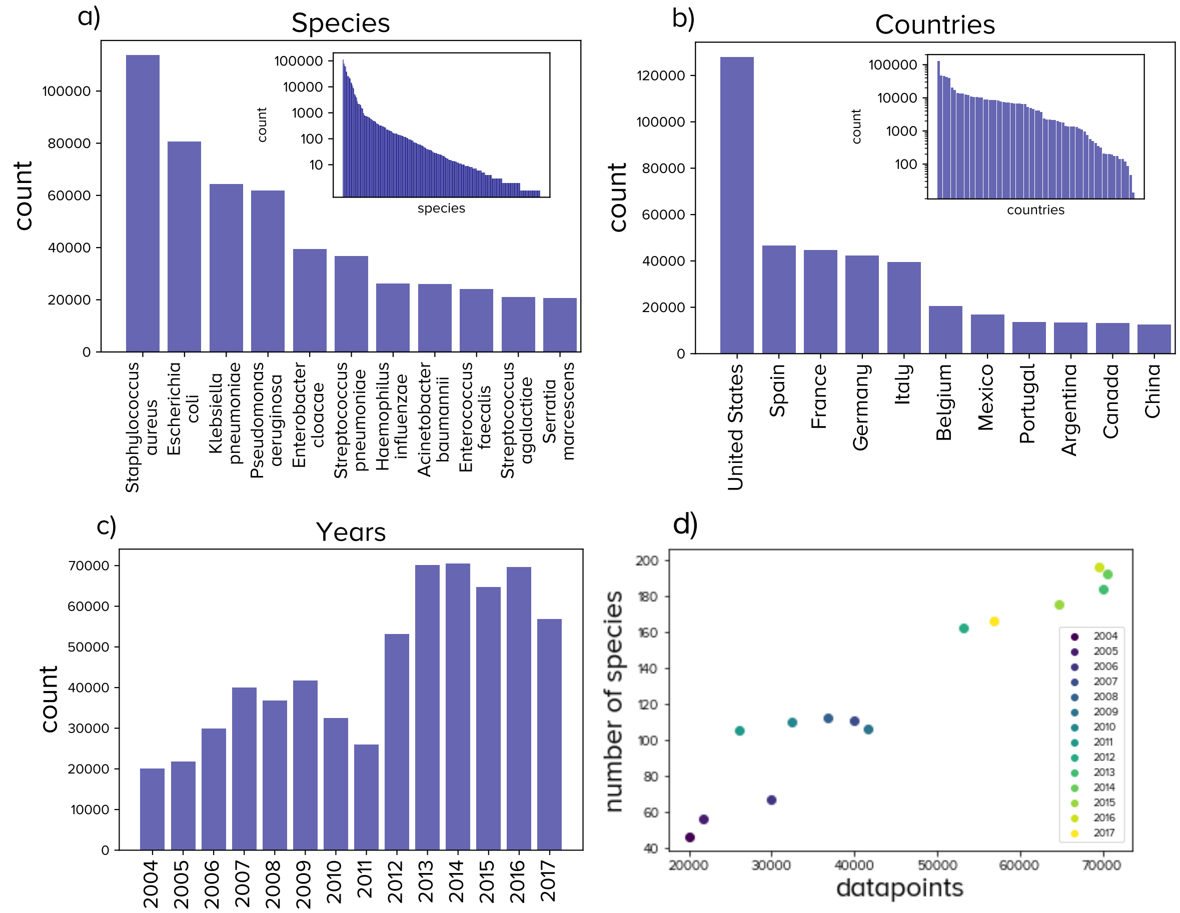

Supplementary Figure 1: Basic statistics of the ATLAS database. A) The bacterial species most represented in ATLAS. B) Some countries contribute more data than others, even after accounting for population size. C) More MIC data has been added to the ATLAS database in recent times with a noticeable increase around 2012. D) An analogous comment applies to bacterial species: these increase in number through time. These features are explained below.

1 A methodological description of ATLAS

The Antimicrobial Testing Leadership and Surveillance (ATLAS) programme, led by Pfizer, is the union of 3 previous surveillance programs: the Tigecycline Evaluation Surveillance Trial (TEST) program, instigated by Pfizer in 2004, the International Network for Optimal Resistance Monitoring (INFORM)

program, effected by AstraZeneca from 2012 to 2017, and the Assessing Worldwide Antimicrobial Resistance Evaluation (AWARE) program, also run by AstraZeneca from 2008 until 2017. All three programmes were combined to form ATLAS in 2018. However, in the public dataset used for this work, only isolates from TEST and INFORM were included. Out of the $633,820$ isolates in ATLAS, $218,432$ (around 34%) belonged to the INFORM program and the remaining 66% were TEST isolates.

A key feature of TEST was to evaluate the activity of the broad-spectrum antibiotic tigecycline and a panel of other comparator agents against a set of clinically relevant bacterial pathogens.[67] Medical centres, typically hospitals, contributing to the programme were required to submit at least 65 Gram-positive isolates and 135 Gram-negative isolates each year. An additional requirement was a minimum of 25 *S. aureus*, 15 *Enterococcus spp.*, 15 *S. pneumoniae*, 10 *Streptococcus agalactiae*, 25 *Klebsiella spp.*, 25 *E. coli*, 25 *Enterobacter spp.*, 20 *Pseudomonas aeruginosa*, 15 *Acinetobacter spp.*, 15 *H. influenzae* and 10 *Serratia spp.* isolates.[67]

Isolates were obtained from all body sites and submitted as probable cause of infection (both community and hospital-acquired) to a single laboratory. International Health Management Associates (IMHA) collated organisms and confirmed their identification. MICs were determined at each contributing centre using CLSI broth dilution methodology[19] in conjunction with either MicroScan panels (DadeMicroscan Inc., West Sacremento, CA, USA) or Sensititre plates (TREK Diagnostic Systems, East Grinstead, UK).[68] For more details on TEST protocols, see.[67,68] The INFORM programme set out to study the activity of ceftazidime-avibactam and several comparator agents and had similar characteristics to TEST.[69,70]

2 Quotations from ATLAS curators

ATLAS data is derived from specimens sent routinely to clinical laboratories and aligns with the World Health Organization's Global Antimicrobial Resistance Surveillance System (GLASS) protocol metrics.[31] To evidence this, we have included quotations from the ATLAS documentation held at the following webpage:

https://wellcome.org/sites/default/files/antimicrobial-resistance-surveillance-sharing-industry-data.pdf

On page 8 (Relationship to other surveillance data):

> *Importantly, the data collected by pharmaceutical surveillance programmes is based on priority specimens sent routinely to laboratories and therefore aligns most closely with the World Health Organization's Global Antimicrobial Resistance Surveillance System (GLASS)*

protocol metric Proportion of samples with growth of non-susceptible bacteria of the species and antibiotic under surveillance per specimen type.

On page 7 (Isolate collection):

These participating laboratories submit isolates from infected patients originally collected as part of routine clinical practice.

3 Testing for systematic bias in ATLAS

3.1 ATLAS Methodology

TEST and INFORM surveillance programs were designed to evaluate the activity of new agents tigecycline and ceftazidime-avibactam (CAZ-AVI) against resistant isolates. As a result, it is possible that contributing centres may have chosen to send more resistant isolates for testing, resulting in a systematic bias towards resistance in ATLAS. This is, indeed, suggested by the observation that ATLAS MICs tend to be higher than those of the ResistanceMap, ESPAUR and ECDC databases (Figure 1). If this is the case, then a subsequent analysis based on ATLAS data would be compromised. However, we argue that there is no systematic bias towards resistance and we do so using the following statistical model.

First, we create a model database that exhibits systematic MIC bias. For this, suppose we have a large bacterial population with two genotypes, one susceptible (S) and the other resistant (R). As in the main text, assume the clinical MIC breakpoint is at 0, so R isolates will have positive MICs and S isolates have negative MICs. Assume these MICs are normally distributed and, to create the data, suppose the average values of MIC distributions are chosen uniformly between -4 and -1 for S isolates and 0 and 3 for R isolates. Meanwhile, standard deviations of these distributions will be chosen uniformly between 0.1 and 1.

We can sample this model bacterial population with a bias which is simply this: if contributing centres were biased towards sending more resistant isolates for testing, then the probability of choosing a resistant isolate should be higher than the probability of choosing a susceptible isolate. There are many ways to model this but one method is to choose an isolate for submission with probability density

$$p(x) = \frac{1}{Z}\frac{x-m}{1+a(x-m)}, \tag{1}$$

where x is the MIC of the isolate (mimicking ATLAS where $-10 \leq x \leq 10$), m is the minimum MIC in the population, $Z = \int_{-10}^{10} \frac{x-m}{1+a(x-m)}dx$ is a normalising factor and $a \neq 0$ is a parameter that modulates the strength of bias: $a = 0$ creates a linear bias, while $a \to \infty$ results in unbiased sampling.

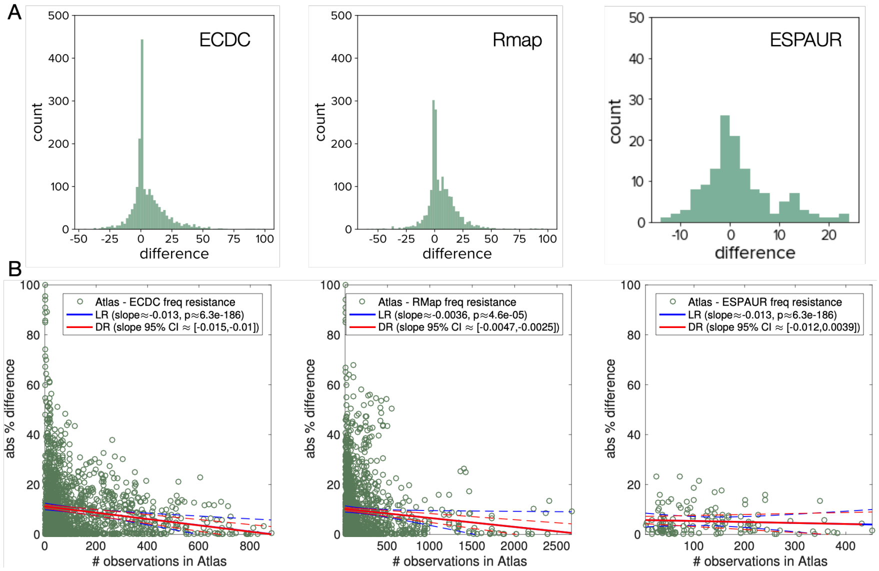

Supplementary Figure 2: Histograms comparing PA pair data 'pointwise' against ATLAS asking: are between-database differences skewed? Frequencies of resistance determined from ATLAS MICs are compared with Resistance Map, ECDC and ESPAUR 2013-2018 (the latter compared only against UK data in ATLAS) PA pair datasets by taking the absolute difference between the frequency for each PA pair. Each dot represents a pathogen-antibiotic (PA) pair, in a given country, for a given year. A) This shows differences between the %age of resistant cases in ATLAS and other databases are significantly positively skewed (p-values using `scipy.stats.skewtest` are: for ATLAS against RMap: 10^{-87}, against ECDC: $8 \cdot 10^{-63}$ and against ESPAUR: $2.68 \cdot 10^{-04}$). B) However, this shows between-database differences are smaller for larger PA pair datasets: linear regressions (LR in legend) show a significant negative correlation between the number of datapoints (x-axis) and between-database differences in the fraction of resistant bacteria sampled (y-axis) (F-test, p-values< 0.001). Thus datasets converge as the number of samples increases. Label DR (red lines) shows analogous behaviour for Deming regression although the ATLAS-ESPAUR comparison is not significantly decreasing for that methodology (95% CIs defined in the inset contain zero).

Parameter a is chosen with an exponential distribution $p(a) = \lambda \exp^{-\lambda a}$. This sampling strategy ensures that resistant isolates are chosen more often than not. Finally, the sample size N is chosen uniformly between 10 and 1000, where the population has $100,000$ individuals for computational tractability.

Now we perform a simulation that generates $1,000$ bacterial populations and we sample them using the procedures described above. We then calculate the resistant fraction of the sample and compare

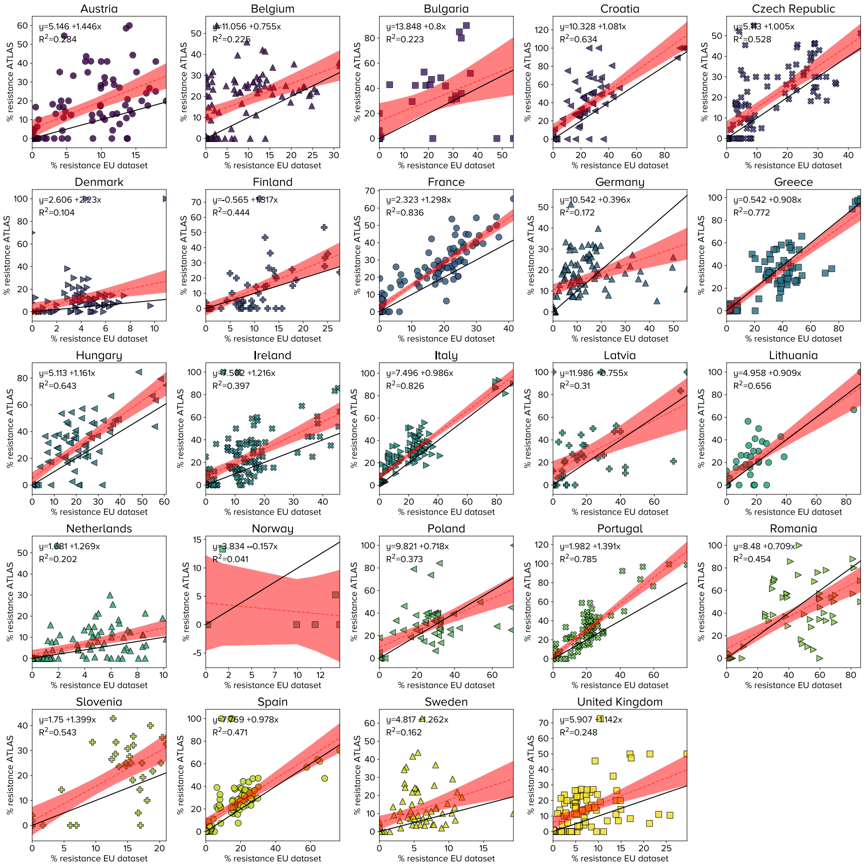

Supplementary Figure 3: Collated ECDC and ATLAS data and their correlations for European countries. Linear regressions indicate ATLAS reports higher frequencies of resistance than ECDC data but only in some countries (each symbol denotes one PA pair). The linear regressions, which should report $y = x$ for each country can, instead, yield $y = ax + b$ where fitting parameters (a, b) are significantly different to $(1, 0)$ in several cases. Shaded area in red represents the 95% confidence interval of the mean.

them with the true resistant fraction in the population. The idea is to compare features of synthetically generated data with properties of ATLAS shown, for instance, in Figure 1A and Supplementary Figure 2. To make this comparison, we assume the sampled populations are those in ATLAS and the 'true' frequencies of resistance are those found in ResistanceMap, ESPAUR or ECDC databases.

The results of this are summarised in Supplementary Figure 4. Notice that there is a consistent overestimation of resistance values in the model samples (Supplementary Figure 4(left) and (middle)) which is not what we observe when we compare ATLAS versus ResistanceMap, ESPAUR and ECDC (Figure 1A). Importantly, there is no relationship between sample size and the absolute difference

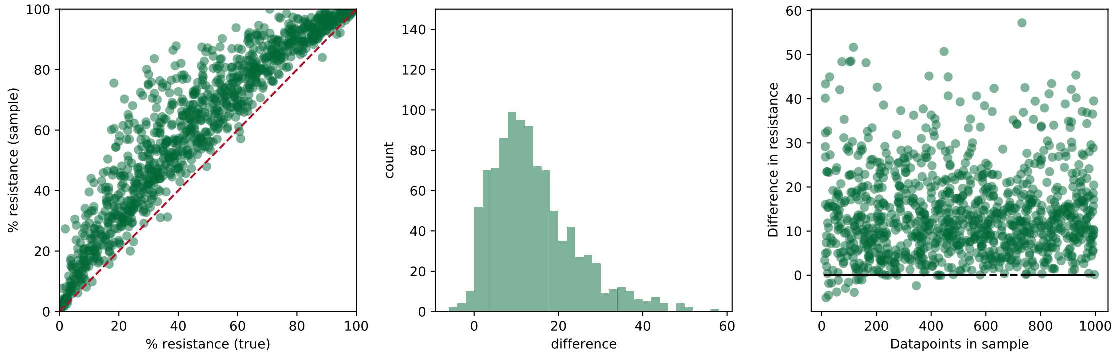

Supplementary Figure 4: Testing for systematic resistance bias in ATLAS. A simple statistical model illustrates features of systematic database bias towards having more resistant isolates. (left) Comparing the resistant fraction calculated for a synthetic database of biased samples naturally results in a consistent overestimation of resistance. Compare this to Figure 1A where resistance bias does not appear to be systematically skewed. (middle) Here, the distribution of differences between database and its biased sub-sample is systematically biased towards positive values, with little underestimation. (right) Importantly, there is no relationship between sampling size and the difference between estimated and true resistance, this is at odds with what we observe in ATLAS: Supplementary Figure 2B shows greater differences between datasets for smaller samples whereas the statistical model used here does not.

between estimated and true resistance values in the synthetic data (Supplementary Figure 4(right)), whereas ATLAS samples were significantly more likely to be different from ResistanceMap, ESPAUR or ECDC if their sample size was smaller (Figure 1A and Supplementary Figure 2). From these observations we conclude that ATLAS is not systematically biased and differences in estimating MICs between real-world databases, therefore, may be due to the fluctuations inherent to comparing small sample sizes.

There are many limitations in the above argument, particularly its lack of generality that uses one model of systematic bias. Nevertheless, one also can illustrate that even if there is a systematic sampling bias towards resistance, R-MIC dynamics can have a form of robustness against it. To illustrate, consider a population with 2 sub-populations, S and R, as before. Suppose the MIC of both is normally distributed: the mean MIC of the susceptible subpopulation (S, 75% of the population) is defined to be -2 and the resistant one, the R-MIC, is 1, while both have unit variance. Suppose the MIC of both evolves linearly with time: the S-MIC decreases at 0.05 units per year, while the R-MIC increases at 0.175 units per year. Suppose the population has 100,000 isolates and we take a biased sample of 1,000 isolates, with the same systemic biasing method as before (using (1)).

We then take this sample and extract the R cluster using a Gaussian mixture model and compute its R-MIC trend as is done in the main text, the results are shown in Supplementary Figure 5. In this example based on synthetic data, the inferred trend is 0.179 MIC units per year (against a baseline of 0.175), while the inferred intercept is 1.067 (against 1.000). Baseline trends (pre-sampling bias) are plotted as dashed lines (red for R, blue for S), while estimated trend (post-bias) is plotted as solid red line.

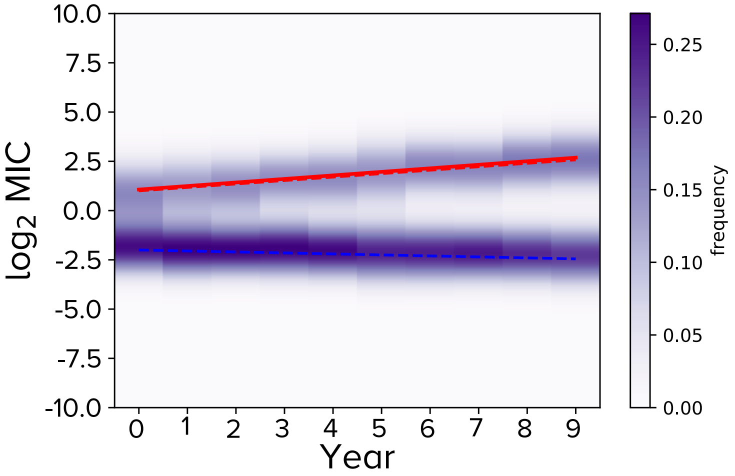

Supplementary Figure 5: Dynamics of the R-MIC need not be affected by sampling bias towards resistance. The procedure of applying linear regression to the R-cluster of Gaussian mixture models as used in the main text to detect changes in high levels of resistance was applied to a synthetic population consisting of S and R clusters. While over-sampling R and under-sampling S can lead to changes in predicted trends of the S-MIC, the R-MIC is more robust to this bias: here, the 2 almost indistinguishable red lines (one is dashed, one is thick) indicate predicted dynamics of the R-MIC pre- and post-implementation of the bias that skews the sampled strains towards greater resistance. This example illustrates that this bias need not unduly affect R-MIC dynamics even though the S cluster could disappear entirely due to this bias.

This argument could be generalised in a number of ways, but the principle this synthetic exemplar illustrates is that R-MICs are less affected by sampling bias towards greater MICs than are the S-MICs of the population. The reason for this is clear: S strains are removed from the sampled population with greater likelihood than are R strains by positive bias towards greater resistance in the sampling methodology. For completeness, §3.2 below contains one such generalisation.

3.2 Systematic R-biased sampling yields expected differences between the population and sampled fraction of resistance that are independent of sample size

Let $\mathcal{X} \subset \mathbb{R}$ be an interval representing the possible MICs of a bacterial population with two subpopulations, S (susceptible) and R (resistant). The MIC of the population has the following probability density function

$$f(x) = (1-r)f_S(x) + rf_R(x),$$

where r is the fraction of R individuals in the population, $f_S(x)$ and $f_R(x)$ are the MIC distributions of the S and R subpopulations, respectively, where $x \in \mathcal{X}$ is an MIC. For definiteness, although it is an abuse of notation, let $R \subset \mathcal{X}$ be an interval containing the highest MIC in $\mathcal{X}$ and let S be the complement of R in $\mathcal{X}$.

Now sample the population with a bias towards R whereby the probability of introducing a resistant bacterium into the sample if it belongs to R is $p > 1/2$ and, accordingly, the probability of introducing a susceptible bacterium into the sample is $1-p$. This is a negative binomial sample and we will continue to sample until we obtain N individuals.

The probability density function of the negative binomial distribution, which gives the probability that the number k of "sample trials" has a certain value given some acceptance parameters, is known, but of no interest in the following calculation. Instead, for our purposes, we simply need to know that we construct some sample set, $\mathcal{X}_N = \{x_1, x_2, \ldots, x_N\}$, with a different MIC distribution from the original population due to sampling. In particular, the fraction of resistant individuals in the sample will no longer be r, but a random variable r'. We now want to prove that the expected difference between r and r' is independent of N, thus showing that a systematic bias towards R would not be consistent with ATLAS data in Supplementary Figure 2B but it would be consistent with synthetic computational data in Supplementary Figure 4(right).

Now, the fraction of resistant individuals in the sample $\mathcal{X}_N$ is given by

$$r' = \frac{1}{N}\sum_{i=1}^{N} \mathbf{1}_R(x_i),$$

where $\mathbf{1}_R(x_i)$ is the indicator function of R: it is equal to one if $x_i \in R$, and it is zero otherwise. Thus

$$\mathsf{P}\left(\mathbf{1}_R(x_i) = 1\right) = \mathsf{P}(x_i \in R \mid \text{sample } i \text{ is in the sample set}),$$

and we have to determine this probability.

The sampling process has two steps: first, we sample from the population uniformly (i.e. randomly) with probability r of choosing an R individual (this is a Bernoulli trial with parameter r). Then, we

include the sampled bacterium into our sample set with probability p if its MIC belongs to R (another Bernoulli trial with parameter p). Therefore, using Bayes' theorem,, writing probabilities informally for pedagogical reasons,

$$\begin{aligned} \mathsf{P}\left(\mathbf{1}_R(x_i)=1\right) &= \mathsf{P}(x_i \in R \mid \text{sample } i \text{ is in the sample set}) \\ &= \frac{\mathsf{P}(\text{sampling } R)\ \mathsf{P}(\text{including } R \text{ in the sample set})}{\mathsf{P}(\text{inclusion in sample set})} \\ &= \frac{rp}{rp+(1-r)(1-p)}, \end{aligned}$$

because

$$\mathsf{P}(\text{inclusion in sample}) = \mathsf{P}(\text{sampling } R)\ \mathsf{P}(\text{including } R \text{ in sample}) +$$

$$+\mathsf{P}(\text{sampling } S)\ \mathsf{P}(\text{including } S \text{ in sample}) = rp+(1-r)(1-p).$$

Therefore, $\mathbf{1}_R(x_i)$ is a Bernoulli trial with parameter $t = \frac{rp}{rp+(1-r)(1-p)}$.

Defining $Z = \sum_{i=1}^{N} \mathbf{1}_R(x_i)$, it is clear that Z is a Binomial random variable with parameters (N,t) as the x_i are independent. Therefore,

$$\text{Prob}(Z=k) = \binom{N}{k} t^{N-k}(1-t)^k,$$

and

$$\mathbb{E}(r') = \mathbb{E}(\frac{1}{N}Z) = \frac{1}{N}Nt = t,$$

which is independent of N, concluding the argument. To re-iterate, this calculation is not consistent with ATLAS data in Supplementary Figure 2B but it is consistent with Supplementary Figure 4(right), we conclude that ATLAS is not consistent with systematic bias towards greater sampling of resistant bacteria. Finally, note that when $p = 1/2$, so there is no bias towards sampling resistant bacteria, $\mathbb{E}(r') = t = r$.

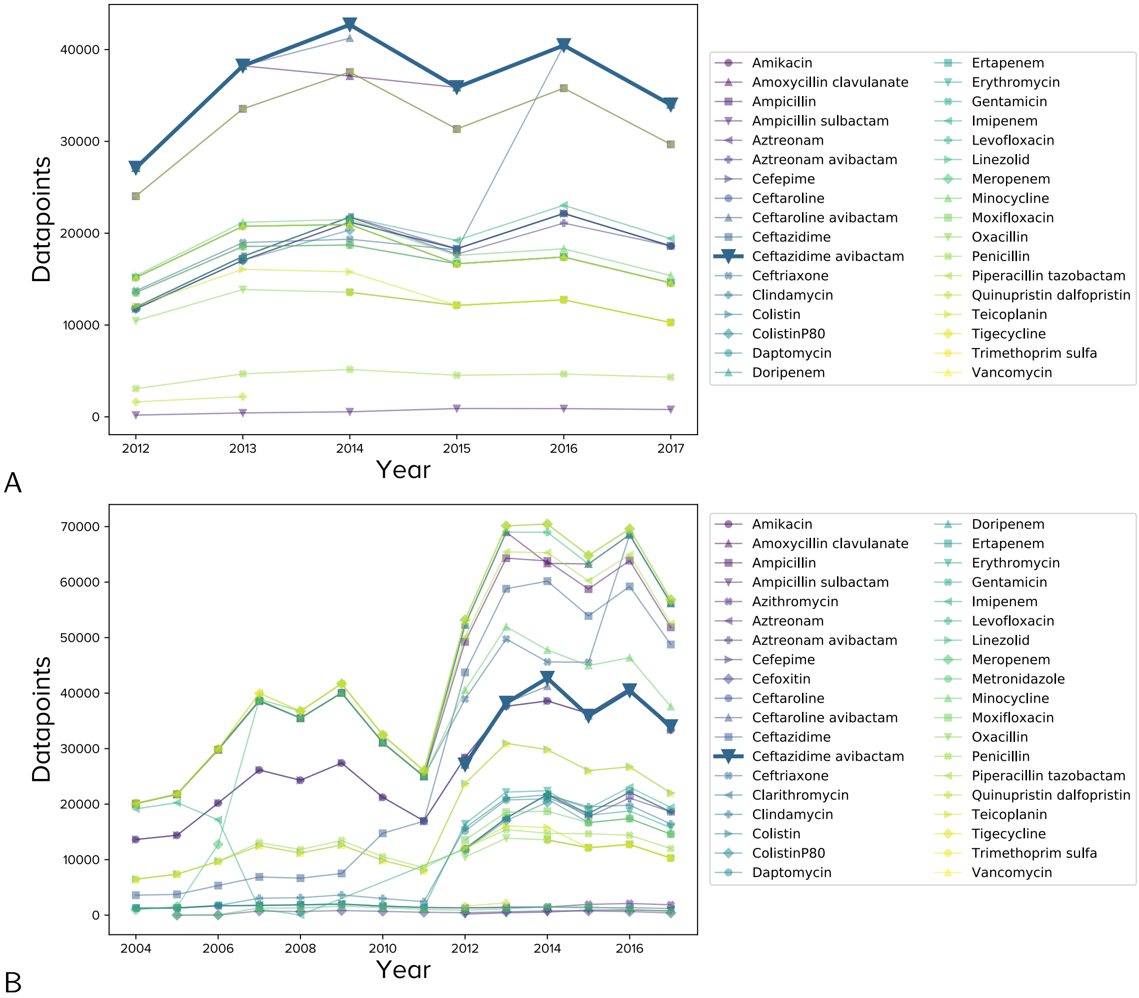

Supplementary Figure 6: The number of data points through time for CAZ-AVI (ceftazidime avibactam) that contribute to the ATLAS database. A) Data from the INFORM dataset only. B) Data from the entirety of ATLAS. In both A and B, data for CAZ-AVI are highlighted using a bold line. Note how CAZ-AVI is the most-represented antibiotic in INFORM (see A) and has over half the number of the most-represented antibiotics in ATLAS (see B) even though it is not as highly-prescribed as many other antibiotics.

4 Changepoint analysis for low-MIC sampling bias in ATLAS

Given a time series consisting of a number of MICs, $n_1, \ldots, n_T$ say, we want to test the null hypothesis, H_0, that all sampled points come from the same distribution against the alternative hypothesis, H_1, that there is a single changepoint in mean, μ, at timepoint τ:

$$\begin{aligned} H_0 &: \mu_1 = \mu_2 = \cdots = \mu_{T-1} = \mu_T \\ H_1 &: \mu_1 = \mu_2 = \cdots = \mu_{\tau-1} = \mu_\tau \neq \mu_{\tau+1} = \mu_{\tau+2} = \cdots = \mu_{T-1} = \mu_T. \end{aligned}$$

Assuming that the sample is normally distributed and that the variance, σ^2, does not change across the sample, the log-likelihood ratio between the two hypotheses is:

$$\mathcal{R}_\tau = \log\left(\frac{\mathcal{L}_{H_1}}{\mathcal{L}_{H_0}}\right) = -\frac{1}{2\sigma^2}\left[\sum_{i=1}^{\tau}(n_i - \mu_1)^2 + \sum_{j=\tau+1}^{T}(n_j - \mu_2)^2 - \sum_{k=1}^{T}(n_k - \mu_0)^2\right], \tag{2}$$

where $\mu_0 = \sum_{i=1}^{T} n_i/T$, $\mu_1 = \sum_{i=1}^{\tau} n_i/\tau$ and $\mu_2 = \sum_{i=\tau+1}^{T} n_i/(T-\tau)$. We then find the value of τ that maximizes $\mathcal{R}_\tau$, and we define

$$G = \max_{1\leq\tau\leq T} \mathcal{R}_\tau \tag{3}$$

where $\hat{\tau} = \underset{1\leq\tau\leq T}{\text{argmax}}\ \mathcal{R}_\tau$. We accept the existence of a changepoint, at time $\hat{\tau}$, if G is larger than a critical value, λ^*, defined by the Bayesian Information Criterion[71] $\lambda^* = \frac{1}{2}\log T$.

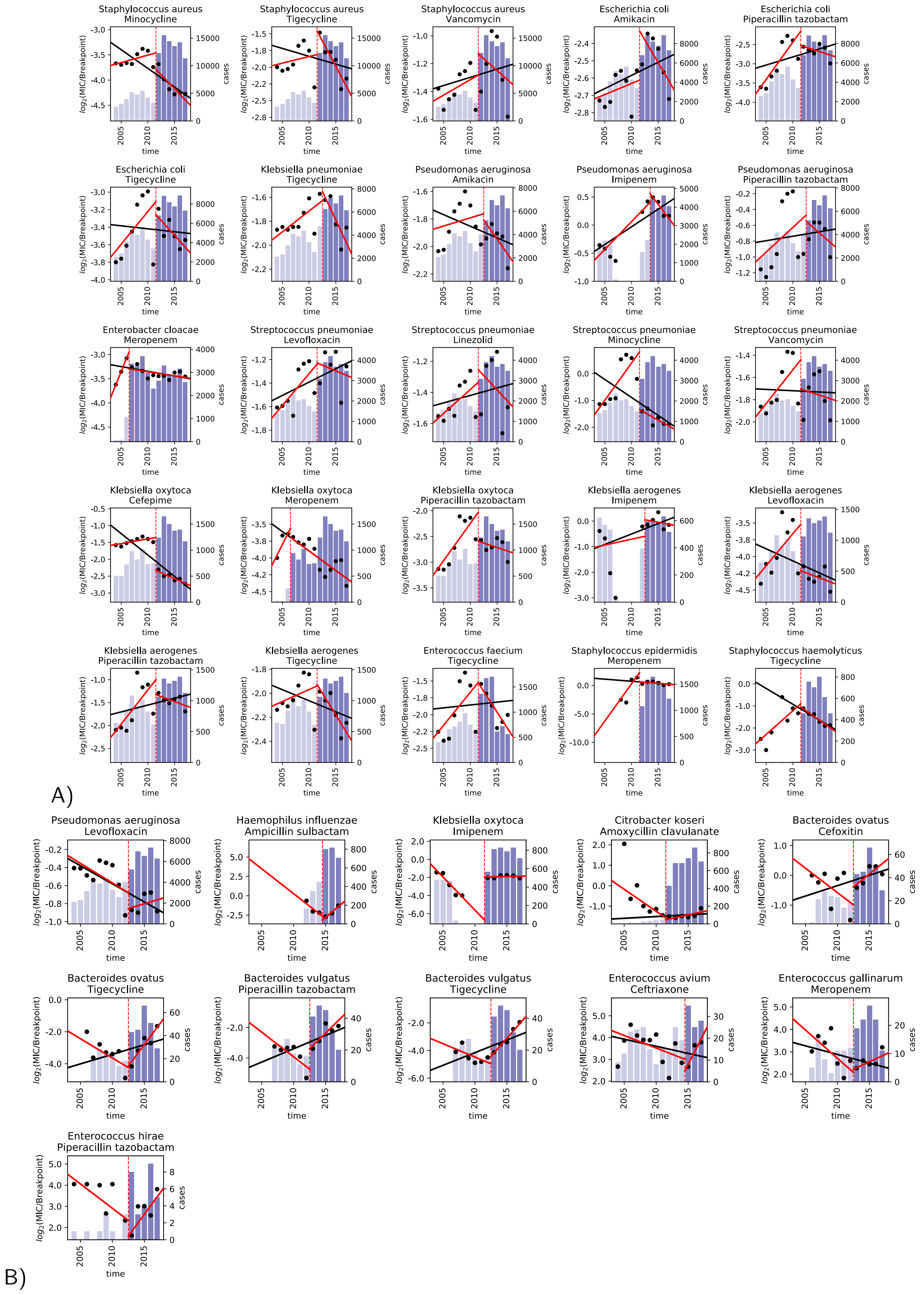
Staphylococcus aureus Minocycline
Staphylococcus aureus Tigecycline
Staphylococcus aureus Vancomycin
Escherichia coli Amikacin
Escherichia coli Piperacillin tazobactam
Escherichia coli Tigecycline
Klebsiella pneumoniae Tigecycline
Pseudomonas aeruginosa Amikacin
Pseudomonas aeruginosa Imipenem
Pseudomonas aeruginosa Piperacillin tazobactam
Enterobacter cloacae Meropenem
Streptococcus pneumoniae Levofloxacin
Streptococcus pneumoniae Linezolid
Streptococcus pneumoniae Minocycline
Streptococcus pneumoniae Vancomycin
Klebsiella oxytoca Cefepime
Klebsiella oxytoca Meropenem
Klebsiella oxytoca Piperacillin tazobactam
Klebsiella aerogenes Imipenem
Klebsiella aerogenes Levofloxacin
Klebsiella aerogenes Piperacillin tazobactam
Klebsiella aerogenes Tigecycline
Enterococcus faecium Tigecycline
Staphylococcus epidermidis Meropenem
Staphylococcus haemolyticus Tigecycline
A)
Pseudomonas aeruginosa Levofloxacin
Haemophilus influenzae Ampicillin sulbactam
Klebsiella oxytoca Imipenem
Citrobacter koseri Amoxycillin clavulanate
Bacteroides ovatus Cefoxitin
Bacteroides ovatus Tigecycline
Bacteroides vulgatus Piperacillin tazobactam
Bacteroides vulgatus Tigecycline
Enterococcus avium Ceftriaxone
Enterococcus gallinarum Meropenem
Enterococcus hirae Piperacillin tazobactam
B)
log2(MIC/Breakpoint)
cases
time

Supplementary Figure 7: A data size changepoint analysis detects PA pairs for which temporal decreases in MIC coincide with increased data availability. A) Each sub-plot shows analogous data: first, the blue bars represent the yearly number of available datapoints ('cases') for each PA pair and the change from light to dark blue highlights the year at which an changepoint was detected in the number of available datapoints in ATLAS. The two red lines show the slopes of linear regressions that indicate significant increases in MIC before the changepoint (the vertical dashed red line) followed by a significant decrease in MIC after the changepoint. Finally, the black line is the result of applying a regression to the entire MIC timeseries to estimate a coarse rate of change. Thus, these PA pairs represent cases whereby a predicted change in MIC correlates with an increase in data availability. B) Analogous to A) but these PA pairs have MICs that first decrease and then increase at a data size changepoint.

5 Spectral Analysis of Correlation Matrices

Given the lack of replication in clinical MIC assays, it is important to ask whether MIC timeseries from the ATLAS database are noise-like or do they form coherent signals with relatively small noise despite being sampled from different patients, at different times in different locations? We therefore use a test for MIC coherency between years using the following null model: we ask, what within- and between-year correlations would be found in MICs if they were drawn from uncorrelated processes each year? We then test the Frobenius norm of the correlation matrix of each PA pair for consistency with that null.

So, assume a data stream, (t_k, m_k), of MIC values (at time t_k) is taken for N years for a given PA combination, where i and j are labels for two years and where each MIC is a random variable with $m_k \sim \text{PDF}(f)$ where f is a probability density function that does not depend on year and F is its cumulative distribution function supported on the discrete set $\{-10, -9, -8, ..., 8, 9, 10\} =: M$, where ± 10 is a clinically reasonable upper and lower bound for MIC observations on the $\log_2$ MIC scale defined in Methods.

Now bin data according to the year associated with t_k and let y_j be the sampled MIC distribution for year j, and so we expect $y_j \approx f$ as a vector supported on M (of dimension 21, the cardinality of M). Centralise the sample distribution data, $Y_j = (y_j - \text{mean}(y_j))/\text{std}(y_j)$, so each Y_j is a random variable with some distribution function $Y_j \sim \text{PDF}(g)$ where g derives from f and is positive, takes on continuous values in $[0, 1]$ and is supported on $2 \cdot M$. Now set $\hat{y}_{ij} := E(Y_i - Y_j)^2 = 2(1 - E(Y_i Y_j))$ and let $X = (1 - \hat{y}_{ij}/2)$ be a correlation matrix which is symmetric with positive entries between 0 and 1. Moreover X can be written $X = I_N + L + L^T$ where L is a lower diagonal matrix with entries between 0 and 1 where I_N is the identity matrix.

If yearly MIC data is correlated because MICs can be modelled as a stationary random variable with the same distribution each year, then Y_i and Y_j both have g as their PDF. Thus $X = \texttt{Ones}(N)$, which is the $N \times N$ rank 1 'unity matrix' consisting only of 1 in each of its ij entries. On the other hand, if yearly MICs have been drawn each year from independent random variables then $E(Y_i Y_j) = 0$ and $X = I_N$, the $N \times N$ identity matrix.

Intuitively, if all MICs of a PA pair are drawn from a stationary MIC distribution then X will be a matrix of rank 1. If MICs are drawn from non-stationary distributions of MICs subject to evolutionary change because bacteria are evolving in response to antibiotic use, we should expect some changes in between-year MIC distributions, so X will have rank greater than 1. In the extreme case that there is such uncertainty from clinical procedures concerning MIC measurements, and the fact that different

assays are used to obtain MICs, that the MIC is a noise-dominated process, then X should have rank N.

We will now test these algebraic properties of X, the matrix associated with an arbitrary MIC dataset for some PA pair, as defined above. The Frobenius norm of X, $\|X\|_F := \text{tr}(X^TX)^{1/2}$, where tr denotes a matrix trace, is helpful in this regard. The eigenvalues of X are counted according to algebraic multiplicity for the following eigenvalue sums. So, if $\sigma(A)$ denotes the spectrum of a matrix, A, then yearly MICs are uncorrelated when $\sigma(X) = \{1, 1, ..., 1\}$ and rank$(X) = N$ but they are correlated when $\sigma(X) = \{1, 0, ..., 0\}$ and rank$(X) = 1$. Now singular values of X, $s(X)$, satisfy $s(X) = \sigma(X^TX) = \sigma(X)^2$ and

$$\sum s(X) = \sum \sigma(X^TX) = \text{tr}(X^TX) \leq N^2$$

because each element of X can be bounded by the Cauchy-Schwartz inequality under assumptions of the null, where $\text{tr}(X^TX)$ is the sum of squares of all the entries in X. Also

$$\sum s(X) = \sum \sigma(X^TX) \geq N$$

because the N diagonal entries of X are all 1.

Given this, we now define functionals τ and τ_N that can be used as the basis of a test that compares correlation matrices for PA pairs independently of the value of N over which the MIC data stream has been gathered. As $X = L + I + L^T$, where L is a lower-diagonal matrix with entries between 0 and 1, then we are interested in whether the mean square off-diagonal entries in L, namely the mean over all l_{ij}^2, is 0, 1, or neither. Now, this mean square, noting $\text{t}r(X) = \text{t}r(I) = N$, is

$$\frac{1}{N^2 - N}\sum_{i \neq j}^{N} l_{ij}^2 = \frac{1}{2(N^2 - N)}\sum_{i > j}^{N} l_{ij}^2 + l_{ji}^2 = \frac{\text{t}r((L + L^T)^T(L + L^T))}{2(N^2 - N)} = \frac{\text{t}r(X - I)^T(X - I)}{2(N^2 - N)}$$

$$= \frac{\text{t}r(X^TX) - 2\text{t}r(X) + N}{2(N^2 - N)} = \frac{\text{t}r(X^TX) - N}{2(N^2 - N)} = \frac{\sum s(X) - N}{2(N^2 - N)} =: \tau(X)$$

where the functional τ takes values between 0 and $\frac{1}{2}$. Or, for a fixed N, we can test the total lower diagonal diagonal square entries of L, namely

$$\tau_N(X) := \sum_{i > j}^{N} l_{ij}^2 = \frac{1}{2}\text{tr}((X - I)^T(X - I)) = \frac{1}{2}\left(\sum s(X) - N\right) \in [0, N(N-1)/2]$$

for a single PA pair, as follows: a PA pair has a stationary yearly MIC distribution if $\tau_N(X) = N(N-1)/2$ and it has uncorrelated yearly MICs if $\tau_N(X) = 0$.

Applying these functionals to ATLAS, Supplementary Figure 8 shows that histograms of τ_N for N-year timeseries are significantly closer to $N(N-1)/2$ than to 0, consistent with non-stationary but positively correlated year-to-year MIC distributions despite all MICs being drawn from different patients. Similarly, the histogram of τ across ATLAS is biased away from zero (Supplementary Figure 8b, $p < 10^{-15}$).

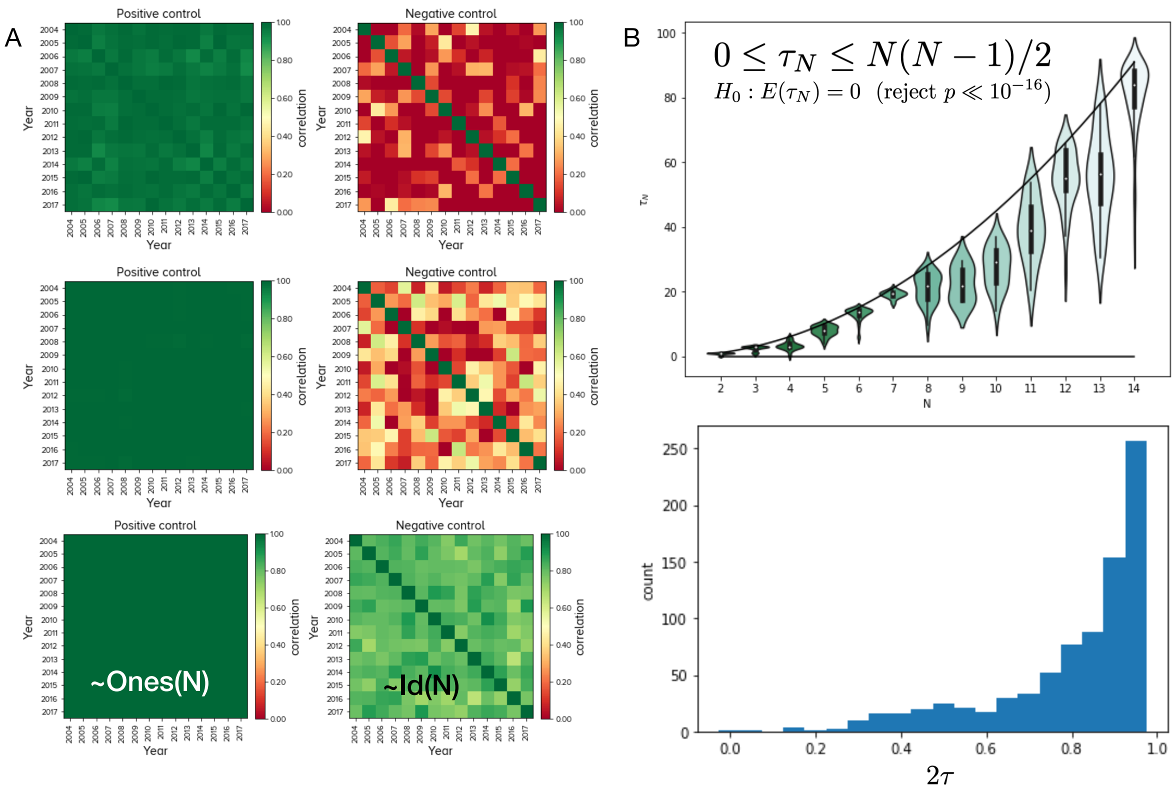

Supplementary Figure 8: Statistics of τ show ATLAS has MIC distributions with significant year-to-year correlations. A) An illustration of different correlelograms: synthetic correlation matrices were generated from timeseries both with, and without, strong temporal correlations to illustrate the near-ones (unity) matrix, Ones(N), and near-identity matrices, Id_N, that result from those different processes. B) The statistics that result from applying τ to correlation matrices for all ATLAS PA pairs that have MIC timeseries for N years where N is shown: τ lies somewhere between 0 and $N(N-1)/2$ (black lines) whereby all densities significantly are closer to the latter than the former, indicating non-random year-year MIC correlations. If we assume a null model whereby ATLAS is 'noise dominated' in the sense that MIC data for all strains and all antibiotics can be modelled as iid random variables with no between-year MIC correlations, this null can be rejected with very high probability by testing that the mean of $\tau = 0$, as the histogram of τ for all PA pairs in the lower panel illustrates (one sided t-test, p-value< 0.001). Boxplots in the top panel are standard: boxes range from lower to upper quartile, centered at the median, while the whiskers extend up to 1.5 times the interquartile range.

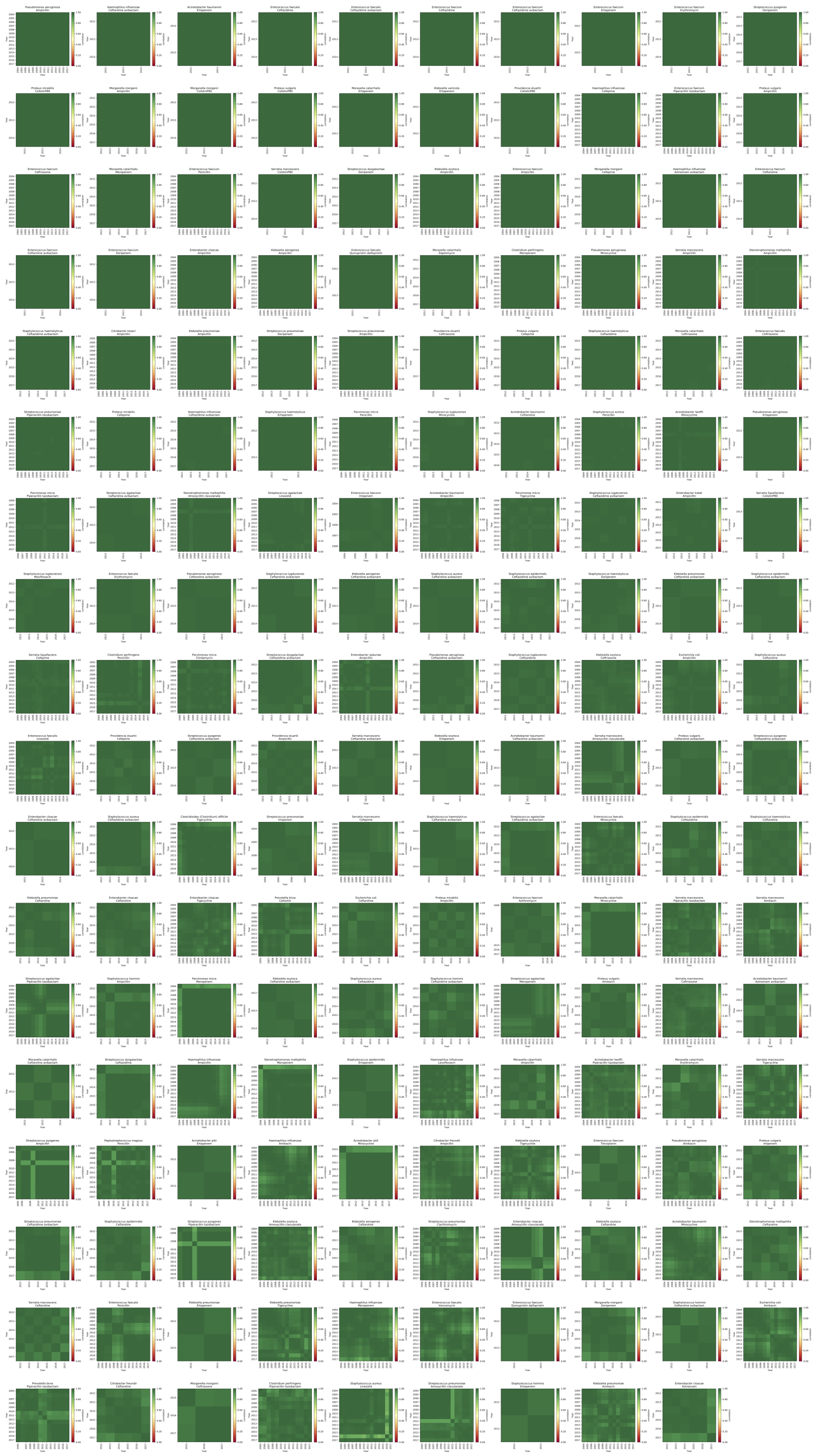

Supplementary Figure 9: PA pair year-year correlations ranked by τ (they satisfy $\tau > 0.485$). These MIC correlations are highly-ranked and are almost monochrome, consistent with slowly-varying year-year MIC distributions.

A)

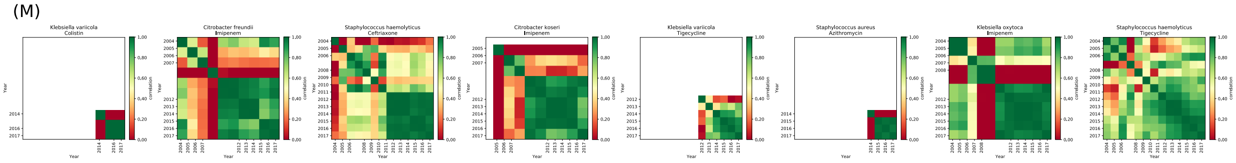
(M)
Klebsiella variicola
Colistin
Citrobacter freundii
Imipenem
Staphylococcus haemolyticus
Ceftriaxone
Citrobacter koseri
Imipenem
Klebsiella variicola
Tigecycline
Staphylococcus aureus
Azithromycin
Klebsiella oxytoca
Imipenem
Staphylococcus haemolyticus
Tigecycline

B)

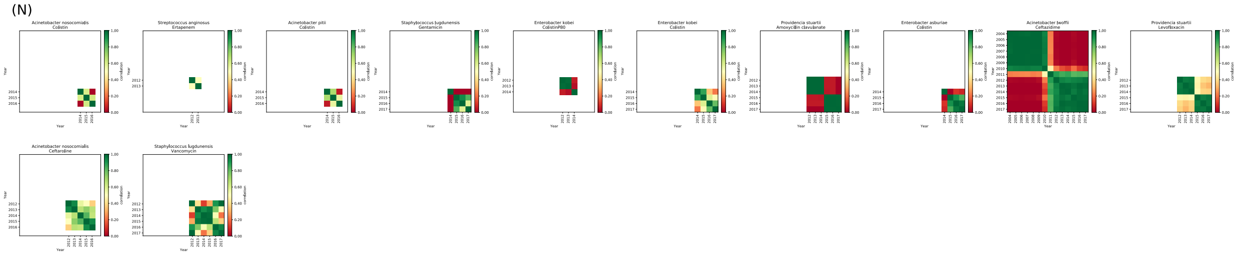
(N)

C)

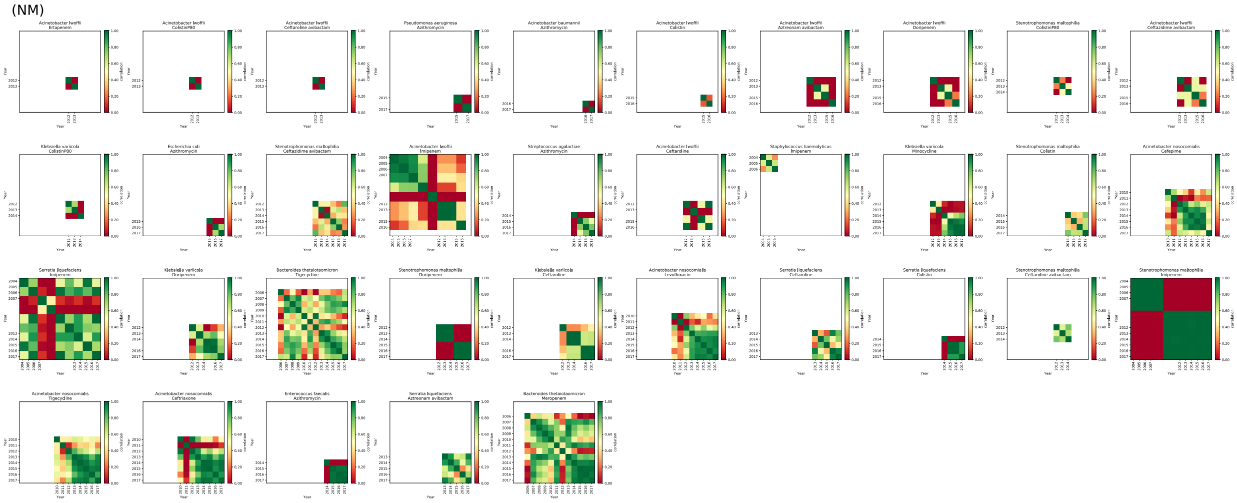
(NM)

D)

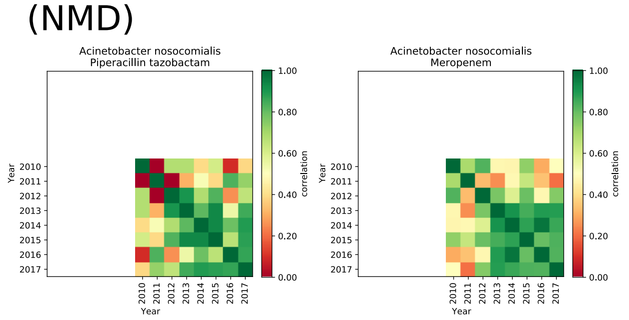
(NMD)
Acinetobacter nosocomialis
Piperacillin tazobactam
Acinetobacter nosocomialis
Meropenem
Year
Year
correlation

E)

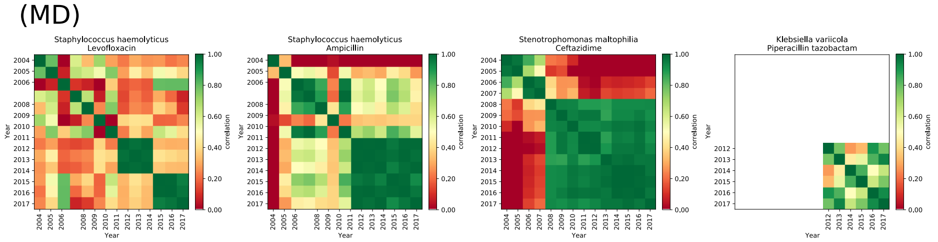

F)

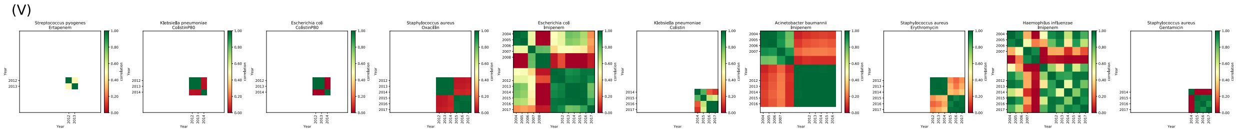

G)

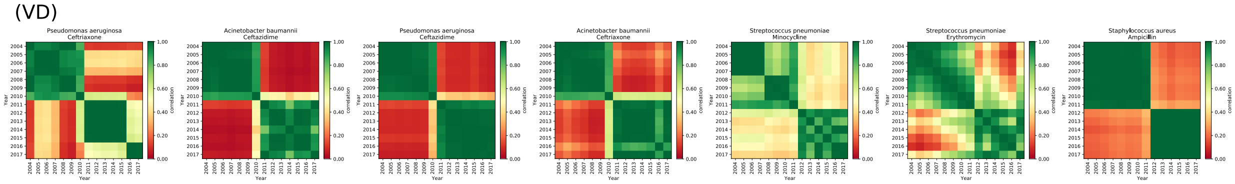

H)

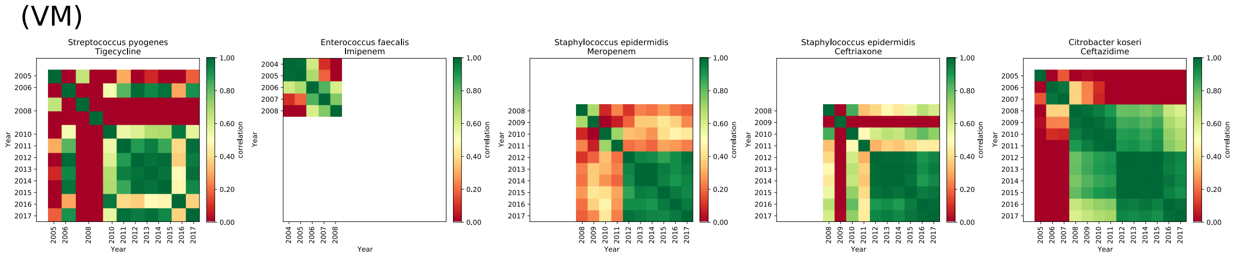

I)

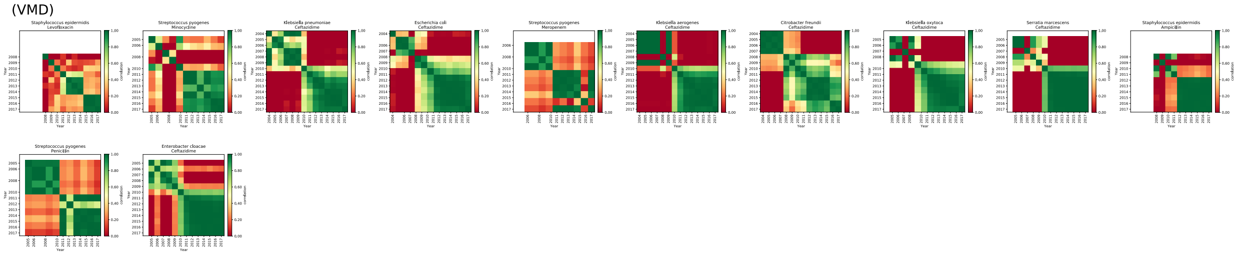

Supplementary Figure 10: This remainder of the correlelograms referred to in Figure 3 for all classification labels V, M, D, N and U, note how some PA pairs have several labels, denoting multiple potential issues with their MIC data. The data classifications described in the main text are indicated in labels placed above each sub-figure, they are repeated here: (A) M, (B) N, (C) N and M, (D) N, M and D, (E) M and D, (F) V, (G) V and D, (H) V and M, (I) V, M and D.

6 Detecting 'dissonant' PA pairs between TEST and INFORM

We discuss the PA pair *S. pneumoniae* with clindamycin and erythromycin extensively in the main text. This is because after careful consideration, we found that the abrupt shifts in the MIC distributions that occur for these PA pairs in ATLAS in 2012 were due to the amalgamation of INFORM data into the ATLAS database. We therefore wish to find out if this inconsistency between TEST and INFORM data occurs for other PA pairs, so we now present a metric-like functional to try and detect them.

Given a PA pair with data in both TEST and INFORM, we first extract the S and R clusters for each year and each database separately. Let us call the average MIC value of these clusters $S_T(y)$ and $R_T(y)$ for TEST clusters and $S_I(y)$ and $R_I(y)$ for INFORM, where y represents the year. Then, for each year y in the range 2012 to 2017 (the years in which TEST and INFORM overlap), we calculate the difference as follows:

$$\Delta = \frac{1}{6}\sum_{y=2012}^{2017} \Delta(y), \tag{4}$$

which is the average difference between years, and

$$\Delta(y) = (R_T(y) - R_I(y))^2 + (S_T(y) - S_I(y))^2,$$

as long as the four clusters exist. If both TEST and INFORM have only the R cluster for one particular year (because the clustering method has only found one cluster in the whole MIC distribution), we calculate

$$\Delta(y) = (R_T(y) - R_I(y))^2.$$

If, in one particular year, INFORM does not have an S cluster (but TEST has both R and S), we use

$$\Delta(y) = \left(\frac{R_T(y) + S_T(Y)}{2} - R_I(y)\right)^2,$$

and we use an analogous definition for when TEST does not have an S cluster but INFORM does.

Supplementary Figure 11 shows the distribution of Δ (on the y axis) versus τ for each PA pair, the latter is the measure of year-year MIC correlations defined in Section §5. Although PA pairs with high τ typically exhibit low values of Δ, there is no clearly discernible simple relationship between these variables and the outliers of the scatterplot appear at intermediate values of τ. Supplementary Figure 12 shows the four most 'dissonant' PA pairs between TEST and INFORM after the 2 *S. pneumoniae* cases.

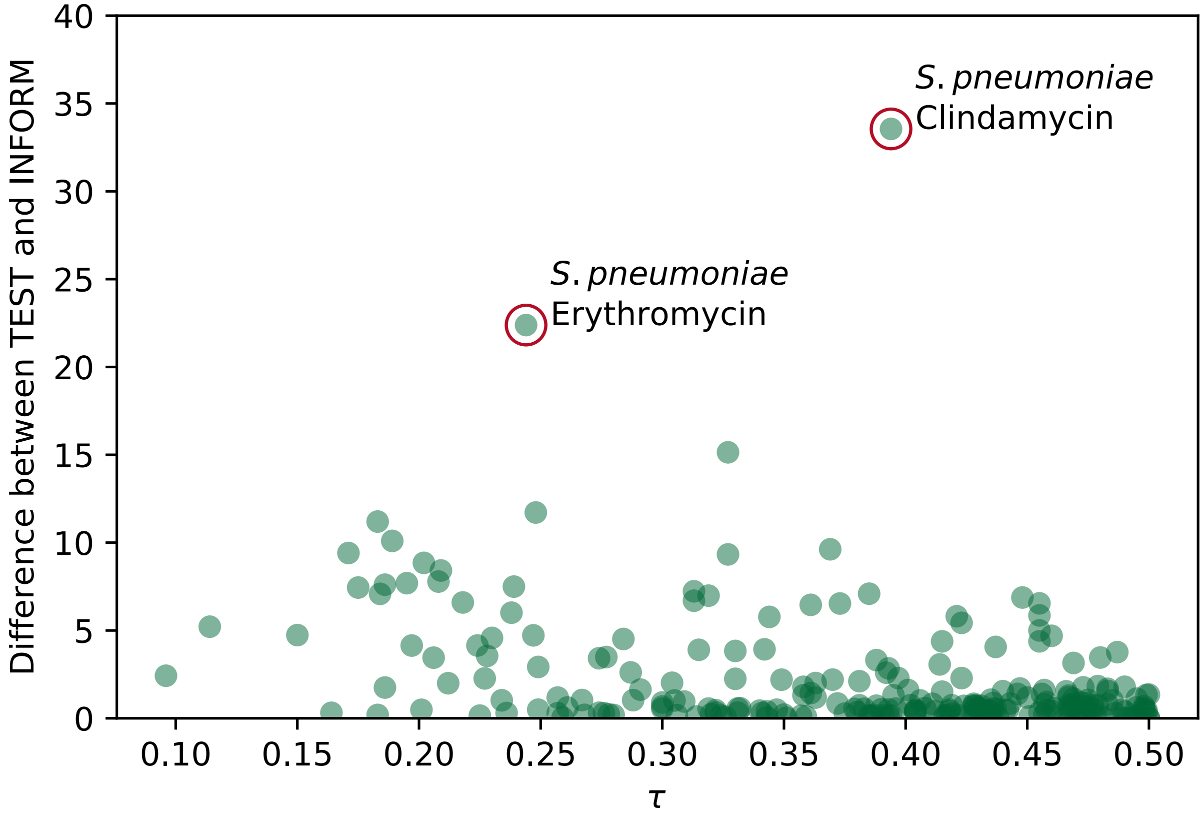

Supplementary Figure 11: Inconsistencies between TEST and INFORM. Differences between TEST and INFORM (as measured by Δ versus τ). Although most PA pairs seem to be consistent between TEST and INFORM (that is, they have low values of Δ on the y-axis), some of them exhibit very high differences between databases, particularly those indicated by a circle. Note that there is no clear correlation between Δ and τ. Marked in red are *S. pneumoniae* with clindamycin and erythromycin, the 2 PA pairs with the highest difference between TEST and INFORM databases.

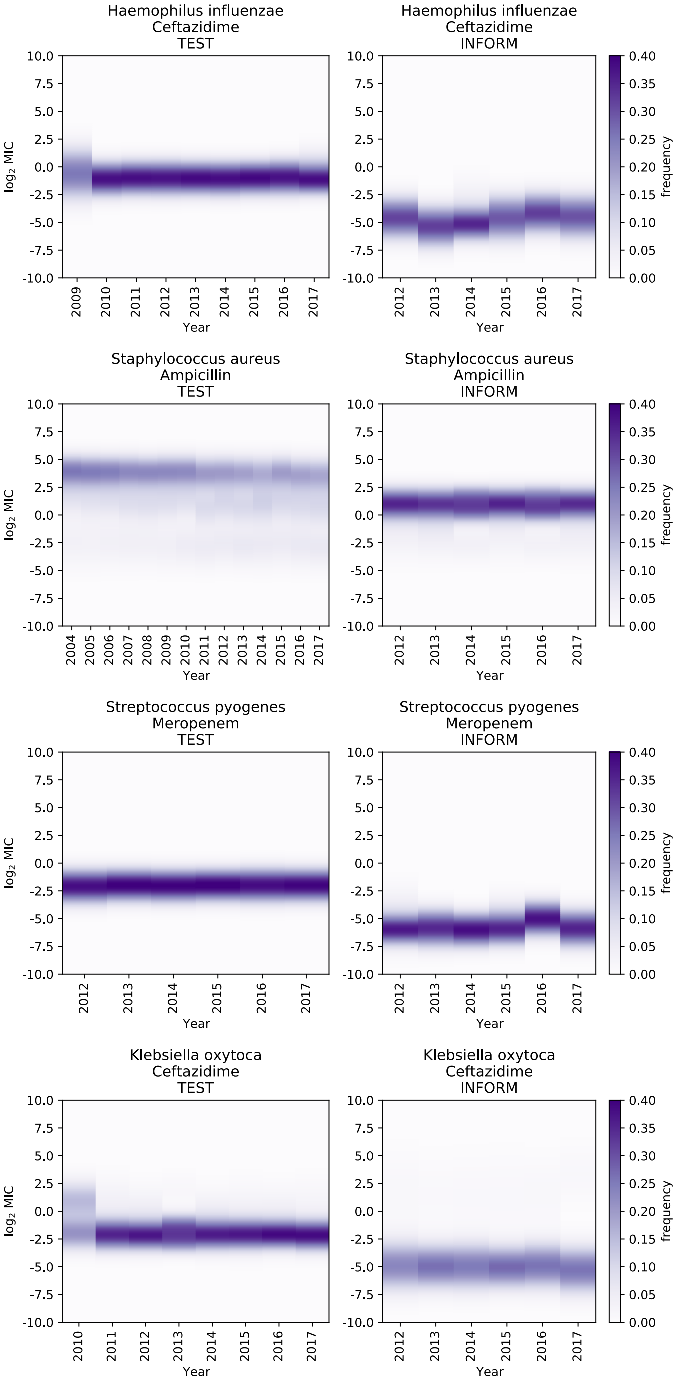

Supplementary Figure 12: PA pairs with the highest differences between TEST and INFORM. These 4 PA pairs show the highest dissonances (measured by Δ) between TEST and INFORM databases, that is, after *S. pneumoniae* and clindamycin or erythromycin. Interestingly, and as happened with the latter PA pairs as well, these 4 pairs appear consistent within a given database, it is only when comparing between two databases that a problem arises, as if different databases sample different subpopulations. However, if true, we do not know the reason for these sampling differences.

7 PA pairs with low year-year correlation, τ.

Figure 3 and Supplementary Figure 10 shows PA pairs satisfying $\tau < 1/4$. Low τ values could arise due to a number of reasons: insufficient sampling, data curation issues discussed in the main text, or the biological evolution of resistance. Seeking to clarify this, we labelled each PA pair in the figure according to the following legends:

N) If the average yearly sample size of a PA pair is below 113 (the first quartile of the distribution of this variable for all PA pairs), we label the pair with an N. This label accounts for low sampling. For instance, *K. variicola* versus ColistinP80 has an average sample size of only 2.7! Out of 128 PA pairs with $\tau < 1/4$, 49 are labelled with an N.

V) If the standard deviation of sample size between years is higher than 352 (the third quartile of the distribution of this variable for all PA pairs), we label the pair with a V. This accounts for high temporal variability in sampling size which could be a reason for low correlation between years. For instance, *S. epidermidis* versus Levofloxacin has < 15 datapoints for all years before 2012, and > 1000 after 2012. While the quality of the MIC data after 2012 is good enough to permit some analyses, we cannot compare it with data before 2012. Out of 128 PA pairs with $\tau < 1/4$, 34 are labelled with a V.

M) If the minimum sampling size across all years is lower than 7 (the first quartile of the distribution of this variable for all PA pairs), we label the pair with an M. For instance, *K. variicola* with colistin has 2 datapoints in 2014, and 200 in 2016 and 2017. Its standard deviation is not high enough to be labelled V, and it is clear that first year's dataset pushed the τ value downwards. Out of 128 PA pairs with $\tau < 1/4$, 66 are labelled with an M.

D) If the metric-like Δ defined in (4) used to test for differences between TEST and INFORM is higher than 2.3 (the third quartile of the distribution of this variable for all PA pairs), we label it with a D, accounting for PA pairs that are inconsistent between TEST and INFORM. Out of 128 PA pairs with $\tau < 1/4$, 25 are labelled with a D.

U) If the PA pair has $\tau < 1/4$ but no label among those discussed above is applicable, we label it with a U (uncertain). Out of 128 PA pairs with $\tau < 1/4$, 33 are labelled with a U and their MIC dynamics are shown in Supplementary Figure 13.

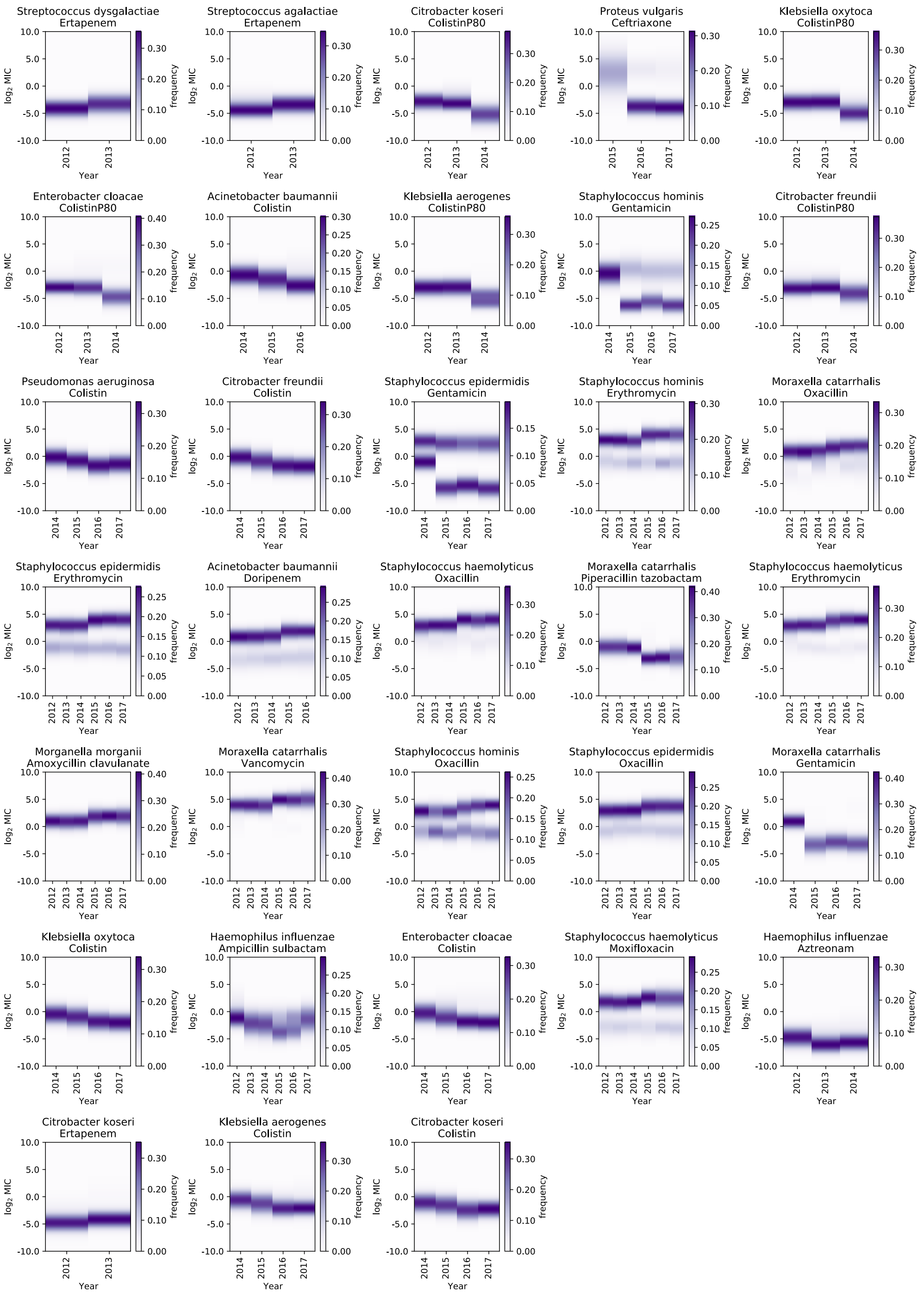

Supplementary Figure 13: PA pairs with $\tau < 1/4$ and a U classification. These 33 PA pairs do not fall under the categories N-V-M-D (see above) that could explain a low τ value and so are classified ‘U’.

8 Inconsistencies due to the revision of CLSI breakpoints

Clinical breakpoints are revised through time[29] (Table 1) and so resistance fractions reported one year might not be consistent with fractions reported after a revision which could affect comparisons between ATLAS and ECDC, ResistanceMap and ESPAUR datasets. So while ATLAS reports raw MIC data labelled with CLSI breakpoints from 2018, the other 3 databases publish resistance fractions yearly and therefore are based on the breakpoints in use in each particular year.

To quantify the variation that could accrue from this type of between-year inconsistency in reporting, we re-analysed ATLAS MIC data as if they had been used to publish resistance fractions each year, mimicking the reporting strategy of ECDC, ResistanceMap and ESPAUR. We obtained revised CLSI breakpoints from the literature[29] (see Table 1) and reanalysed the affected PA pairs.

For instance, an ATLAS *E coli* MIC value of 2.5 against Ceftazidime would be labelled susceptible in 2006 but resistant in 2014. We then re-computed the difference in reported resistance fractions between ATLAS and the other 3 databases and the results are shown in Supplementary Figure 14. ESPAUR data are not shown as the revised breakpoints did not affect comparisons with this database.

Most PA pairs show increased differences between databases when using the reanalysed data in ATLAS (Supplementary Figure 14a and c). Datapoints showing increased agreement between ATLAS and either ResistanceMap or ECDC should lie in the shaded grey area between the $y = x$ line and the x axis in these plots, but they are clearly outnumbered by the points outside this region. However, when plotting the differences between the updated/new between-database differences (y coordinates in subplots a and c) and the old/prior differences (x coordinates in subplots a and c) against sample size in ATLAS (that is, points in the grey area are negative here, everything else is positive), we see that as PA pair sample size increases in ATLAS, agreement between databases increases (Supplementary Figure 14b and d), irrespective of the use of revised breakpoints.

Supplementary Table 1: Revised CLSI breakpoints according to Humphries et al., 2019.[29] The **BP** column lists the ($\log_2$ transformed) breakpoint between susceptible and intermediate strains (this is the value used throughout the manuscript). The **Year** column lists the year until which the given breakpoint was used ('present' means the breakpoint is still in use). *Enterobacteriaceae* include *E. coli*, *Klebsiella pneumoniae*, *K. oxytoca*, *K. aerogenes* and *Enterobacter cloacae*.

Species	Antibiotic	Year	BP
Enterobacteriaceae	Aztreonam	until 2010	3
Enterobacteriaceae	Aztreonam	present	2
Enterobacteriaceae	Cefepime	until 2014	3
Enterobacteriaceae	Cefepime	present	1
Enterobacteriaceae	Ceftazidime	until 2010	3
Enterobacteriaceae	Ceftazidime	present	2
Enterobacteriaceae	Ceftriaxone	until 2010	3
Enterobacteriaceae	Ceftriaxone	present	0
Enterobacteriaceae	Ertapenem	until 2010	1
Enterobacteriaceae	Ertapenem	until 2012	-2
Enterobacteriaceae	Ertapenem	present	-1
Enterobacteriaceae	Imipenem	until 2010	2
Enterobacteriaceae	Imipenem	present	0
Enterobacteriaceae	Meropenem	until 2010	2
Enterobacteriaceae	Meropenem	present	0
Pseudomona aeruginosa	Imipenem	until 2012	2
Pseudomona aeruginosa	Imipenem	present	1
Pseudomona aeruginosa	Meropenem	until 2012	2
Pseudomona aeruginosa	Meropenem	present	1
Pseudomona aeruginosa	Piperacillin tazobactam	until 2012	6
Pseudomona aeruginosa	Piperacillin tazobactam	present	4
Acinetobacter baumannii	Imipenem	until 2014	2
Acinetobacter baumannii	Imipenem	present	1
Acinetobacter baumannii	Meropenem	until 2014	2
Acinetobacter baumannii	Meropenem	present	1

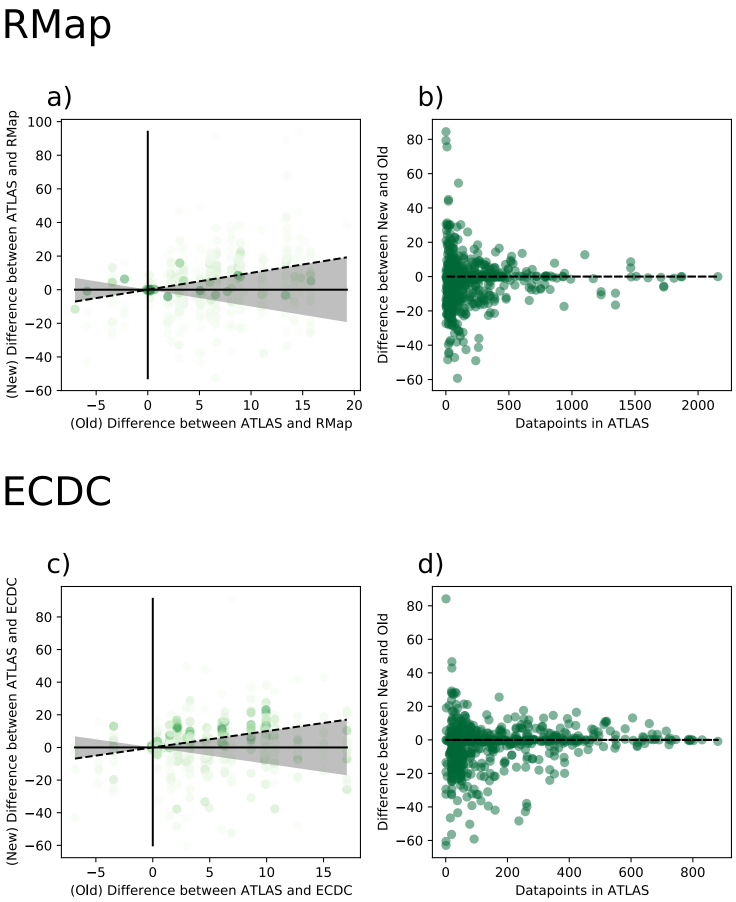

Supplementary Figure 14: Revised CLSI breakpoints affect the measured differences between ATLAS and ECDC and ResistanceMap. Reanalysed ATLAS datapoints were compared with ResistanceMap (a and b) and ECDC (c and d). Plots show one dot for each PA pair whereby the newly measured differences (in absolute value) between resistance fractions in ATLAS vs RMap (a) and ATLAS vs ECDC (c) are plotted against the old differences (note: these are as shown in Figure 1A; here green intensity is proportional to sample size in ATLAS). The dashed line $y = x$ lies where all datapoints should be if the revised breakpoints had not made any difference. Points lying in the shaded grey area between the lines $y = -x$ and the x axis show smaller differences (in absolute value) between databases than before revising the breakpoints. Note how many PA pairs lie outside this grey region, thus CLSI revisions generally increase between-database discrepancies. The latter reduce as sample sizes in ATLAS increase (b and d): when plotting the difference between the 'New' and 'Old' differences (y and x coordinates of each datapoint in a and c), the largest values (between approximately 20 and 40%) are found when sample size is small, and they tend to zero as sample sizes increase.

9 Additional figures

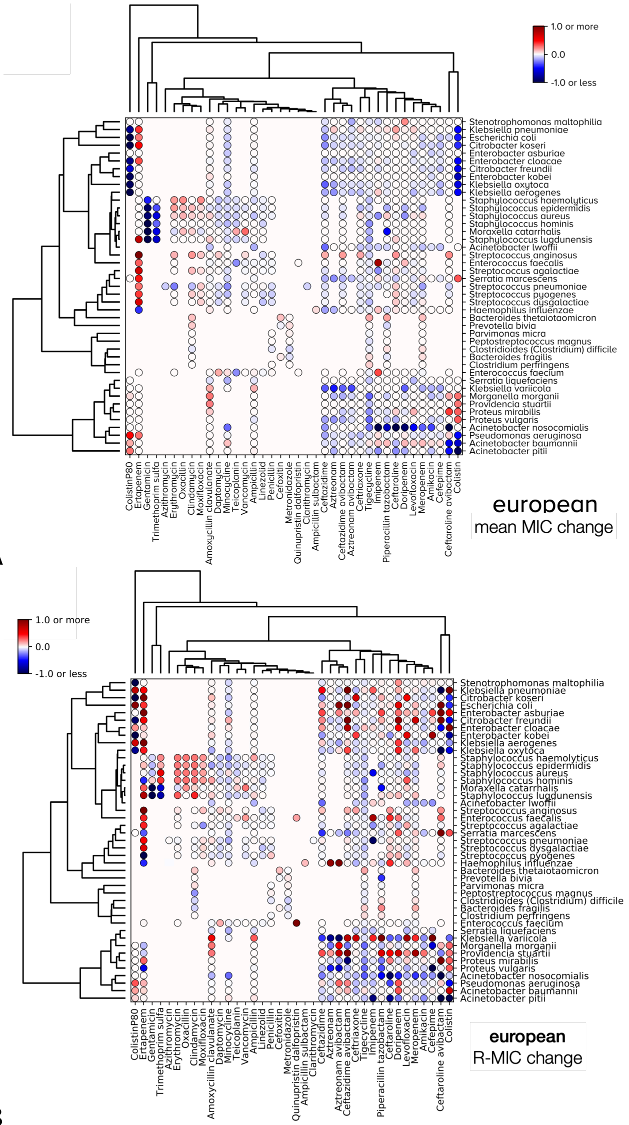

A

B

Supplementary Figure 15: Two types of predicted change for European MIC data: $\log_2$ **MIC change per year for each PA pair predicted by applying a linear regression to all MIC data in A) and just using the R-MIC in B)**. A) Linear regression applied to the global MIC distribution for each of the shown PA pairs indicates a motif of increases and decreases for Europe-wide MICs where the colour is the rate of change estimate provided by the regression slope. B) However, comparing A) with the analogous linear regressions conducted on just the R-MIC for each PA pair (again for European data) indicates a bias towards increasing MIC (red). Taken together, A and B are consistent with the observation of increases in the R-MIC of some PA pairs while the mean MIC of those pairs are decreasing.

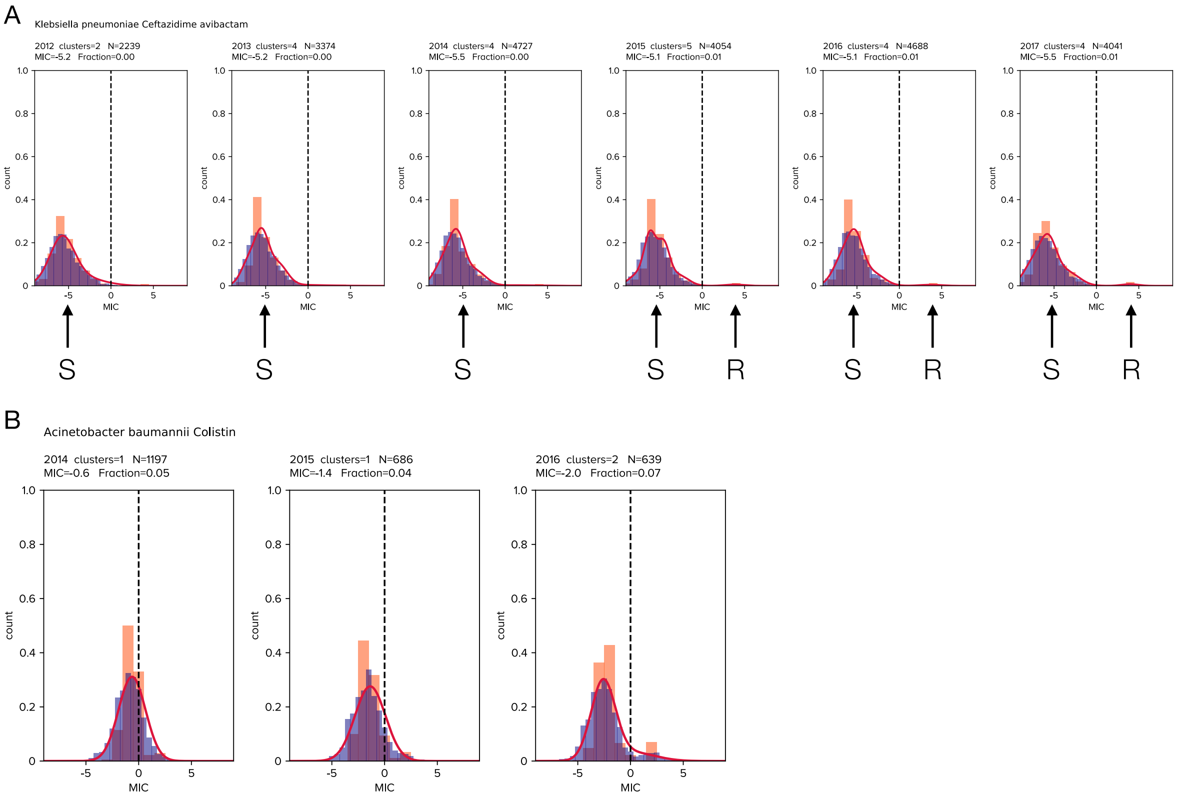

Supplementary Figure 16: Exemplar determinations of S and R cluster pairs and their changes. A) A Gaussian mixture model of the MIC distribution does not separate the *Klebsiella pneumoniae* and ceftazidime avibactam population into multiple clusters with different MIC phenotypes prior to 2015 as the most likely model is formed from significantly overlapping Gaussians. After 2015 this changes and around 1% of the population resides in a Gaussian cluster with significantly higher mean MIC than the remainder. At this time the high-MIC cluster is labelled R and the remainder is labelled S. This resembles the clinical procedure of allocating pathogens using a binary classification, S and R, based on known breakpoints however this cluster-based approach determines the R-classification breakpoint as part of an algorithmically-defined procedure applied directly to data. B) This is analogous to A but for *Acinetobacter baumanii* and collistin over a 3-year period. NB: In both, and throughout, orange bars are histograms of raw ATLAS MICs, blue histograms include modelled uncertainty with simulated noise (Methods) and red lines are estimated kernel densities determined from fitting Gaussian mixture models to those data.

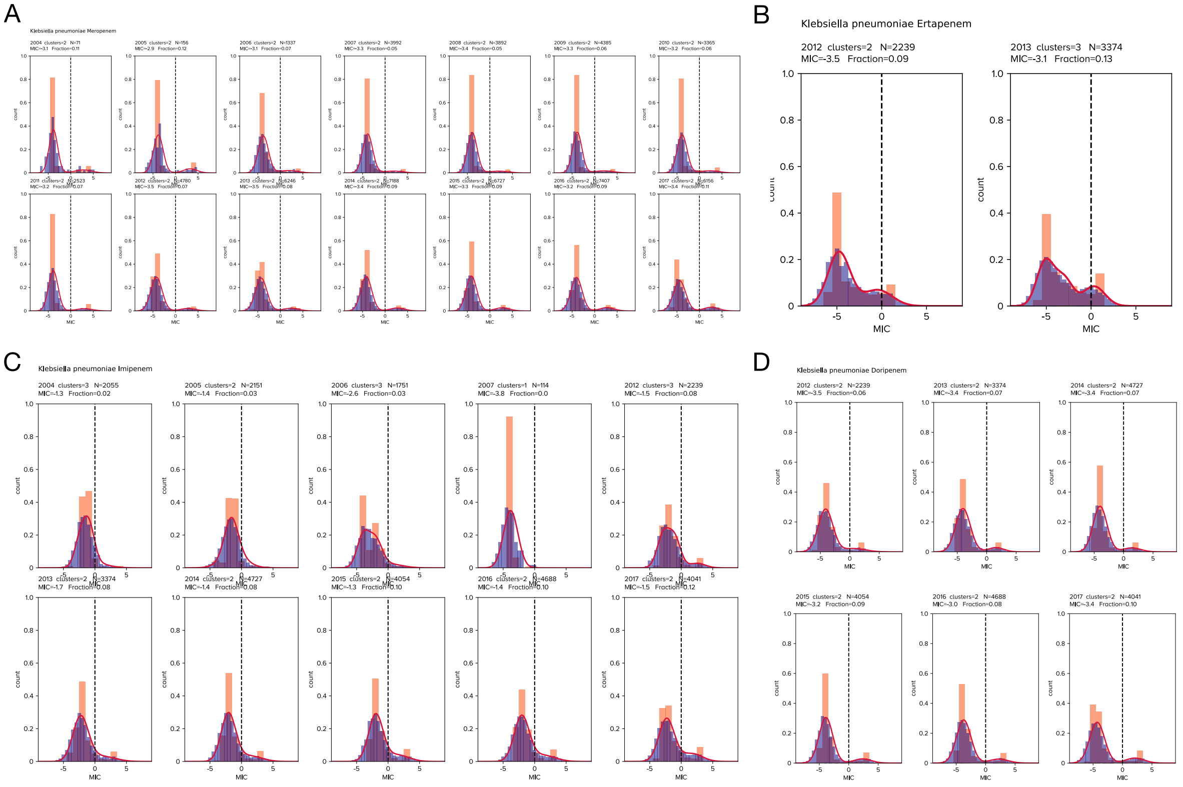

Supplementary Figure 17: Emergent and stable bimodal S- and R-clusters for *Klebsiella pneumoniae* and carbapenems. This plot is analogous to Supplementary Figure 16 whose legend contains further explanation. Antibiotics are as follows: A) meropenem ; B) imipenem - note the emergence of a clinically resistant R cluster from 2006 ; C) ertapenem ; D) doripenem. (NB: orange bars are histograms of MICs, blue histograms include modelled uncertainty and red lines are kernel densities from fitted Gaussian mixture models.)

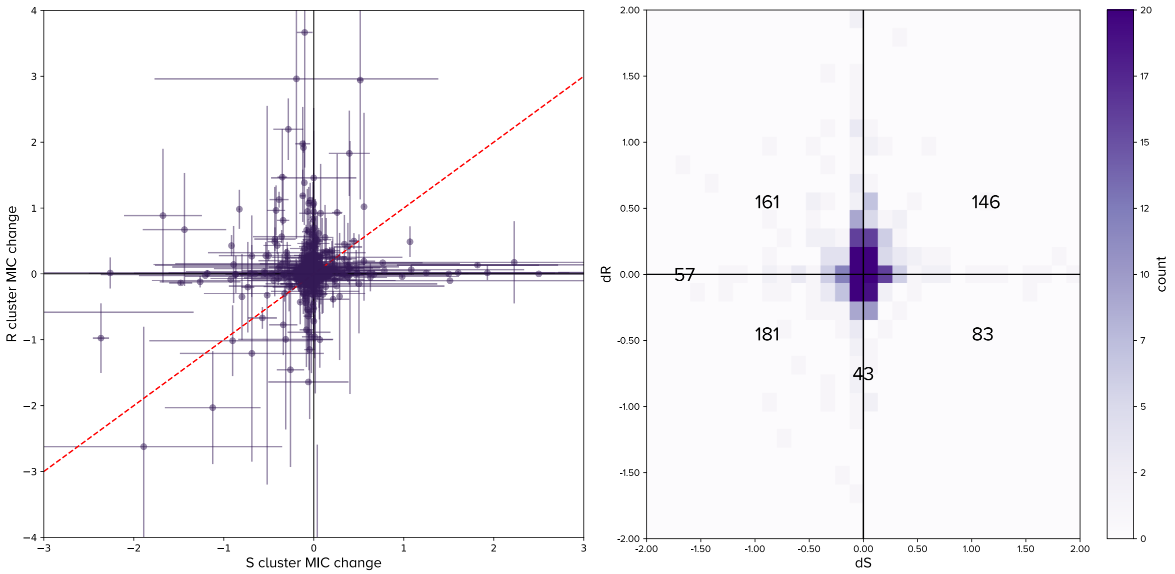

Supplementary Figure 18: The phase plane of time derivatives of the mean MICs of S and R clusters for all PA pairs in ATLAS. The rate of change of the S sub-population and the R cluster for each PA pair on the left hand plot is shown as a dot (crosshairs indicate one s.d., $n = 50$) and the right hand plot counts those on the left that reside significantly in each quadrant. It also indicates where datapoints lie on the boundaries between the 2 quadrants whereby S and R clusters are transitioning from sub- to super-breakpoint. We identify divergent MICs as being PA pairs with increasing MIC derivative in the R cluster but decreasing MIC derivative in the S sub-population, these are represented by points in the top-left quadrant. Convergent MICs arise when the MIC of S is increasing but that of R is decreasing, these are points in the bottom-right quadrant.

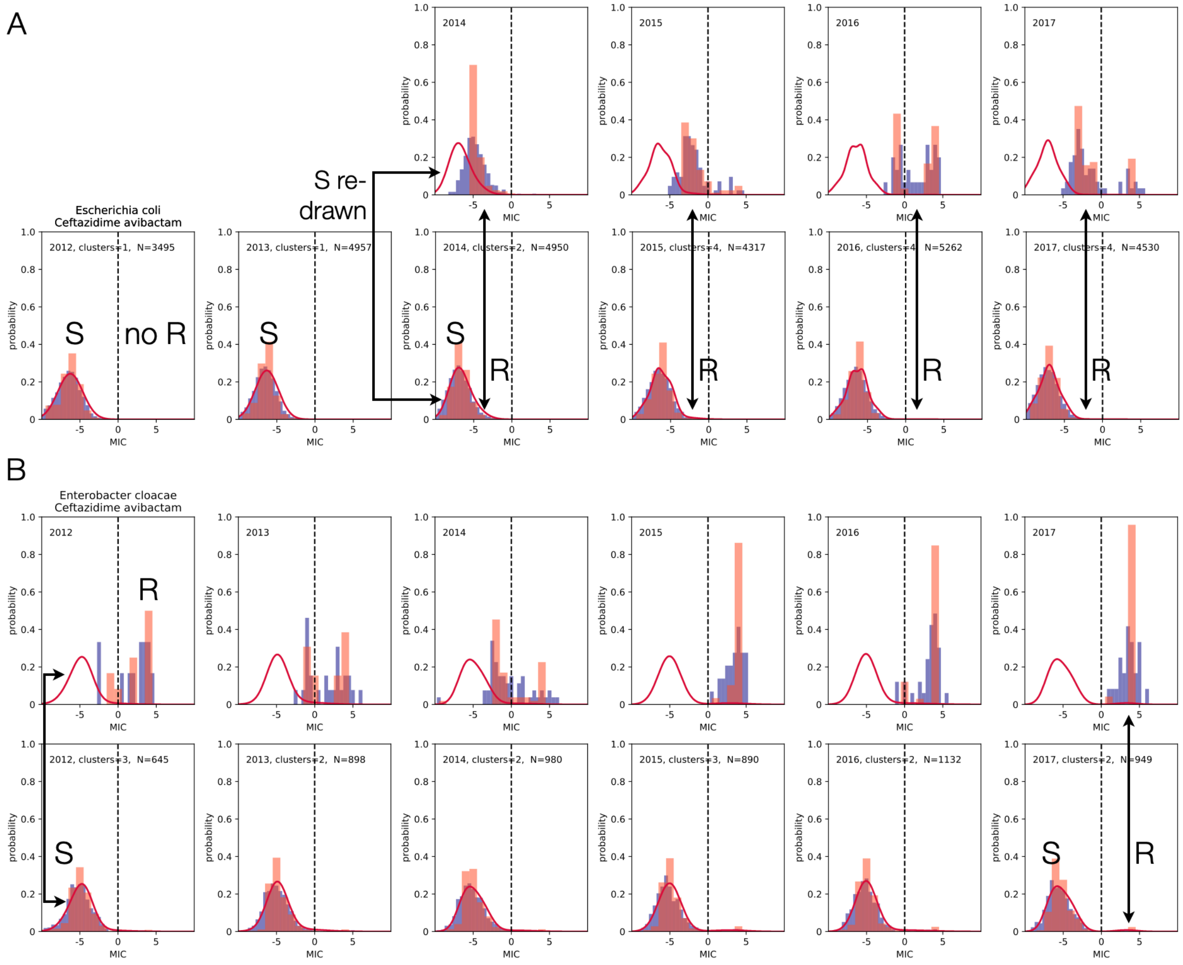

Supplementary Figure 19: Emergent and stable bimodal S- and R-clusters for A) ***E.coli*** **and CAZ-AVI, B)** ***Enterobacter cloacae*** **and CAZ-AVI.** Reading from left to right in both A and B, each panel shows the MIC distributions of the S- and R-subpopulations as years progress beyond 2012. For A, the 2012 data only has an S subpopulation (leftmost plot) and later data gains an R-cluster in 2014. This is indicated by the 2 double-headed arrows whereby the top plot in A is a zoom into the R cluster which cannot be seen in the bottom plot because the latter is dominated by S's data, making the R data invisible on the scale used. The zoom (top) shows how the R-cluster changes with time, passing from sub- to super-clinical breakpoint by 2016. For B, the plots are analogous to A except the S and R-clusters are present from 2012 onwards and the R-cluster is large enough to be seen clearly alongside the S-subpopulation by 2017. This is marked using the double-headed vertical arrow. The red lines denote the MIC frequencies of S subpopulations from below (in both A and B) that have been super-imposed onto the zooms into the R clusters above; that zoom and that red line are not plotted on the same scale, this superimposition has been done merely to guide the eye.

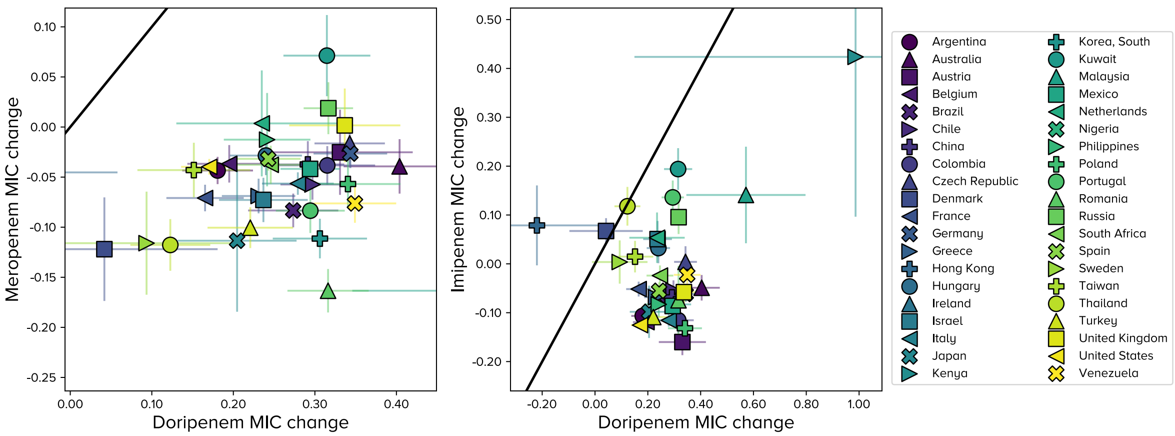

Supplementary Figure 20: Estimated time derivatives of the R-MIC for *Pseudomonas aeruginosa* and 3 carbapenems. The black line denotes '$y = x$' in both plots, thus indicating countries for which doripenem R-MICs change at the same, or different, rates as meropenem and imipenem: doripenem has a more rapidly increasing R-MIC, independently of the country analysed. Crosshairs represent s.e. of the mean, n is different for each. This analysis was performed on one replicate of the ATLAS database (Methods).

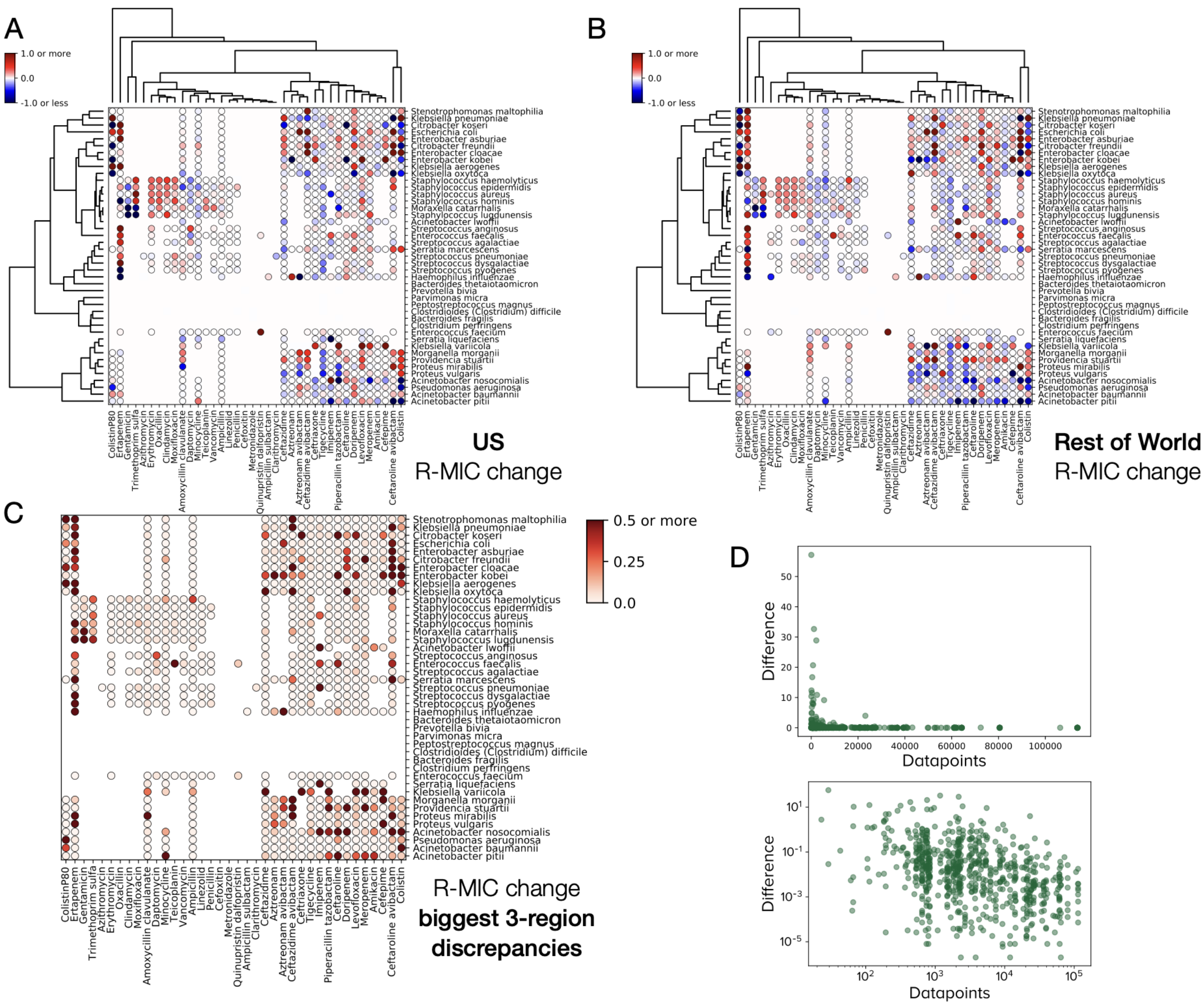

Supplementary Figure 21: Comparing R-MIC dynamics across 3 database subsets of similar size (in terms of numbers of datapoints): US, Europe and Rest of the World. A) These are regression-predicted dynamics of the R-MIC in $\log_2$ MIC change per year for all PA pairs restricted to US-only data. B) This is the same as A for the entire ATLAS database but not including US and European data (i.e. Rest of the World). For the European analogy to A and B, see Supplementary Figure 15B. C) To assess the robustness of the R-MIC methodology and of ATLAS data to make predictions on changes in resistance robustly, we compared data in A, B and Supplementary Figure 15B for each PA pair: for this, here we plot the value of $(r_1 - r_2)^2 + (r_2 - r_3)^2 + (r_3 - r_2)^2$ on a continuous colour scale where each r_i represents the predicted rate of R-MIC change for a PA pair from each of US, Europe and Rest of the World, respectively. The darker/browner points are the PA pairs that differ most in their R-MIC dynamics between these 3 regions. D) The metadata from C are plotted on the y-axis where the x-axis shows the number of available datapoints for each PA pair (one dot per pair). This shows PA pairs with bigger differences in predicted R-MIC changes have smaller datasets. (Figure 6B shows the analogy of A and B here using the entire ATLAS database.)

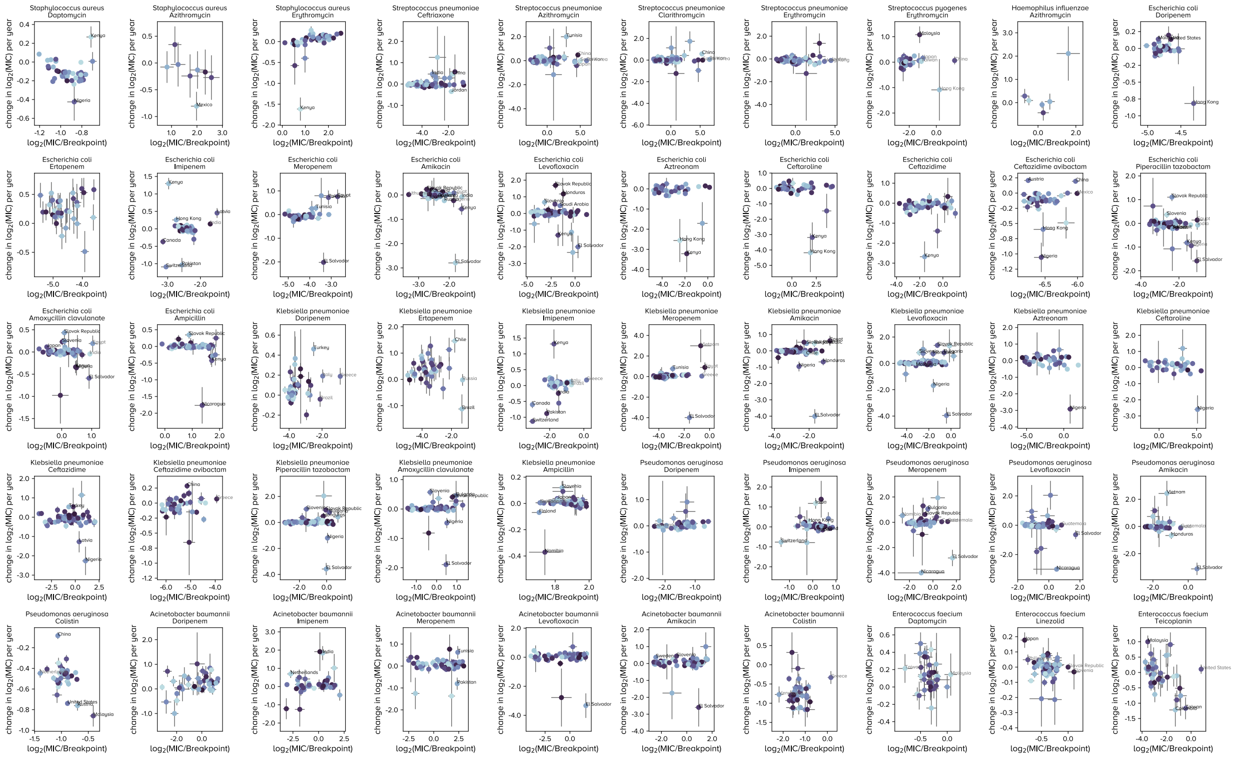

Supplementary Figure 22: Variation in MIC and change in MIC between countries. Each dot represents one country whereby the x-axis shows mean MIC and the y-axis shows a linear regression-predicted change in MIC where labels for outlier countries can be seen and bars are standard deviations. Note how some countries vary little in their MIC values for some PA pairs whereas other PA pairs can vary by several orders of magnitude. As per Methods, MICs are quoted as $\log_2$ values relative to their clinical breakpoint, so a value of zero means equal to the breakpoint.

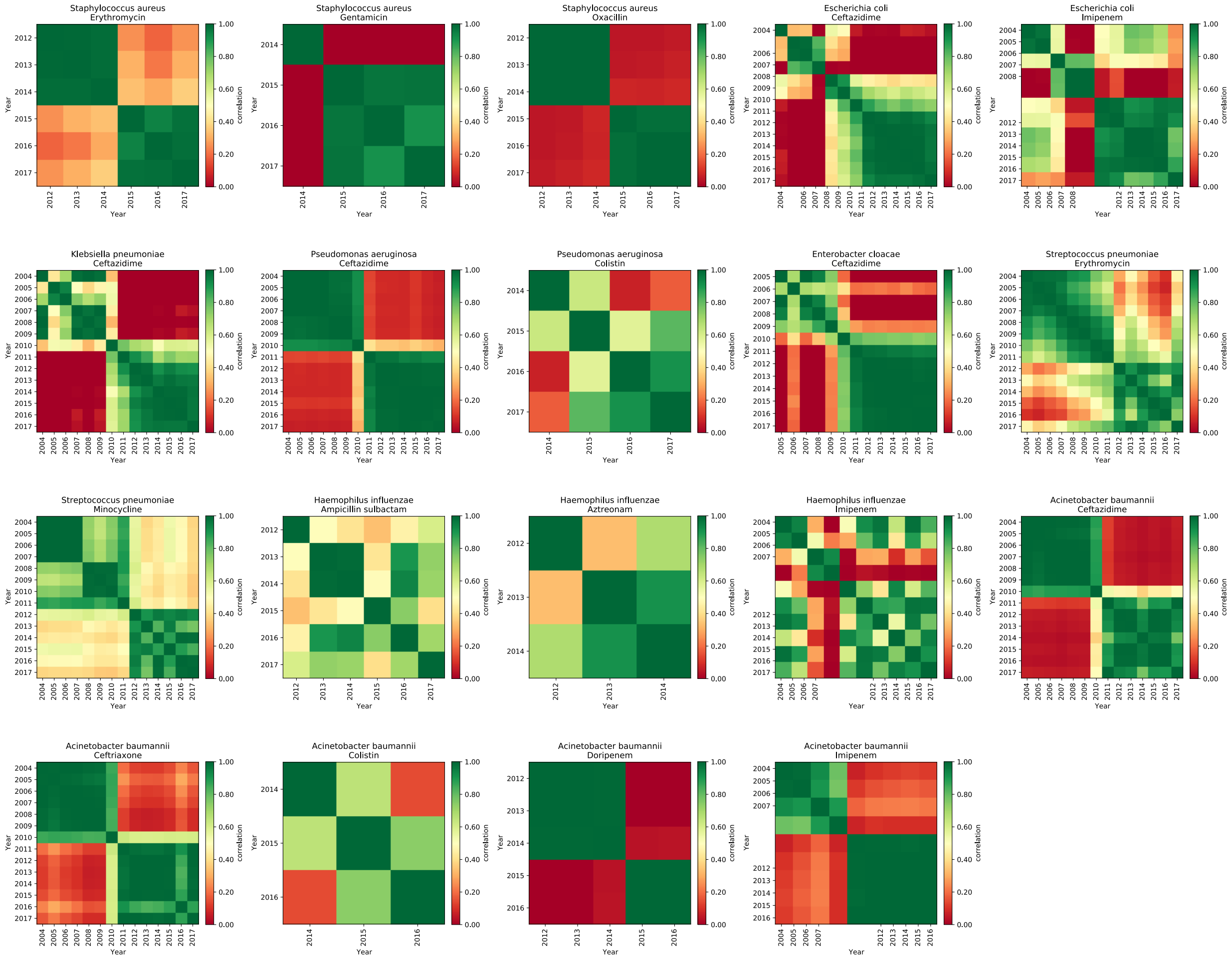

Supplementary Figure 23: PA pairs in Figure 7A with the lowest year-year correlations. These correlelograms satisfy $\tau < 1/4$.

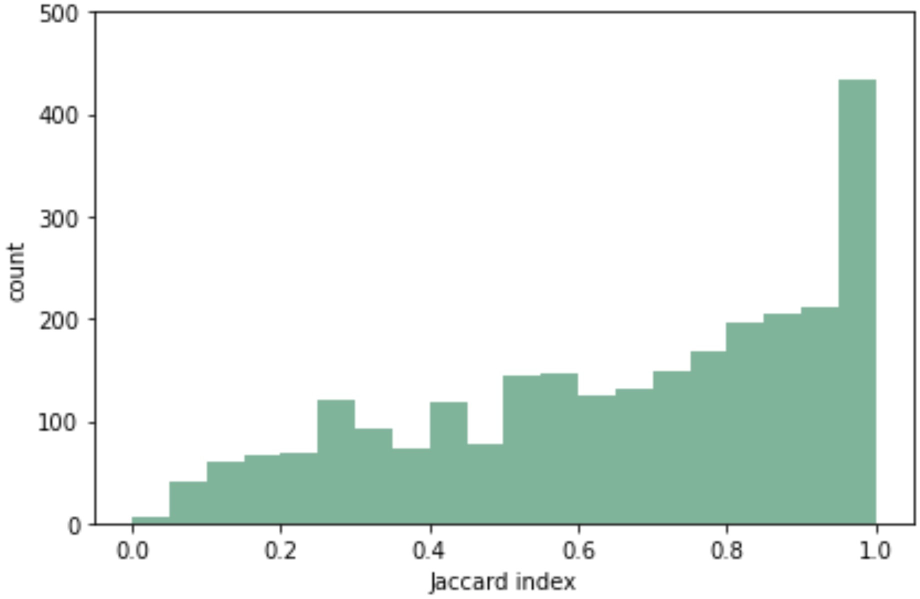

Supplementary Figure 24: Q) Do similar or different countries contribute to S and R subpopulations? This histogram shows the Jaccard index taken between S and R subpopulations for all PA pairs across ATLAs for all years, note how it skews towards unity from which we infer that most instances of S and R are constituted from similar countries (27% are below 0.5 Jaccard similarity).

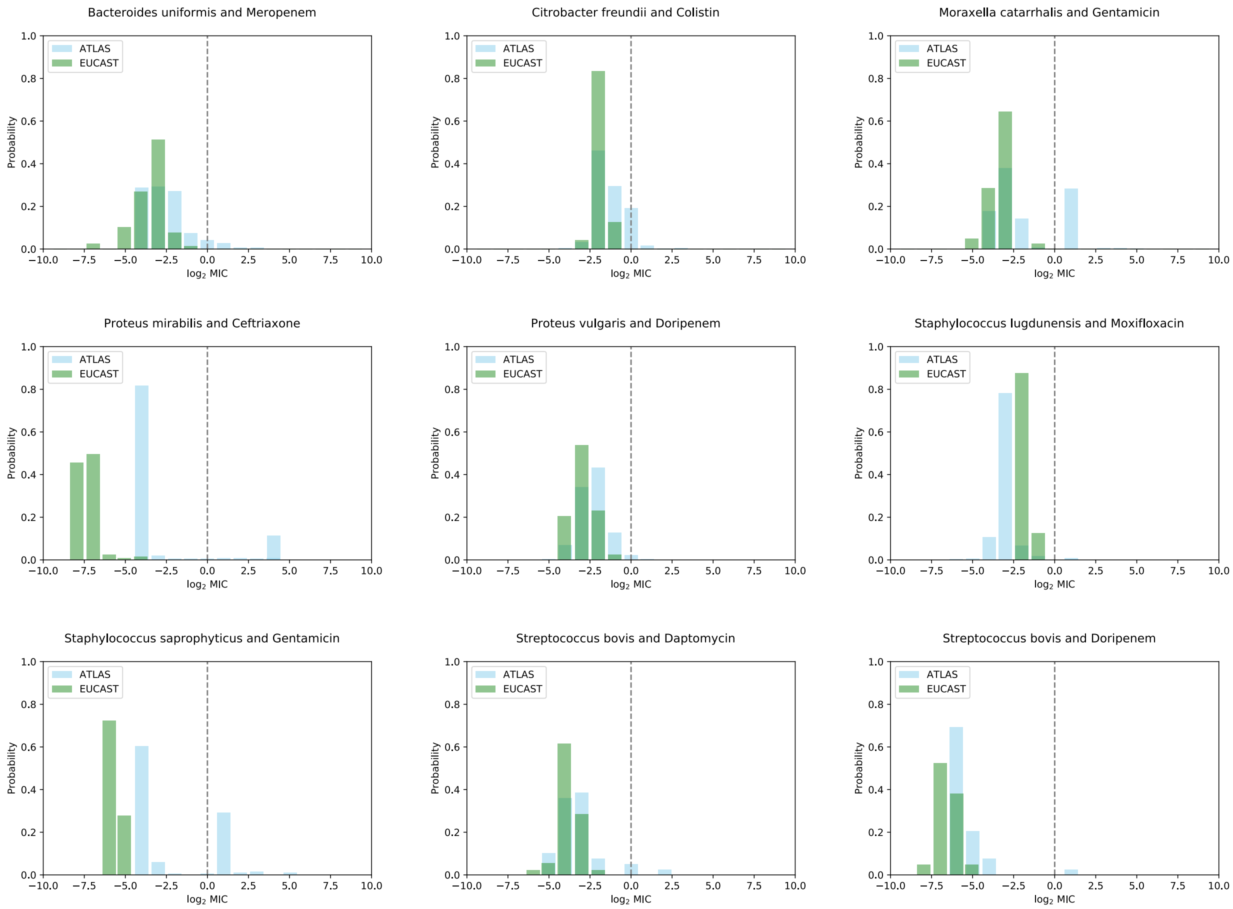

Supplementary Figure 25: ATLAS contains MIC data which indicate clinical resistance for 10 PA pairs that are not resistant according to EUCAST. The case of *S. pneumoniae* and amoxycillin clavulanate shown in Figure 2 is one of 10 antibiotic resistant PA pairs according to ATLAS for which EUCAST data does not indicate resistance, the remaining 9 cases are shown here.

10 Supplementary Tables

Supplementary Table 2: Largest frequency of resistance differences between ECDC data and ATLAS (as a %).

Species	Antibiotic	Year	Country	ECDC%	ATLAS%	Diff	N
Streptococcus pneumoniae	Macrolides[1]	2007	Latvia	0	100	100	3
Streptococcus pneumoniae	Penicillins[2]	2005	Denmark	4.44	100	95.56	1
Pseudomonas aeruginosa	Piperacillin Tazobactam	2006	Spain	8.911	100	91.089	1
Streptococcus pneumoniae	Penicillins	2005	Ireland	11.083	100	88.917	1
Pseudomonas aeruginosa	Carbapenems[3]	2006	Spain	14.396	100	85.604	1
Pseudomonas aeruginosa	Ceftazidime	2006	Spain	15	100	85	1
Streptococcus pneumoniae	Penicillins	2013	Croatia	25.862	100	74.138	1
Enterococcus faecium	Vancomycin	2016	Latvia	28.571	100	71.429	3
Streptococcus pneumoniae	Macrolides	2013	Croatia	32.759	100	67.241	3
Enterococcus faecium	Vancomycin	2014	Croatia	10.448	75	64.552	4
Enterococcus faecium	Vancomycin	2010	UK	10.4	72.7	62.3	11
Streptococcus pneumoniae	Penicillins	2008	Finland	11.059	72.2	61.141	18
Pseudomonas aeruginosa	Carbapenems	2008	Poland	18.182	78.9	60.718	19
Pseudomonas aeruginosa	Carbapenems	2011	Romania	60	0	-60	1
Streptococcus pneumoniae	Penicillins	2017	Sweden	6.133	62.5	56.367	24
Pseudomonas aeruginosa	Carbapenems	2011	Bulgaria	35.417	90	54.583	20
Pseudomonas aeruginosa	Ceftazidime	2008	Bulgaria	54.545	0	-54.545	1
Streptococcus pneumoniae	Penicillins	2014	Romania	46.667	100	53.333	4
Pseudomonas aeruginosa	Piperacillin Tazobactam	2011	Bulgaria	32.558	85	52.442	20
Pseudomonas aeruginosa	Piperacillin Tazobactam	2008	Poland	31.818	84.2	52.382	19
Pseudomonas aeruginosa	Carbapenems	2017	Latvia	71.429	21.1	-50.329	19

1. Erythromycin and Clarithromycin
2. Penicillin and Oxacillin
3. Imipenem and Meropenem

Supplementary Table 3: Largest frequency of resistance differences between RMap data and ATLAS (as a %).

**Species**	**Antibiotic**	**Year**	**Country**	**RMap%**	**ATLAS%**	**Diff**	**N**
Streptococcus pneumoniae	Macrolides[1]	2007	Latvia	0	100	100	3
Acinetobacter baumannii	Carbapenems[2]	2015	Ireland	6	100	94	1
Pseudomonas aeruginosa	Piperacillin-tazobactam	2006	Spain	9	100	91	1
Acinetobacter baumannii	Carbapenems	2012	Denmark	11	100	89	9
Pseudomonas aeruginosa	Carbapenems	2006	Spain	14	100	86	1
Pseudomonas aeruginosa	Ceftazidime	2006	Spain	15	100	85	1
Acinetobacter baumannii	Carbapenems	2014	Turkey	28	96	68	100
Streptococcus pneumoniae	Macrolides	2013	Croatia	33	100	67	3
Enterococcus faecium	Vancomycin	2014	Croatia	10	75	65	4
Enterococcus faecalis	Vancomycin	2013	Turkey	1	65.1	64.1	43
Staphylococcus aureus	Oxacillin (MRSA)	2014	Sweden	1	63.8	62.8	94
Enterococcus faecium	Vancomycin	2010	UK	10	72.7	62.7	11
Acinetobacter baumannii	Amikacin	2016	Vietnam	40	100	60	13
Staphylococcus aureus	Oxacillin (MRSA)	2013	Denmark	2	60	58	190
Staphylococcus aureus	Oxacillin (MRSA)	2015	UK	11	66.9	55.9	293
Staphylococcus aureus	Oxacillin (MRSA)	2014	Denmark	3	58.6	55.6	273
Staphylococcus aureus	Oxacillin (MRSA)	2012	Denmark	1	56.4	55.4	94
Pseudomonas aeruginosa	Carbapenems	2011	Bulgaria	35	90	55	20
Staphylococcus aureus	Oxacillin (MRSA)	2015	Germany	11	65.5	54.5	368
Staphylococcus aureus	Oxacillin (MRSA)	2014	Germany	13	67.3	54.3	490
Staphylococcus aureus	Oxacillin (MRSA)	2015	France	16	69.5	53.5	423
Staphylococcus aureus	Oxacillin (MRSA)	2012	Germany	15	67.7	52.7	300
Pseudomonas aeruginosa	Piperacillin-tazobactam	2011	Bulgaria	33	85	52	20
Staphylococcus aureus	Oxacillin (MRSA)	2015	Denmark	2	53.8	51.8	223
Staphylococcus aureus	Oxacillin (MRSA)	2013	France	17	67.8	50.8	416
Staphylococcus aureus	Oxacillin (MRSA)	2014	France	17	67.4	50.4	552
Staphylococcus aureus	Oxacillin (MRSA)	2013	Ireland	20	70.4	50.4	71

1. Erythromycin and Clarithromycin
2. Imipenem and Meropenem
