# Supplementary material for: Seeking patterns of antibiotic resistance in ATLAS, an open, raw MIC database with patient metadata
Source: Nat Commun. 2022 May 25;13:2917. doi: 10.1038/s41467-022-30635-7 (PMC9133080; doi:10.1038/s41467-022-30635-7)
Supplement: Supplementary file 2 — Reporting Summary [file 41467_2022_30635_MOESM2_ESM.pdf]

nature portfolio

Corresponding author(s): Robert Beardmore
Pablo Catalan

Last updated by author(s): Apr 6, 2022

Reporting Summary

Nature Portfolio wishes to improve the reproducibility of the work that we publish. This form provides structure for consistency and transparency in reporting. For further information on Nature Portfolio policies, see our Editorial Policies and the Editorial Policy Checklist.

Statistics

For all statistical analyses, confirm that the following items are present in the figure legend, table legend, main text, or Methods section.

n/a	Confirmed	
☒	☐	The exact sample size (*n*) for each experimental group/condition, given as a discrete number and unit of measurement
☐	☒	A statement on whether measurements were taken from distinct samples or whether the same sample was measured repeatedly
☐	☒	The statistical test(s) used AND whether they are one- or two-sided<br>*Only common tests should be described solely by name; describe more complex techniques in the Methods section.*
☒	☐	A description of all covariates tested
☐	☒	A description of any assumptions or corrections, such as tests of normality and adjustment for multiple comparisons
☐	☒	A full description of the statistical parameters including central tendency (e.g. means) or other basic estimates (e.g. regression coefficient) AND variation (e.g. standard deviation) or associated estimates of uncertainty (e.g. confidence intervals)
☐	☒	For null hypothesis testing, the test statistic (e.g. *F*, *t*, *r*) with confidence intervals, effect sizes, degrees of freedom and *P* value noted<br>*Give P values as exact values whenever suitable.*
☒	☐	For Bayesian analysis, information on the choice of priors and Markov chain Monte Carlo settings
☐	☒	For hierarchical and complex designs, identification of the appropriate level for tests and full reporting of outcomes
☒	☐	Estimates of effect sizes (e.g. Cohen's *d*, Pearson's *r*), indicating how they were calculated

Our web collection on statistics for biologists contains articles on many of the points above.

Software and code

Policy information about availability of computer code

Data collection	No code was used to collect data by the authors. The main dataset comes in the form of a very large open, pre-existing dataset (ATLAS) that we have downloaded and analysed. Datasets we have compared to that have accessing instructions - all of those are open too, except one case (called EUCAST MIC histograms) where an access request email must be sent to a European oversight committee . A large part of our paper seeks anomalies and inconsistencies in that dataset. Several url links (some of which are mirrors) to those datasets are provided. In summary, we have done no data collection but we have accessed existing data. The data availability statement in the methods provides all links to data.
Data analysis	We aim to be "fastidiously open" in terms of the analysis we have performed and all codes are openly available online. All computations in the paper were performed using Pandas 1.4.1 in Python 3 and Matlab 9.11.0.1873467 (R2021b) Update 3 including the Statistics and Machine Learning Toolbox R2021b. Stable links and DOIs (via Github and Zenodo) are provided to all the source code which functions from single user clicks.i.e. no coding ability is required to run those codes. Give the nature of the data, some of the analysis is bespoke and not based on basic "off the shelf" statistical tests. All of these deeper ideas are described in mathematical detail in the main text and the supplementary. None are particularly difficult but they do require a little specialist understanding of statistical notation, hence a good deal of pedagogical text hangs around those tests to help the non-expert reader of statistics. In terms of the replicates ("n") question above, this is important: we have no control over "n", this is a feature of the database (ATLAS) that we analyse. One key purpose of our paper is to describe exactly those elements of ATLAS whereby a low "n" value causes issues with the data, leading to potential statements that are either false or else biased. This is described in detail in the main text and is very hard to summarise here. It certainly cannot be boiled down to a single statement on whether, or not, we have described "n" values because our study addresses stratifications of ATLAS with thousands of "n" values. Finally, to address the problem that ATLAS has no replicated values in it, as it standard in the clinical practice we are addressing, we use an additive noise model to study the significance of statistical statements under synthetic ATLAS replication where n = 50 throughout.<br><br>Code availability.<br>Analysis codes written in Python 3.0 using pandas can be downloaded here:<br>https://github.com/PabloCatalan/atlas} or \url{https://doi.org/10.5281/zenodo.6390565

Codes have been written to provide straightforward access to data so that figures from this manuscript can be reproduced and to help facilitate the development of new analyses. Interested readers are encouraged to seek assistance from corresponding authors in case it is not clear how those codes are used.

For manuscripts utilizing custom algorithms or software that are central to the research but not yet described in published literature, software must be made available to editors and reviewers. We strongly encourage code deposition in a community repository (e.g. GitHub). See the Nature Portfolio guidelines for submitting code & software for further information.

Data

Policy information about availability of data

All manuscripts must include a data availability statement. This statement should provide the following information, where applicable:

- Accession codes, unique identifiers, or web links for publicly available datasets
- A description of any restrictions on data availability
- For clinical datasets or third party data, please ensure that the statement adheres to our policy

Data availability

ATLAS is available following website registration*. Data and further information can be downloaded from the following links:

Project overview: https://amr.theodi.org/project-overview

Project description: https://wellcome.ac.uk/sites/default/files/antimicrobial-resistance-surveillance-sharing-industry-data.pdf

Data download*: \url{https://www.synapse.org/#!Synapse:syn17009517/wiki/585653

The same dataset is available from this link:

https://s3-eu-west-1.amazonaws.com/amr-prototype-data/Open+Atlas_Reuse_Data.xlsx

Data was extracted from the English Surveillance Programme for Antimicrobial Utilisation and Resistance (ESPAUR) report from years 2013-2018. These were downloaded from the following UK government website: https://www.gov.uk/government/publications/english-surveillance-programme-antimicrobial-utilisation-and-resistance-espaur-report

ResistanceMap data is published by the Center for Disease, Dynamics Economics and Policy, it can be downloaded from https://github.com/gwenknight/empiricprescribing/tree/master/data

Data for the European Center for Disease Prevention and Control (ECDC) can be downloaded from
https://atlas.ecdc.europa.eu/public/index.aspx?Dataset=27&HealthTopic=4
The file we used in this paper can be downloaded from
https://github.com/PabloCatalan/atlas/tree/master/data/europe_resistance_data.csv

EUCAST data can only be obtained by contacting individuals named on their website

https://www.eucast.org/mic_distributions_and_ecoffs/

and requesting access to MIC histograms, which we were granted.

Code availability.
Analysis codes written in Python 3.0 using pandas can be downloaded here:
https://github.com/PabloCatalan/atlas} or \url{https://doi.org/10.5281/zenodo.6390565

Codes have been written to provide straightforward access to data so that figures from this manuscript can be reproduced and to help facilitate the development of new analyses. Interested readers are encouraged to seek assistance from corresponding authors in case it is not clear how those codes are used.

Field-specific reporting

Please select the one below that is the best fit for your research. If you are not sure, read the appropriate sections before making your selection.

☒ Life sciences ☐ Behavioural & social sciences ☐ Ecological, evolutionary & environmental sciences

For a reference copy of the document with all sections, see nature.com/documents/nr-reporting-summary-flat.pdf

Life sciences study design

All studies must disclose on these points even when the disclosure is negative.

Sample size

We do not disclose sample size in the sense you mean - this question as posed is not appropriate to this study. As discussed in our statistical answers above, sample sizes are not determined by us, they are already present in the ATLAS database that we study. Note also that this is a growing surveillance database, it does not have a fixed size. Moreover, the sample sizes themselves form part of the data properties that we

	study ... they are part of the problem of addressing data anomalies and biases within ATLAS. The issue here is that ATLAS is stratified into categories that could be country, disease type (infected body part), year, etc, and each stratification has its own sample size. To describe these in the text would take either pages and pages of tables, or else a figure for each stratification used.
Data exclusions	No data are excluded to begin with, but we describe stratifications of the data into infectious disease categories that may exhibit poor quality metrics and so are excluded following an analysis (having too small a sample size with $n < 3$ for a given stratification would be one obvious reason to exclude data). This information is described in detail as it is a core part of the study both in the main text in several places but also in the supplementary
Replication	This is a study of a single database with no replication, this is standard clinical practice in this field because each patient is assessed once and only once. Thus each datum in that database is not replicated either. This clinical practice is a problem over which we have no control. In order to remedy the statistical shortcomings this creates, we have a synthetic replication model for the entire ATLAS database based on synthetic additive noise based on some empirically motivated assumptions for which $n = 50$ throughout.
Randomization	There is no randomisation procedure because of the nature of ATLAS which is described in detail in the text. Rather, each patient that is presented to clinic with an infection is included in this surveillance database and their clinical data is annotated with metadata based on their symptoms. In this context, the term "randomisation" has no meaning as those symptoms and a subsequent diagnosis dictate which disease group each patient is allocated to. (There are no treatment groups, for example.)
Blinding	There is no group allocation and hence no blinding, but all the data analysed is anonymous.

Reporting for specific materials, systems and methods

We require information from authors about some types of materials, experimental systems and methods used in many studies. Here, indicate whether each material, system or method listed is relevant to your study. If you are not sure if a list item applies to your research, read the appropriate section before selecting a response.

Materials & experimental systems

n/a	Involved in the study
☒	☐ Antibodies
☒	☐ Eukaryotic cell lines
☒	☐ Palaeontology and archaeology
☒	☐ Animals and other organisms
☒	☐ Human research participants
☒	☐ Clinical data
☒	☐ Dual use research of concern

Methods

n/a	Involved in the study
☒	☐ ChIP-seq
☒	☐ Flow cytometry
☒	☐ MRI-based neuroimaging
